# Rotamer-Controlled Dual Emissive α-Amino Acids

**DOI:** 10.1021/acs.orglett.3c02112

**Published:** 2023-07-28

**Authors:** Rochelle McGrory, Danielle C. Morgan, Andrew G. Jamieson, Andrew Sutherland

**Affiliations:** † School of Chemistry, The Joseph Black Building, University of Glasgow, Glasgow G12 8QQ, United Kingdom; ‡ School of Chemistry, Advanced Research Centre, University of Glasgow, Glasgow G11 6EW, United Kingdom

## Abstract

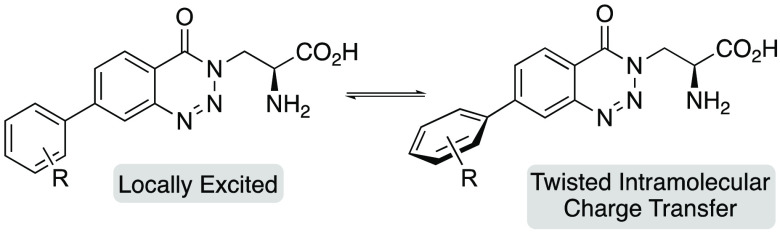

The synthesis and photoluminescent properties of novel α-amino acids are described in which the biaryl benzotriazinone-containing chromophores were found to display dual emission fluorescence via locally excited (LE) and twisted intramolecular charge transfer (TICT) states. The intensity of each emission band could be controlled by the electronics and position of the substituents, and this led to the design of a 2-methoxyphenyl analogue that, due to twisting, displayed bright TICT fluorescence, solvatochromism, and pH sensitivity.

Fluorescent spectroscopy has become a powerful technique for noninvasive imaging of dynamic molecular events in living organisms.^1^ In combination with small-molecule fluorescent dyes that are easily prepared and have tunable optical properties, the use of fluorescent spectroscopy has attracted broad interest for biomedical imaging.^2^ This is particularly the case for α-amino acids. As proteinogenic α-amino acids, phenylalanine, tyrosine, and tryptophan have suboptimal fluorescent properties, this has resulted in the design and discovery of new structures, derived from either natural amino acids or compounds with novel side chain chromophores.^3^ This approach has allowed the development of unnatural fluorescent α-amino acids with tunable photoluminescent properties that can be incorporated into peptides and proteins by using techniques such as solid phase peptide synthesis (SPPS) or unnatural amino acid mutagenesis. Notable examples include l-tryptophan-BODIPY conjugate **1**,^4^ incorporated into a cyclic peptide and used to visualize fungal infections in human tissue and the environment-sensitive dimethylaminophthalimide **2** that was used for sensing dynamic protein–protein interactions (Figure 1a).^5^ Recently, benzoselenadiazole-derived amino acids such as **3** were found to be photostable and applicable for imaging of synaptosomes in mouse brain tissue.^6^

**Figure 1:**
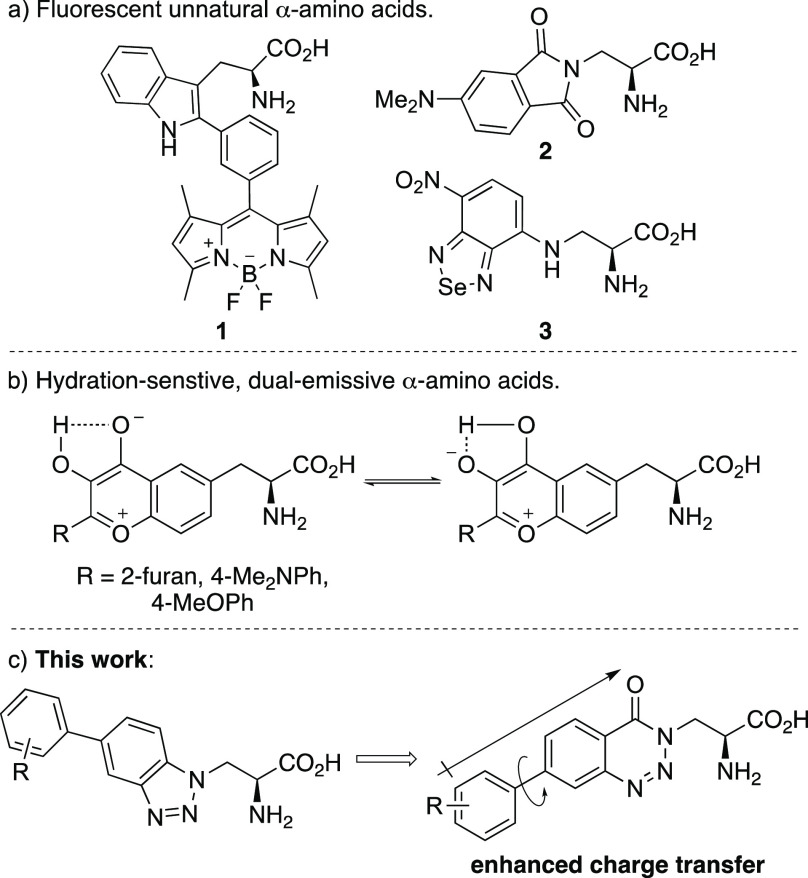
Fluorescent unnatural α-amino acids.

Dual emission fluorescence is a two-color emission process and in small-molecule dyes generally occurs via two distinct excited electronic states.^7^ Although uncommon due to the fast relaxation process associated with fluorescence, in the past decade, dual emission fluorescent molecules have been developed for a range of material and biological applications, such as pH and ion sensors, as organic light emitting diodes and in distinguishing enzyme binding sites.^8^ Fluorescent unnatural α-amino acids that display dual emission are relatively uncommon.^9^ Several examples were reported by Mély and co-workers who demonstrated that flavone-derived α-amino acids possessed dual emission fluorescence arising from excited state intramolecular proton transfer (Figure 1b).^10^ These α-amino acids were incorporated into peptides and used to study binding with oligonucleotides and peptide orientation in lipid bilayers.

Dual emission can occur via a range of different mechanisms.^7^ Upon excitation of biaryl compounds that contain flexible charge-transfer π-conjugated chromophores, dual emission often occurs by initial relaxation to a planar structure with partial charge transfer character known as the locally excited state (LE).^7,8^ Further relaxation to a more twisted structure with enhanced charge transfer, the twisted intramolecular charge transfer (TICT) state, results in a second emission band. Based on this, we believed that α-amino acids containing biaryl side chains with restricted rotation may display dual emission fluorescence via LE and TICT excited states. We recently reported the synthesis and development of several new classes of unnatural α-amino acids,^11^ including compounds with biaryl benzotriazole-derived side chains that displayed charge transfer fluorescence (Figure 1c).^12^ To achieve dual emission via LE and TICT states, we proposed that modification of the benzotriazole^13^ side chain to a benzotriazinone motif would allow both flexibility and enhanced charge transfer properties. The motivation for developing dual emission amino acids of this nature was the potential to use the different sensitivities of the LE and TICT bands to polarity and pH to report environmental changes. Furthermore, amino acids in which the relative intensity of the two emission bands is controlled by the conformation of the biaryl side chain could also provide detailed information on protein active site binding. Herein, we report the synthesis and photoluminescent properties of benzotriazinone-derived α-amino acids. As well as demonstrating substituent control of LE or TICT emission, we report the polarity and pH sensitivity of the brightest amino acid and its incorporation into a cell-penetrating peptide.

The proposed synthesis of benzotriazinone-derived α-amino acids involved three key disconnections (Scheme 1). Late-stage diversity would be introduced to study the electronics and structure of the chromophore via a Suzuki–Miyaura reaction of a 7-bromobenzotriazinone intermediate and various arylboronic acids. The key 7-bromobenzotriazinone motif would be prepared by a one-pot diazotization and cyclization of a 2-aminobenzamide, which would be readily synthesized by acylation of an l-3-aminoalanine derivative.

**Scheme 1:**
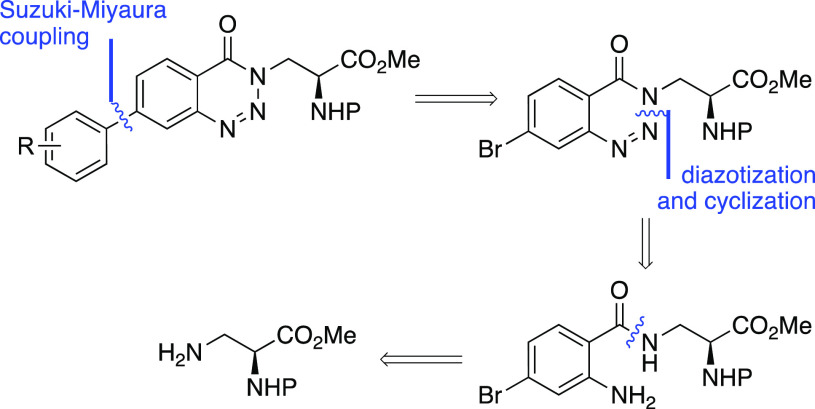
Proposed Synthesis of Benzotriazinone Amino Acids

The synthesis of the benzotriazinone α-amino acids is summarized in Scheme 2. Acid chloride **5** was prepared by the reaction of 2-nitro-4-bromobenzoic acid with thionyl chloride, and this was then reacted in situ with l-3-aminoalanine derivative **4**,^14^ to give the benzamide intermediate **6** in 57% yield over the two steps. Nitro-group reduction using zinc and acetic acid under mild conditions gave the corresponding amine **7** in 91% yield. Synthesis of key benzotriazinone intermediate **8** was then achieved in 92% yield using a polymer-supported nitrite reagent and *p*-tosic acid.^15^ This reaction generates a stable diazonium tosylate salt which undergoes in situ cyclization.^16^ Arenes with different electronic characteristics were then incorporated using a Suzuki–Miyaura reaction.^17^ The use of the Buchwald precatalyst XPhos Pd G2 at 2 mol % loading allowed fast reactions (1 h) under mild conditions (40 °C) and gave the coupled products in 68–95% yields.^18^ Mild conditions were also employed for two-step deprotection. Ester hydrolysis using cesium carbonate at room temperature, followed by acid-mediated removal of the Boc-group, gave, after recrystallization, the target α-amino acids in good overall yields.

**Scheme 2:**
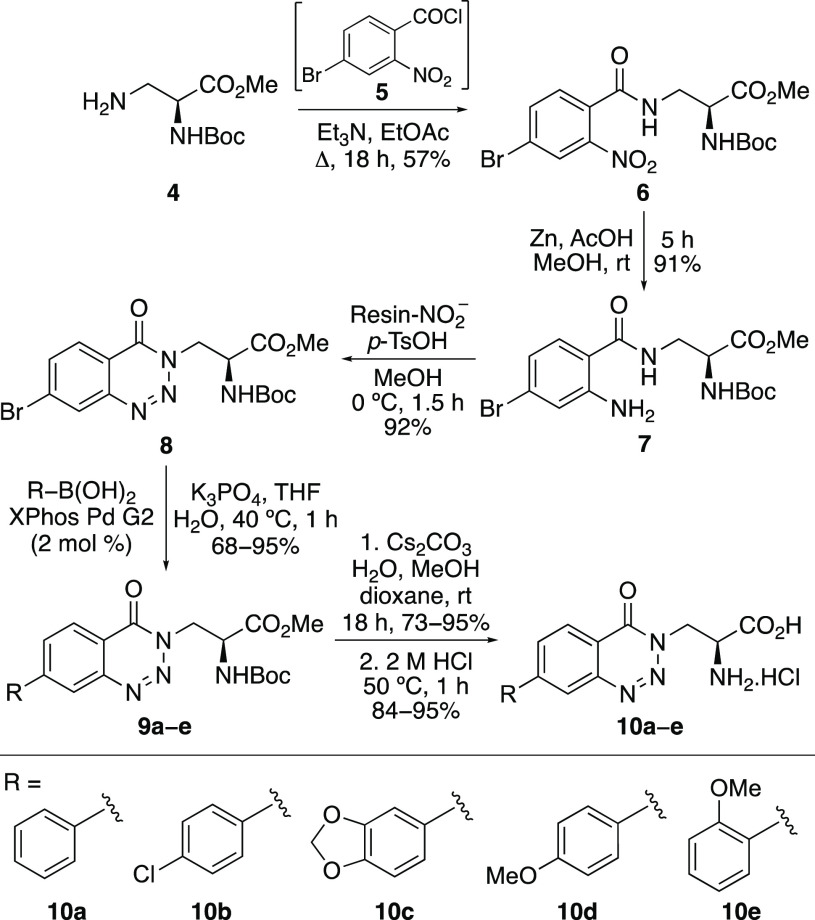
Synthesis of α-Amino Acids **10a**–**e**

The photoluminescent properties of α-amino acids **10a**–**e** were then measured (Table 1 and Supporting Information). Phenyl analogue **10a** showed weak dual-emission fluorescence with a strong LE band at 308 nm and a faint TICT band at 426 nm (Figure 2).^19^ As expected, the 4-chlorophenyl compound **10b** also displayed weak dual-emission fluorescence. However, with the introduction of the chloride substituent resulting in greater twisting of the biaryl side chain, the TICT band was found to be stronger than that for **10a**. Electron-rich analogues, benzodioxole **10c** and 4-methoxyphenyl **10d**, with enhanced charge transfer properties continued the trend of stronger TICT bands. For amino acid **10d**, which showed strong fluorescence, with a quantum yield of 0.13, TICT was found to be the major emission pathway. These results then led to the design of the 2-methoxyphenyl analogue **10e**. It was proposed that an electron-rich *ortho*-substituent would maximize twisting of the biaryl system, resulting in the suppression of LE emission and enhancement of the TICT excited state. This theory was confirmed by the emission spectrum of **10e**, which showed a greatly reduced LE band at 312 nm and a strong TICT band at 395 nm. As well as red-shifted absorbance, **10e** was found to possess the strongest brightness with a quantum yield of 0.47. Collectively, these results demonstrate the tuning of photoluminescent properties by substituent-controlled rotation of the biaryl side chain.

**Table 1:** Photophysical Data of α-Amino Acids **10a**–**e**

amino acid	λ_Abs_ (nm)^a^	ε (cm^–1^ M^–1^)	λ_Em_ (nm)^a^^,^^b^	Φ_F_^c^	brightness (cm^–1^ M^–1^)
**10a**	266	23 900	**308**, 426	0.005	110
**10b**	270	21 400	**308**, 435	0.003	60
**10c**	279	15 500	**312**, 435	0.008	130
**10d**	269	10 600	310, **388**	0.13	1400
**10e**	309	18 200	312, **395**	0.47	8540

a Spectra were recorded at 15 μM in methanol.

b Bold format represents major emission band.

c Quantum yields (Φ_F_) were determined in methanol using anthracene and L-tryptophan as standards.

**Figure 2:**
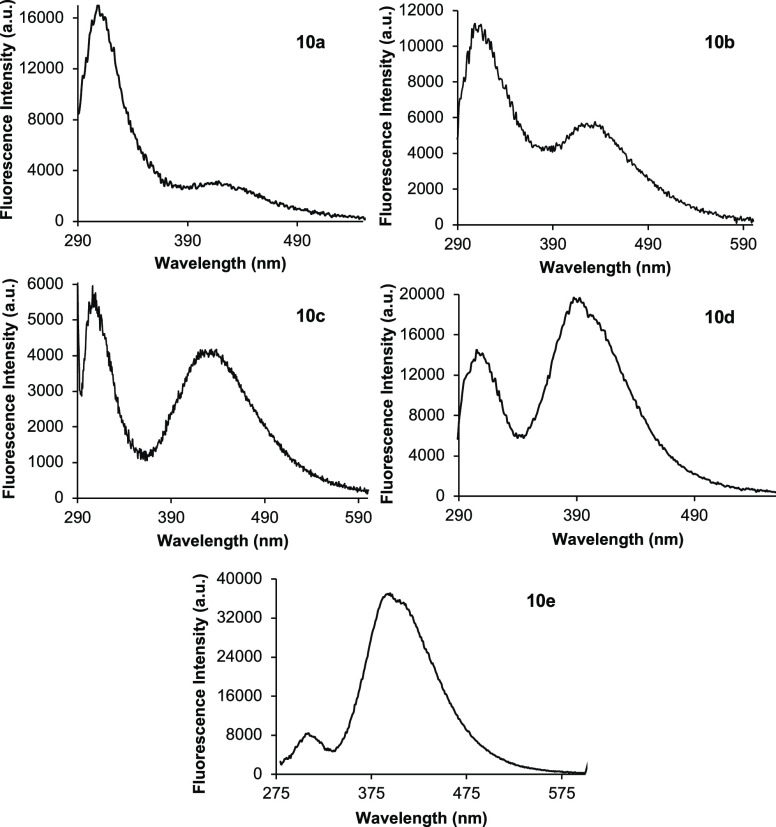
Emission spectra of α-amino acids **10a**–**e**.

Having identified lead α-amino acid **10e** through structural analysis of the biaryl system, we further explored the photoluminescent properties of this compound via solvatochromic and pH studies. In various solvents, **10e** demonstrated similar absorption maxima (Supporting Information), suggesting that absorbance is independent of solvent polarity in the ground state. In contrast, the emission spectra displayed a significant bathochromic shift, directly correlated with increasing polarity (Figure 3a).^20^ The emission maximum was found at 310 nm in THF, compared to 432 nm in phosphate-buffered saline (PBS). As expected, the TICT band was most affected by a change in polarity. Comparison of emission spectra in DMSO and PBS showed a difference of 20 nm between the LE bands, while this increased to 59 nm for the TICT bands. These results confirm the internal charge transfer character of the main emission band, which is stabilized in more polar solvents. Next, the effect of pH variation on fluorescence was explored (Figure 3b).^21^ Minimal changes were observed with decreasing pH from 7 to 4. At pH 1, while similar intensity was observed for the LE band, there was an ∼3-fold reduction in the intensity of the TICT band. Under these strongly acidic conditions, we propose that protonation of the benzotriazinone ring results in reversible ring-opening, which explains the pH sensitivity of **10e** (Scheme 3). Ring-opening of the triazinone ring would allow rotation of the biaryl system with respect to the electron-withdrawing amide moiety, resulting in the disruption of charge transfer and suppression of the TICT band. Overall, the combined polarity- and pH-dependent fluorescence of α-amino acid **10e** suggests potential as a chemical biology probe.

**Figure 3:**
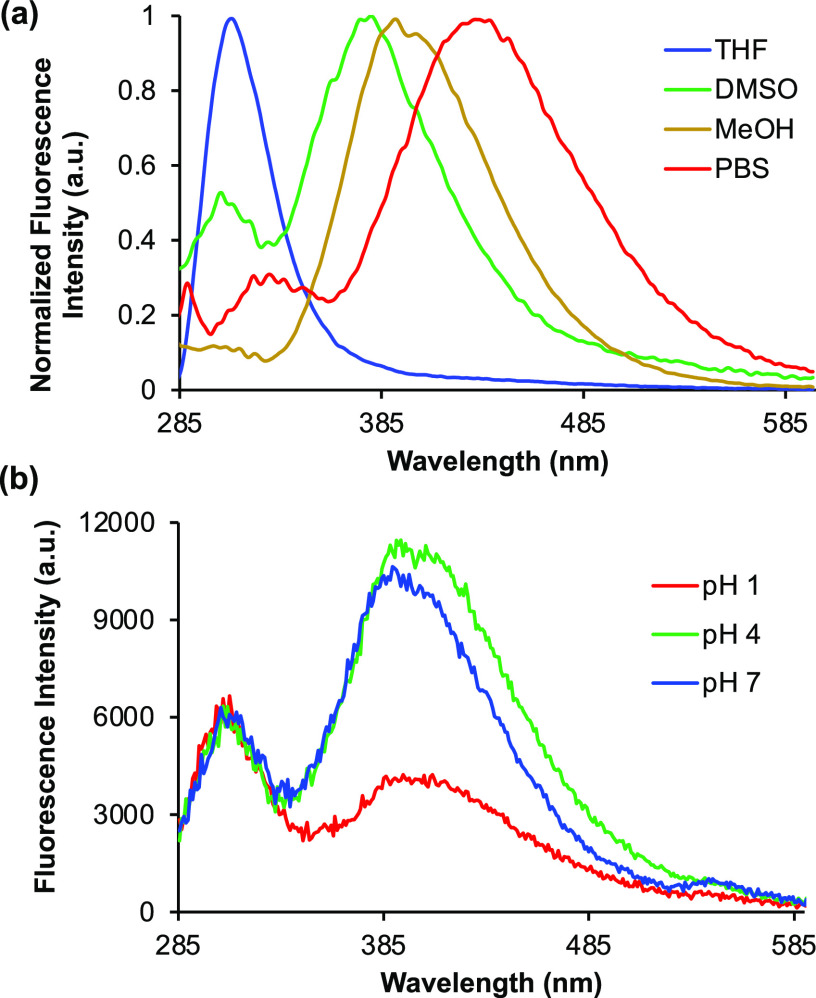
(a) Emission spectra of **10e** in various solvents. (b) Emission spectra of **10e** at pH 1, 4, and 7. All spectra were recorded by using a concentration of 5 μM.

**Scheme 3:**
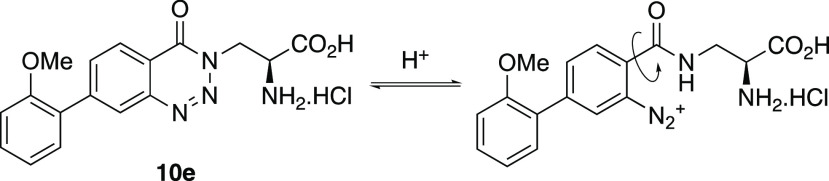
Reversible Acidic Triazinone Ring-Opening of **10e**

A proof-of-concept experiment was then conducted to assess the suitability of α-amino acid **10e** for incorporation into peptides via standard Fmoc-based solid phase peptide synthesis (SPPS) methods. The arginine-rich TAT(47–57) sequence was chosen due to the use of this peptide to deliver various conjugating groups including fluorophores into cells through transport across plasma membranes.^22^ In addition to assessing the suitability of **10e** for SPPS, we also wanted to demonstrate that a fluorescent TAT peptide could be generated using an amino acid based chromophore, rather than previously reported rhodamine- and fluorescein-derived systems.^22a,22b^ The TAT(47–57) sequence was synthesized using a Rink Amide ChemMatrix resin and an Fmoc/*tert*-butyl protecting strategy (Scheme 4). Following coupling of Fmoc-Arg(Pbf)-OH onto the polymer support using *N*,*N*′-diisopropylcarbodiimide (DIC)/OxymaPure activation, subsequent rounds of morpholine-mediated *N*-deprotection and coupling with successive amino acids gave the TAT(47–57) undecapeptide. A Fmoc-protected version of α-amino acid **10e**, compound **11** was then coupled with the polymer-supported undecapeptide.^23^ Following a final Fmoc-deprotection step, the N-terminus was capped with an acetyl group and a TFA cleavage cocktail was used to remove the side chain protecting groups and release the dodecapeptide from the polymer support. Purification by reversed-phase HPLC allowed isolation of dodecapeptide **12** in 3% overall yield and in >95% purity. Successful characterization of **12** by high resolution electrospray ionization mass spectrometry confirmed the compatibility of benzotriazinone-derived α-amino acids such as **10e** with SPPS methods.^23^ It should be noted that despite the acidic conditions of the deprotection and cleavage step, the emission spectrum of peptide **12** showed the same relative intensity of the LE and TICT bands as for amino acid **10e** at pH 4–7 (Figure 3b), further confirming successful intact incorporation of the benzotriazinone motif. In addition, both the absorption and emission spectra for peptide **12** showed good correlation with the spectra for amino acid **10e** (see the Supporting Information).

**Scheme 4:**
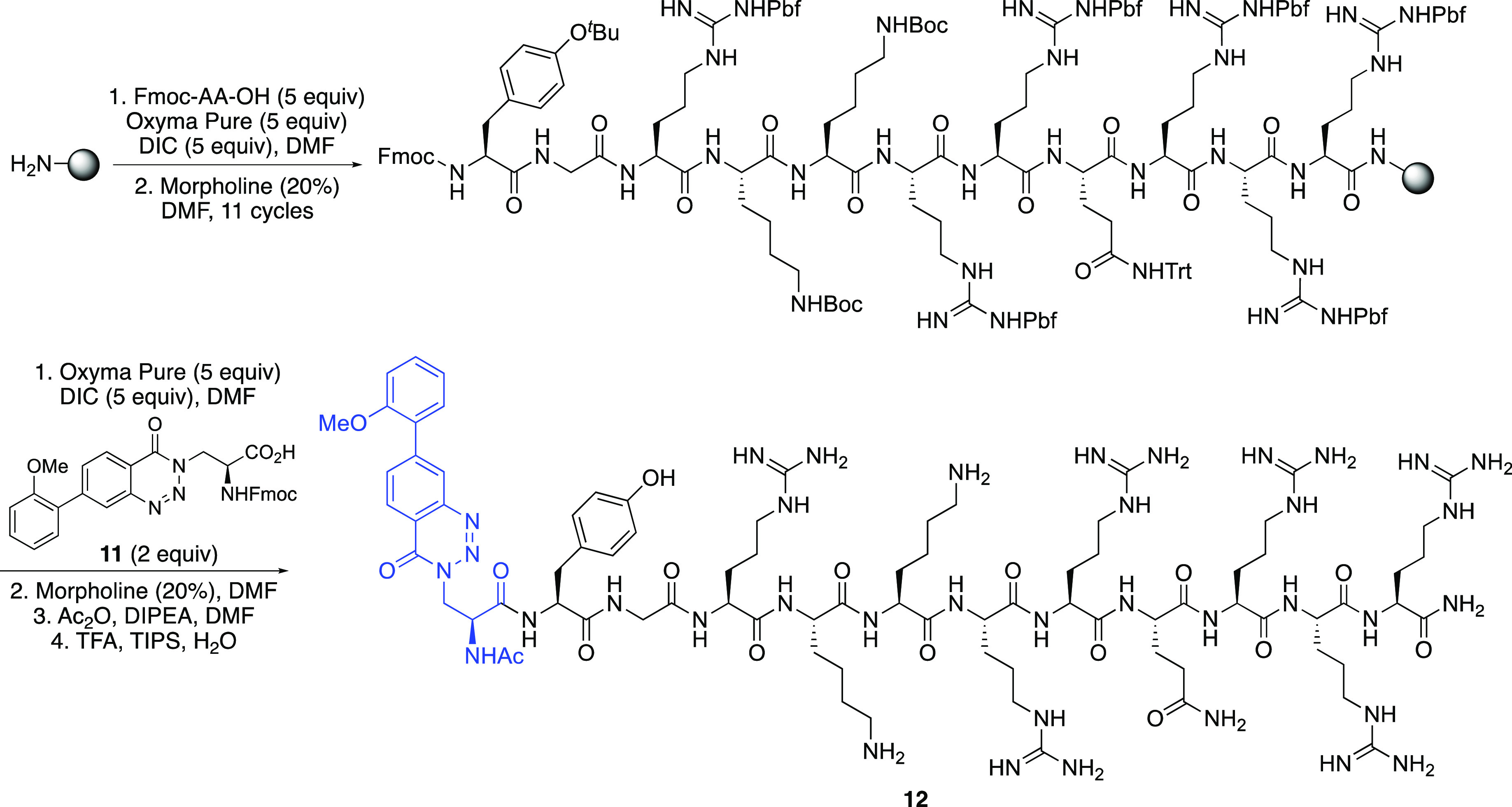
SPPS Synthesis of Dodecapeptide **12**

In summary, a one-pot diazotization and cyclization, followed by Suzuki–Miyaura cross-coupling reactions, have been used as the key steps for the synthesis of benzotriazinone α-amino acids. These biaryl compounds were found to display dual emission fluorescence based on LE and TICT excited states, with the intensity of each band being found to correlate with the electronics and positioning of substituents. This led to the design of the twisted 2-MeO-phenyl analogue **10e** which displayed bright TICT emission. In addition, amino acid **10e** displayed solvatochromism and pH sensitivity under strongly acidic conditions and was found to be compatible with SPPS. Current work is focused on exploiting potential applications of these benzotriazinone-derived α-amino acids. In addition to imaging applications of amino acid **10e**, future work will also investigate whether the strong dual emission properties of compounds such as the 4-methoxyphenyl analogue **10d** can be used to probe the local environment of peptides and proteins.

## Data Availability

The data underlying this study are available in the published article and its Supporting Information.

## Supporting Information Available

Experimental procedures, characterization data, photophysical data, NMR spectra of all compounds (PDF)

## References

[ref1] aChinen A. B.; Guan C. M.; Ferrer J. R.; Barnaby S. N.; Merkel T. J.; Mirkin C. A. Nanoparticle Probes for the Detection of Cancer Biomarkers, Cells and Tissues by Fluorescence. Chem. Rev. 2015, 115, 10530–10574. 10.1021/acs.chemrev.5b00321. 26313138 PMC5457709

[ref2] aSinkeldam R. W.; Greco N. J.; Tor Y. Fluorescent Analogs of Biomolecular Building Blocks: Design, Properties and Applications. Chem. Rev. 2010, 110, 2579–2619. 10.1021/cr900301e. 20205430 PMC2868948

[ref3] aKrueger A. T.; Imperiali B. Fluorescent Amino Acids: Modular Building Blocks for the Assembly of New Tools for Chemical Biology. ChemBioChem. 2013, 14, 788–799. 10.1002/cbic.201300079. 23609944

[ref4] Mendive-Tapia L.; Zhao C.; Akram A. R.; Preciado S.; Albericio F.; Lee M.; Serrels A.; Kielland N.; Read N. D.; Lavilla R.; Vendrell M. Spacer-Free BODIPY Fluorogens in Antimicrobial Peptides for Direct Imaging of Fungal Infection in Human Tissue. Nat. Commun. 2016, 7, 10940–10948. 10.1038/ncomms10940. 26956772 PMC4786873

[ref5] Vázquez M. E.; Rothman D. M.; Imperiali B. A New Environment-Sensitive Fluorescent Amino Acid for Fmoc-Based Solid Phase Peptide Synthesis. Chem. Commun. 2004, 2, 1965–1966. 10.1039/B408001G. 15254619

[ref6] de Moliner F.; Konieczna Z.; Mendive-Tapia L.; Saleeb R. S.; Morris K.; Gonzalez-Vera J. A.; Kaizuka T.; Grant S. G. N.; Horrocks M. H.; Vendrell M. Small Fluorogenic Amino Acids for Peptide-Guided Background-Free Imaging. Angew. Chem., Int. Ed. 2023, 62, e202216231 10.1002/anie.202216231. PMC10108274 36412996

[ref7] Behera S. K.; Park S. Y.; Gierschner J. Dual Emission: Classes, Mechanisms and Conditions. Angew. Chem., Int. Ed. 2021, 60, 22624–22638. 10.1002/anie.202009789. 32783293

[ref8] aKukhta N. A.; Bryce M. R. Dual Emission in Purely Organic Materials for Optoelectronic Applications. Mater. Horiz. 2021, 8, 33–55. 10.1039/D0MH01316A. 34821289

[ref9] Yokoo H.; Kagechika H.; Ohsaki A.; Hirano T. A Polarity-Sensitive Fluorescent Amino Acid and Its Incorporation into Peptides for the Ratiometric Detection of Biomolecular Interactions. ChemPlusChem. 2019, 84, 1716–1719. 10.1002/cplu.201900489. 31943883

[ref10] aStrizhak A. V.; Postupalenko V. Y.; Shvadchak V. V.; Morellet N.; Guittet E.; Pivovarenko V. G.; Klymchenko A. S.; Mély Y. Two-Color Fluorescent L-Amino Acid Mimic of Tryptophan for Probing Peptide-Nucleic Acid Complexes. Bioconjugate Chem. 2012, 23, 2434–2443. 10.1021/bc300464u. 23153224

[ref11] aHarkiss A. H.; Bell J. D.; Knuhtsen A.; Jamieson A. G.; Sutherland A. Synthesis and Fluorescent Properties of β-Pyridyl α-Amino Acids. J. Org. Chem. 2019, 84, 2879–2890. 10.1021/acs.joc.9b00036. 30726078

[ref12] Bell J. D.; Morgan T. E. F.; Buijs N.; Harkiss A. H.; Wellaway C. R.; Sutherland A. Synthesis and Photophysical Properties of Benzotriazole-Derived Unnatural α-Amino Acids. J. Org. Chem. 2019, 84, 10436–10448. 10.1021/acs.joc.9b01685. 31340638

[ref13] aDebia N. P.; Rodríguez J. J. P.; da Silveira C. H.; Chaves O. A.; Iglesias B. A.; Rodembusch F. S.; Lüdtke D. S. Synthesis and Photophysics of Benzazole Based Triazoles with Amino Acid-derived Pendant Units. Multiparametric Optical Sensors for BSA and CT-DNA in Solution. J. Mol. Liq. 2020, 309, 113092 10.1016/j.molliq.2020.113092.

[ref14] Stojkovic M. R.; Piotrowski P.; Schmuck C.; Piantanida I. A Short, Rigid Linker Between Pyrene and Guanidiniocarbonyl-Pyrrole Induced a New Set of Spectroscopic Responses to the ds-DNA Secondary Structure. Org. Biomol. Chem. 2015, 13, 1629–1633. 10.1039/C4OB02169J. 25502619

[ref15] McGrory R.; Faggyas R. J.; Sutherland A. One-pot Synthesis of *N*-Substituted Benzannulated Triazoles via Stable Arene Diazonium Salts. Org. Biomol. Chem. 2021, 19, 6127–6140. 10.1039/D1OB00968K. 34179913

[ref16] Filimonov V. D.; Trusova M.; Postnikov P.; Krasnokutskaya E. A.; Lee Y. M.; Hwang H. Y.; Kim H.; Chi K.-W. Unusually Stable, Versatile and Pure Arenediazonium Tosylates: Their Preparation, Structures and Synthetic Applicability. Org. Lett. 2008, 10, 3961–3964. 10.1021/ol8013528. 18722457

[ref17] Miyaura N.; Yamada K.; Suzuki A. A New Stereospecific Cross-Coupling by the Palladium-Catalyzed Reaction of 1-Alkenylboranes with 1-Alkenyl or 1-Alkynyl Halides. Tetrahedron Lett. 1979, 20, 3437–3440. 10.1016/S0040-4039(01)95429-2.

[ref18] Kinzel T.; Zhang Y.; Buchwald S. L. A New Palladium Precatalyst Allows for the Fast Suzuki-Miyaura Coupling Reactions of Unstable Polyfluorophenyl and 2-Heteroaryl Boronic Acids. J. Am. Chem. Soc. 2010, 132, 14073–14075. 10.1021/ja1073799. 20858009 PMC2953245

[ref19] Excitation spectra at the wavelength of the two emission maxima for each amino acid are provided in the Supporting Information (SI).

[ref20] aLippert E. Dipolmoment und Elektronenstruktur von Angeregten Molekülen. Z. Naturforsch. 1955, 10a, 541–545. 10.1515/zna-1955-0707.

[ref21] Under basic conditions, no emission was observed for amino acid **10e**.

[ref22] aSrinivasan D.; Muthukrishnan N.; Johnson G. A.; Erazo-Oliveras A.; Lim J.; Simanek E. E.; Pellois J.-P. Conjugation to the Cell-Penetrating Peptide TAT Potentiates the Photodynamic Effect of Carboxytetramethylrhodamine. PLoS One 2011, 6, e17732 10.1371/journal.pone.0017732. 21423812 PMC3056768

[ref23] See SI for synthesis of compound **11** and characterization data for dodecapeptide **12**.

