# Supplementary material for: Rotamer-Controlled Dual Emissive α-Amino Acids
Source: Org Lett. 2023 Jul 28;25(31):5844–9. doi: 10.1021/acs.orglett.3c02112 (PMC10425982; doi:10.1021/acs.orglett.3c02112)

Supporting Information for:

Rotamer-Controlled Dual-Emissive α-Amino Acids

Rochelle McGrory,[†] Danielle C. Morgan,[‡] Andrew G. Jamieson[‡] and Andrew Sutherland[†]*

[†]School of Chemistry, The Joseph Black Building, University of Glasgow, Glasgow G12 8QQ, United Kingdom. [‡]School of Chemistry, Advanced Research Centre, University of Glasgow, Glasgow G11 6EW, United Kingdom

Table of Contents

1. General Experimental	S2–S3
2. Experimental Procedures and Spectroscopic Data for all Compounds	S3–S17
3. Photophysical Data for α-Amino Acids **10a**–**e** and peptide **12**	S18–S27
4. References	S28
5. ^{1}H and ^{13}C NMR Spectra for all Novel Compounds	S29–S65

1. General Experimental

The synthesis of compound **4** has been previously described in the literature.[1] All reagents and starting materials were obtained from commercial sources and used as received. Reactions were performed open to air unless otherwise mentioned. All reactions performed at elevated temperatures were heated using an oil bath. Brine refers to a saturated aqueous solution of sodium chloride. Flash column chromatography was performed using silica gel 60 (40–63 μm). Aluminium-backed plates pre-coated with silica gel $60F_{254}$ were used for thin layer chromatography and were visualized with a UV lamp or by staining with potassium permanganate, vanillin or ninhydrin. ^{1}H NMR spectra were recorded on a NMR spectrometer at either 400 or 500 MHz and data are reported as follows: chemical shift in ppm relative to the solvent as internal standard ($CHCl_3$, δ 7.26 ppm; CH_3OH, δ 3.31 ppm; DMSO, δ 2.50), multiplicity (s = singlet, d = doublet, t = triplet, q = quartet, m = multiplet or overlap of non-equivalent resonances, integration). ^{13}C NMR spectra were recorded on a NMR spectrometer at either 101 or 126 MHz and data are reported as follows: chemical shift in ppm relative to tetramethylsilane or the solvent as internal standard ($CDCl_3$, δ 77.2 ppm; CD_3OD, δ 49.0 ppm; DMSO-d_6, δ 39.5), multiplicity with respect to hydrogen (deduced from DEPT experiments, C, CH, CH_2 or CH_3). Infrared spectra were recorded on a FTIR spectrometer; wavenumbers are indicated in cm^{-1}. Mass spectra were recorded using electrospray techniques. HRMS spectra were recorded using quadrupole time of flight (Q-TOF) mass spectrometers. Melting points are uncorrected. Optical rotations were determined as solutions irradiating with the sodium D line (λ = 589 nm) using a polarimeter. $[\alpha]_D$ values are given in units 10^{-1} deg cm^{-1} g^{-1}. UV-Vis and fluorescence spectra were recorded on a Horiba Duetta spectrometer. Absorbance spectra were recorded with an integration time of 0.05 s and a band pass of 5 nm. Fluorescence spectra were recorded with excitation and emission band pass of 5 nm, an integration time of 2 s, and with detector accumulations set to 1. Quantum yield data were measured using anthracene and L-tryptophan as standard references.

Quantum yields were determined using a comparative method against two standards.[2] Anthracene (Φ = 0.27, in ethanol) and L-tryptophan (Φ = 0.14 in water) were used as standard references. The integrated fluorescence intensity of each compound was determined from the emission spectra given. Measurements were performed at a minimum of four different concentrations. Concentrations were chosen to ensure the absorption value was below 0.1 to avoid re-absorption effects. Integrated fluorescence intensity was plotted as a function of the measured absorbance and a linear fit was calculated. The resultant gradient was then used to calculate the quantum yield, using the following equation:

$$\phi_x = \phi_{ST}\left(\frac{Grad_{ST}}{Grad_x}\right)\left(\frac{\eta_x^2}{\eta_{ST}^2}\right)$$

Subscript *ST* signifies the quantities associated with the quantum yield standard. Subscript *X* signifies the quantities associated with the novel compound. $Grad_x$ is the determined gradient associated with the novel compound. $Grad_{ST}$ is the determined gradient associated with quantum yield standard. η is the refractive index of the solvent used in the fluorescence measurements. η = 1.333 for water, 1.361 for ethanol and 1.331 for methanol.

2. Experimental Procedures and Spectroscopic Data for all Compounds

Methyl (2*S*)-2-(*tert*-butoxycarbonylamino)-3-(2'-nitro-4'-bromobenzoylamino)propanoate (6).

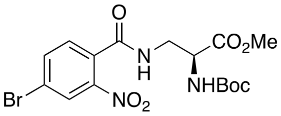

To a stirred solution of 2-nitro-4-bromobenzoic acid (0.447 g, 1.82 mmol) in toluene (9 mL) was added thionyl chloride (0.900 mL, 12.1 mmol). The reaction mixture was heated under reflux for 4 h. The reaction mixture was cooled to room temperature, concentrated *in vacuo* and azeotroped with chloroform (5 × 50 mL). A solution of 2-nitro-4-bromobenzoyl chloride (**5**) in ethyl acetate (4.5 mL) was added dropwise at 0 °C to a stirred solution of methyl (2*S*)-2-(*tert*-butoxycarbonylamino)-3-aminopropanoate (**4**) (0.264 g, 1.21 mmol) and triethylamine (0.350 mL, 2.42 mmol) in ethyl acetate (4.5 mL). The reaction mixture was heated to 90 °C and stirred for 18 h. The reaction mixture was cooled to room temperature and concentrated *in vacuo*. Purification by flash column chromatography, eluting with 40% ethyl acetate in hexane gave methyl (2*S*)-2-(*tert*-butoxycarbonylamino)-3-(2'-nitro-4'-bromobenzoylamino)propanoate (**6**) (0.306 g, 57%) as a white solid. Mp 165–170 °C; IR (neat) 3302, 2978, 1744, 1698, 1651, 1536, 1348, 1160, 732 cm^{-1}; $[\alpha]_D^{18}$ +31.8 (*c* 0.1, $CHCl_3$); ^1H NMR (500 MHz, $CDCl_3$) δ 8.17 (s, 1H), 7.77 (d, 1H, *J* = 8.1 Hz), 7.40 (d, 1H, *J* = 8.1 Hz), 6.72 (br s, 1H), 5.58 (d, 1H, *J* = 7.0 Hz), 4.53–4.43 (m, 1H), 3.93–3.74 (m, 5H), 1.42 (s, 9H); $^{13}C\{^1H\}$ NMR (126 MHz, $CDCl_3$) δ 171.0 (C), 166.1 (C), 156.1 (C), 147.1 (C), 136.8 (CH), 131.3 (C), 130.2 (CH), 127.8 (CH), 124.2 (C), 80.8 (C), 53.6 (CH), 53.1 (CH_3), 42.8 (CH_2), 28.4 (3 × CH_3); MS (ESI) *m/z* 468 (M + Na^+, 100); HRMS (ESI) *m/z*: $[M + Na]^+$ Calcd for $C_{16}H_{20}{}^{79}BrN_3O_7Na$ 468.0377; Found 468.0377.

Methyl (2*S*)-2-(*tert*-butoxycarbonylamino)-3-(2'-amino-4'-bromobenzoylamino)propanoate (7).

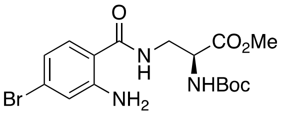

To a stirred solution of methyl (2*S*)-2-(*tert*-butoxycarbonylamino)-3-(2′-nitro-4'-bromobenzoylamino)propanoate (**6**) (0.306 g, 0.686 mmol) in methanol (2 mL) was added zinc powder

(0.448 g, 6.86 mmol) and acetic acid (0.392 mL, 6.86 mmol). The reaction mixture was stirred at room temperature for 5 h. The reaction mixture was filtered through a pad of Celite®, washed with methanol (60 mL) and concentrated *in vacuo.* The reaction mixture was diluted in ethyl acetate (25 mL) and was washed with water (5 × 25 mL). The organic layer was dried ($MgSO_4$), filtered and concentrated *in vacuo.* Purification by flash column chromatography, eluting with 35% ethyl acetate in hexane gave methyl (2*S*)-2-(*tert*-butoxycarbonylamino)-3-(2'-amino-4'-bromobenzoylamino)propanoate (**7**) (0.259 g, 91%) as a white solid. Mp 115–120 °C; IR (neat) 3347, 2977, 1743, 1698, 1515, 1367, 1248, 1158, 909, 730 cm^{-1}; $[\alpha]_D^{18}$ +30.9 (*c* 0.1, $CHCl_3$); ^{1}H NMR (500 MHz, $CDCl_3$) δ 7.19 (d, 1H, *J* = 8.4 Hz), 6.88 (br s, 1H), 6.83 (s, 1H), 6.74 (br d, 1H, *J* = 8.4 Hz), 5.58 (d, 1H, *J* = 7.1 Hz), 4.54–4.47 (m, 1H), 3.83–3.65 (m, 5H), 1.43 (s, 9H); ^{13}C{^{1}H} NMR (126 MHz, $CDCl_3$) δ 171.1 (C), 169.2 (C), 156.3 (C), 149.9 (C), 128.9 (CH), 126.9 (C), 119.8 (2 × CH), 114.2 (C), 80.8 (C), 53.6 (CH), 53.1 (CH_3), 43.0 (CH_2), 28.4 (3 × CH_3); MS (ESI) *m/z* 438 (M + Na$^+$, 100); HRMS (ESI) *m/z*: [M + Na]$^+$ Calcd for $C_{16}H_{22}{}^{79}BrN_3O_5Na$ 438.0635; Found 438.0632.

Methyl (2*S*)-2-(*tert*-butoxycarbonylamino)-3-[7'-bromo-1',2',3'-benzotriazin-4'(3*H*)-one]propanoate (8).

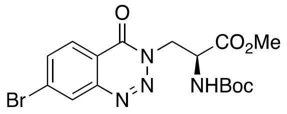

To a stirred solution of methyl (2*S*)-2-(*tert*-butoxycarbonylamino)-3-(2′-amino-4'-bromo-benzoylamino)propanoate (**7**) (1.23 g, 2.97 mmol) in methanol (20 mL) at 0 °C was added polymer-supported nitrite (2.53 g, containing 8.85 mmol of NO_2) and *p*-toluenesulfonic acid monohydrate (1.68 g, 8.85 mmol). The reaction mixture was stirred at 0 °C for 1.5 h. The reaction mixture was filtered and the resulting resin was washed with methanol (60 mL). The reaction mixture was concentrated *in vacuo*. Purification by flash column chromatography, eluting with 30% ethyl acetate in hexane gave methyl (2*S*)-2-(*tert*-butoxycarbonylamino)-3-[7'-bromo-1',2',3'-benzotriazin-4'(3*H*)-one]propanoate (**8**) (1.16 g, 92%) as a colourless oil. IR (neat) 3369, 2974, 1747, 1693, 1597, 1367, 1162, 758 cm^{-1}; $[\alpha]_D^{23}$ –1.7 (*c* 3.0, $CHCl_3$); ^{1}H NMR (500 MHz, $CDCl_3$) δ 8.28 (s, 1H), 8.20 (d, 1H, *J* = 8.4 Hz), 7.89 (d, 1H, *J* = 8.4 Hz), 5.40 (d, 1H, *J* = 7.6 Hz), 4.94 (dd, 1H, *J* = 13.2, 3.9 Hz), 4.91–4.84 (m, 1H), 4.67 (dd, 1H, *J* = 13.2, 7.4 Hz), 3.80 (s, 3H), 1.31 (s, 9H); ^{13}C{^{1}H} NMR (126 MHz, $CDCl_3$) δ 170.3 (C), 155.6 (C), 155.2 (C), 144.8 (C), 136.0 (CH), 131.0 (CH), 129.9 (C), 127.0 (CH), 118.5 (C), 80.5 (C), 53.1 (CH_3), 52.6 (CH), 51.1 (CH_2), 28.2 (3 × CH_3); MS (ESI) *m/z* 449 (M + Na$^+$, 100); HRMS (ESI) *m/z*: [M + Na]$^+$ Calcd for $C_{16}H_{19}{}^{79}BrN_4O_5Na$ 449.0431; Found 449.0433.

Methyl (2*S*)-2-(*tert*-butoxycarbonylamino)-3-[7'-phenyl-1',2',3'-benzotriazin-4'(3*H*)-one]propanoate (9a).

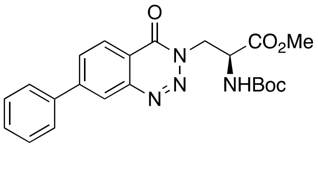

A solution of methyl (2*S*)-2-(*tert*-butoxycarbonylamino)-3-[7'-bromo-1',2',3'-benzotriazin-4'(3*H*)-one]propanoate (**8**) (0.082 g, 0.19 mmol), phenylboronic acid (0.035 g, 0.29 mmol) and potassium phosphate (0.081 g, 0.38 mmol) in tetrahydrofuran/water (1:2, 3 mL) was degassed under argon for 0.2 h. To this was added XPhos Pd G2 (0.0030 g, 0.0038 mmol, 2 mol%). The reaction mixture was stirred at 40 °C for 1 h. The reaction mixture was cooled to room temperature and concentrated *in vacuo.* The reaction mixture was diluted in water (15 mL) and extracted with ethyl acetate (3 × 15 mL). The organic layer was dried ($MgSO_4$), filtered and concentrated *in vacuo.* Purification by flash column chromatography, eluting with 2.5–5% ethyl acetate in dichloromethane gave methyl (2*S*)-2-(*tert*-butoxycarbonylamino)-3-[7'-phenyl-1',2',3'-benzotriazin-4'(3*H*)-one]propanoate (**9a**) (0.075 g, 91%) as a white solid. Mp 125–130 °C; IR (neat) 3379, 2978, 1748, 1686, 1616, 1505, 1304, 1161, 733 cm^{-1}; $[\alpha]_D^{22}$ –4.5 (*c* 0.1, $CHCl_3$); ^1H NMR (400 MHz, $CDCl_3$) δ 8.41 (d, 1H, J = 8.3 Hz), 8.33 (d, 1H, J = 1.3 Hz), 8.04 (dd, 1H, J = 8.3, 1.3 Hz), 7.75–7.69 (m, 2H), 7.58–7.51 (m, 2H), 7.48 (tt, 1H, J = 9.0, 1.5 Hz), 5.45 (d, 1H, J = 7.2 Hz), 5.00–4.87 (m, 2H), 4.74 (dd, 1H, J = 12.9, 6.7 Hz), 3.81 (s, 3H), 1.34 (s, 9H); $^{13}C\{^1H\}$ NMR (101 MHz, $CDCl_3$) δ 170.5 (C), 156.1 (C), 155.2 (C), 148.3 (C), 144.8 (C), 138.7 (C), 131.6 (CH), 129.5 (2 × CH), 129.3 (CH), 127.7 (2 × CH), 126.4 (CH), 126.0 (CH), 118.4 (C), 80.4 (C), 53.04 (CH_3), 52.98 (CH), 50.7 (CH_2), 28.3 (3 × CH_3); MS (ESI) *m/z* 425 (M + H^+, 100); HRMS (ESI) *m/z*: $[M + H]^+$ Calcd for $C_{22}H_{24}N_4O_5H$ 425.1819; Found 425.1816.

Methyl (2*S*)-2-(*tert*-butoxycarbonylamino)-3-[7'-(4''-chlorophenyl)-1',2',3'-benzotriazin-4'(3*H*)-one]propanoate (9b).

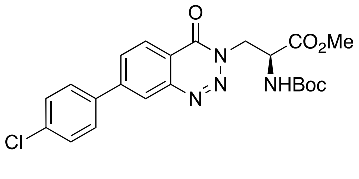

The reaction was performed as described for **9a** using methyl (2*S*)-2-(*tert*-butoxycarbonylamino)-3-[7'-bromo-1',2',3'-benzotriazin-4'(3*H*)-one]propanoate (**8**) (0.150 g, 0.351 mmol), 4-chlorophenylboronic acid (0.0820 g, 0.526 mmol), potassium phosphate (0.149 g, 0.702 mmol) and XPhos Pd G2 (0.00600 g, 0.00763 mmol, 2 mol%). Purification by flash column chromatography, eluting with 60% diethyl ether in hexane followed by 50% diethyl ether in hexane with 1% triethylamine gave methyl (2*S*)-2-

(*tert*-butoxycarbonylamino)-3-[7’-(4’’-chlorophenyl)-1’,2’,3’-benzotriazin-4’(3*H*)-one]propanoate (**9b**) (0.110 g, 68%) as a white solid. Mp 126–130 °C; IR (neat) 3356, 2978, 2361, 1748, 1686, 1616, 1505, 1366, 1308, 1161, 733 cm^{-1}; $[\alpha]_D^{24}$ –13.9 (*c* 0.1, $CHCl_3$); ^1H NMR (400 MHz, $CDCl_3$) δ 8.40 (d, 1H, *J* = 8.3 Hz), 8.29 (d, 1H, *J* = 1.4 Hz), 7.99 (dd, 1H, *J* = 8.3, 1.4 Hz), 7.67–7.61 (m, 2H), 7.54–7.49 (m, 2H), 5.44 (d, 1H, *J* = 7.3 Hz), 5.00–4.85 (m, 2H), 4.73 (dd, 1H, *J* = 12.9, 6.8 Hz), 3.81 (s, 3H), 1.33 (s, 9H); $^{13}C\{^1H\}$ NMR (101 MHz, $CDCl_3$) δ 170.5 (C), 155.9 (C), 155.2 (C), 147.0 (C), 144.7 (C), 137.1 (C), 135.7 (C), 131.3 (CH), 129.7 (2 × CH), 128.9 (2 × CH), 126.22 (CH), 126.17 (CH), 118.6 (C), 80.4 (C), 53.1 (CH_3), 52.9 (CH), 50.8 (CH_2), 28.3 (3 × CH_3); MS (ESI) *m/z* 481 (M + Na^+, 100); HRMS (ESI) *m/z*: $[M + Na]^+$ Calcd for $C_{22}H_{23}{}^{35}ClN_4O_5Na$ 481.1249; Found 481.1241.

Methyl (2*S*)-2-(*tert*-butoxycarbonylamino)-3-[7’-(3’’,4’’-methylenedioxybenzene)-1’,2’,3’-benzotriazin-4’(3*H*)-one]propanoate (9c).

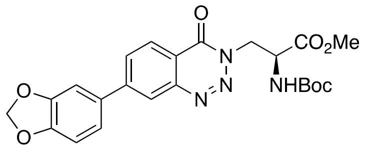

The reaction was performed as described for **9a** using methyl (2*S*)-2-(*tert*-butoxycarbonylamino)-3-[7’-bromo-1’,2’,3’-benzotriazin-4’(3*H*)-one]propanoate (**8**) (0.150 g, 0.351 mmol), 3,4-(methylenedioxy)phenylboronic acid (0.0870 g, 0.526 mmol), potassium phosphate (0.149 g, 0.702 mmol) and XPhos Pd G2 (0.00600 g, 0.00763 mmol, 2 mol%). Purification by flash column chromatography, eluting with 2.5% ethyl acetate in dichloromethane gave methyl (2*S*)-2-(*tert*-butoxycarbonylamino)-3-[7’-(3’’,4’’-methylenedioxybenzene)-1’,2’,3’-benzotriazin-4’(3*H*)-one]propanoate (**9c**) (0.126 g, 79%) as a white solid. Mp 76–80 °C; IR (neat) 3358, 2955, 2361, 2342, 1748, 1686, 1613, 1505, 1462, 1304, 1234, 1165, 1038, 733 cm^{-1}; $[\alpha]_D^{24}$ –16.9 (*c* 0.1, $CHCl_3$); ^1H NMR (400 MHz, $CDCl_3$) δ 8.36 (d, 1H, *J* = 8.3 Hz), 8.24 (d, 1H, *J* = 1.4 Hz), 7.95 (dd, 1H, *J* = 8.3, 1.4 Hz), 7.20 (dd, 1H, *J* = 8.0, 1.7 Hz), 7.18 (d, 1H, *J* = 1.7 Hz), 6.96 (d, 1H, *J* = 8.0 Hz), 6.06 (s, 2H), 5.45 (d, 1H, *J* = 7.3 Hz), 5.00–4.84 (m, 2H), 4.74 (dd, 1H, *J* = 12.8, 6.6 Hz), 3.81 (s, 3H), 1.34 (s, 9H); $^{13}C\{^1H\}$ NMR (101 MHz, $CDCl_3$) δ 170.5 (C), 156.1 (C), 155.2 (C), 148.9 (C), 148.8 (C), 147.9 (C), 144.8 (C), 132.9 (C), 131.3 (CH), 125.9 (CH), 125.8 (CH), 121.8 (CH), 118.0 (C), 109.2 (CH), 107.8 (CH), 101.8 (CH_2), 80.4 (C), 53.03 (CH_3), 53.00 (CH), 50.7 (CH_2), 28.3 (3 × CH_3); MS (ESI) *m/z* 491 (M + Na^+, 100); HRMS (ESI) *m/z*: $[M + Na]^+$ Calcd for $C_{23}H_{24}N_4O_7Na$ 491.1537; Found 491.1543.

Methyl (2*S*)-2-(*tert*-butoxycarbonylamino)-3-[7'-(4''-methoxyphenyl)-1',2',3'-benzotriazin-4'(3*H*)-one]propanoate (9d).

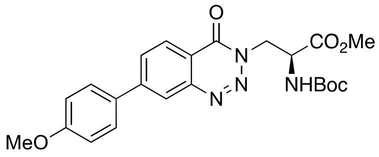

The reaction was performed as described for **9a** using methyl (2*S*)-2-(*tert*-butoxycarbonylamino)-3-[7'-bromo-1',2',3'-benzotriazin-4'(3*H*)-one]propanoate (**8**) (0.103 g, 0.241 mmol), 4-methoxyphenylboronic acid (0.0550 g, 0.361 mmol), potassium phosphate (0.102 g, 0.482 mmol) and XPhos Pd G2 (0.00400 g, 0.00508 mmol, 2 mol%). Purification by flash column chromatography, eluting with 2.5% ethyl acetate in dichloromethane gave methyl (2*S*)-2-(*tert*-butoxycarbonylamino)-3-[7'-(4''-methoxyphenyl)-1',2',3'-benzotriazin-4'(3*H*)-one]propanoate (**9d**) (0.101 g, 93%) as a colorless oil. IR (neat) 3366, 2976, 2541, 2250, 1973, 1748, 1682, 1605, 1524, 1251, 1162, 734 cm^{-1}; $[\alpha]_D^{22}$ –10.6 (*c* 0.1, $CHCl_3$); ^{1}H NMR (500 MHz, $CDCl_3$) δ 8.36 (d, 1H, *J* = 8.3 Hz), 8.28 (s, 1H), 8.00 (d, 1H, *J* = 8.3 Hz), 7.67 (d, 2H, *J* = 8.5 Hz), 7.06 (d, 2H, *J* = 8.5 Hz), 5.47 (d, 1H, *J* = 7.6 Hz), 4.98–4.85 (m, 2H), 4.74 (dd, 1H, *J* = 13.1, 6.8 Hz), 3.89 (s, 3H), 3.81 (s, 3H), 1.34 (s, 9H); ^{13}C{^{1}H} NMR (126 MHz, $CDCl_3$) δ 170.6 (C), 160.7 (C), 156.1 (C), 155.2 (C), 147.8 (C), 144.8 (C), 131.1 (CH), 131.0 (C), 128.8 (2 × CH), 125.9 (CH), 125.4 (CH), 117.7 (C), 114.9 (2 × CH), 80.4 (C), 55.6 (CH_3), 53.1 (CH_3), 53.0 (CH), 50.7 (CH_2), 28.3 (3 × CH_3); MS (ESI) *m/z* 477 (M + Na$^+$, 100); HRMS (ESI) *m/z*: [M + Na]$^+$ Calcd for $C_{23}H_{26}N_4O_6Na$ 477.1745; Found 477.1744.

Methyl (2*S*)-2-(*tert*-butoxycarbonylamino)-3-[7'-(2''-methoxyphenyl)-1',2',3'-benzotriazin-4'(3*H*)-one]propanoate (9e).

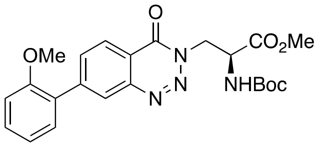

The reaction was performed as described for **9a** using methyl (2*S*)-2-(*tert*-butoxycarbonylamino)-3-[7'-bromo-1',2',3'-benzotriazin-4'(3*H*)-one]propanoate (**8**) (0.100 g, 0.234 mmol), 2-methoxyphenylboronic acid (0.0530 g, 0.351 mmol), potassium phosphate (0.0990 g, 0.468 mmol) and XPhos Pd G2 (0.00400 g, 0.00508 mmol, 2 mol%). Purification by flash column chromatography, eluting with 30% ethyl acetate in hexane gave methyl (2*S*)-2-(*tert*-butoxycarbonylamino)-3-[7'-(2''-methoxyphenyl)-1',2',3'-benzotriazin-4'(3*H*)-one]propanoate (**9e**) (0.101 g, 95%) as a white solid. Mp 190–195 °C; IR (neat) 3370, 2956, 2524, 2249, 1971, 1748, 1682, 1615, 1491, 1244, 1163, 911, 734 cm^{-1}; $[\alpha]_D^{25}$ –1.4 (*c* 0.1, $CHCl_3$); ^{1}H NMR (500 MHz, $CDCl_3$) δ 8.35 (d, 1H, *J* = 8.2 Hz), 8.31 (br s,

1H), 7.99 (dd, 1H, J = 8.2, 1.5 Hz), 7.46–7.42 (m, 1H), 7.40 (dd, 1H, J = 7.6, 1.5 Hz), 7.11 (td, 1H, J = 7.6, 1.0 Hz), 7.05 (br d, 1H, J = 8.3 Hz), 5.47 (d, 1H, J = 7.7 Hz), 4.95 (dd, 1H, J = 13.3, 4.2 Hz), 4.92–4.85 (m, 1H), 4.76 (dd, 1H, J = 13.3, 6.8 Hz), 3.85 (s, 3H), 3.81 (s, 3H), 1.35 (s, 9H); $^{13}C\{^1H\}$ NMR (126 MHz, $CDCl_3$) δ 170.6 (C), 156.6 (C), 156.2 (C), 155.2 (C), 146.1 (C), 144.3 (C), 134.4 (CH), 131.0 (CH), 130.5 (CH), 129.0 (CH), 128.2 (C), 124.8 (CH), 121.4 (CH), 118.0 (C), 111.6 (CH), 80.4 (C), 55.7 (CH_3), 53.1 (CH), 53.0 (CH_3), 50.5 (CH_2), 28.3 (3 × CH_3); MS (ESI) m/z 477 (M + Na^+, 100); HRMS (ESI) m/z: $[M + Na]^+$ Calcd for $C_{23}H_{26}N_4O_6Na$ 477.1745; Found 477.1741.

(2*S*)-2-(*tert*-Butoxycarbonylamino)-3-[7'-phenyl-1',2',3'-benzotriazin-4'(3*H*)-one]propanoic acid (S1).

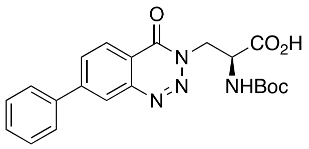

To a stirred solution of methyl (2*S*)-2-(*tert*-butoxycarbonylamino)-3-[7'-phenyl-1',2',3'-benzotriazin-4'(3*H*)-one]propanoate (**9a**) (0.072 g, 0.17 mmol) in methanol (3 mL), dioxane (1.8 mL) and water (1.8 mL) was added caesium carbonate (0.072 g, 0.22 mmol). The reaction mixture was stirred at room temperature for 18 h. The reaction mixture was concentrated *in vacuo*, diluted in water (10 mL) and acidified to pH 1 using 1 M aqueous hydrochloric acid. The reaction mixture was extracted with dichloromethane (3 × 15 mL). The organic layers were combined, dried ($MgSO_4$), filtered and concentrated *in vacuo* to give (2*S*)-2-(*tert*-butoxycarbonylamino)-3-[7'-phenyl-1',2',3'-benzotriazin-4'(3*H*)-one]propanoic acid (**S1**) (0.055 g, 79%) as a white solid. Mp 155–160 °C; IR (neat) 3341, 2978, 2932, 1686, 1616, 1512, 1304, 1161, 910, 729 cm^{-1}; $[\alpha]_D^{23}$ –5.1 (c 0.1, $CHCl_3$); ^1H NMR (400 MHz, $CDCl_3$) δ 8.39 (d, 1H, J = 8.3 Hz), 8.33 (br s, 1H), 8.03 (dd, 1H, J = 8.3, 1.6 Hz), 7.74–7.67 (m, 2H), 7.57–7.42 (m, 3H), 5.61 (d, 1H, J = 7.3 Hz), 5.03 (dd, 1H, J = 13.5, 5.0 Hz), 5.00–4.92 (m, 1H), 4.82 (dd, 1H, J = 13.5, 7.1 Hz), 1.33 (s, 9H); $^{13}C\{^1H\}$ NMR (101 MHz, $CDCl_3$) δ 172.8 (C), 156.4 (C), 155.7 (C), 148.4 (C), 144.7 (C), 138.6 (C), 131.7 (CH), 129.4 (2 × CH), 129.3 (CH), 127.7 (2 × CH), 126.2 (CH), 126.0 (CH), 118.2 (C), 80.8 (C), 52.9 (CH), 50.7 (CH_2), 28.3 (3 × CH_3); MS (ESI) m/z 433 (M + Na^+, 100); HRMS (ESI) m/z: $[M + Na]^+$ Calcd for $C_{21}H_{22}N_4O_5Na$ 433.1482; Found 433.1473.

(2*S*)-2-(*tert*-Butoxycarbonylamino)-3-[7’-(4’’-chlorophenyl)-1’,2’,3’-benzotriazin-4’(3*H*)-one]propanoic acid (S2).

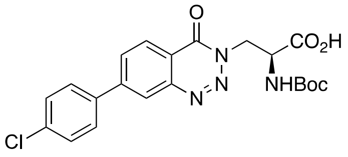

The reaction was performed as described above using methyl (2*S*)-2-(*tert*-butoxycarbonylamino)-3-[7’-(4’’-chlorophenyl)-1’,2’,3’-benzotriazin-4’(3*H*)-one]propanoate (**9b**) (0.073 g, 0.16 mmol) and caesium carbonate (0.067 g, 0.21 mmol). This gave (2*S*)-2-(*tert*-butoxycarbonylamino)-3-[7’-(4’’-chlorophenyl)-1’,2’,3’-benzotriazin-4’(3*H*)-one]propanoic acid (**S2**) (0.064 g, 90%) as a white solid. Mp 195–200 °C; IR (neat) 3345, 2978, 2928, 1740, 1686, 1616, 1520, 1308, 1161, 733 cm^{-1}; $[\alpha]_D^{21}$ –46.0 (*c* 0.1, MeOH); ^1H NMR (400 MHz, CD_3OD) δ 8.34 (d, 1H, *J* = 8.3 Hz), 8.31 (d, 1H, *J* = 1.3 Hz), 8.12 (dd, 1H, *J* = 8.3, 1.3 Hz), 7.77 (br d, 2H, *J* = 8.6 Hz), 7.55–7.50 (m, 2H), 5.00 (dd, 1H, *J* = 13.3, 4.3 Hz), 4.80 (dd, 1H, *J* = 9.5, 4.3 Hz), 4.63 (dd, 1H, *J* = 13.3, 9.5 Hz), 1.25 (s, 9H); $^{13}C\{^1H\}$ NMR (101 MHz, CD_3OD) δ 172.9 (C), 157.6 (C), 157.2 (C), 148.0 (C), 146.0 (C), 138.3 (C), 136.4 (C), 132.3 (CH), 130.5 (2 × CH), 130.1 (2 × CH), 126.8 (CH), 126.7 (CH), 119.7 (C), 80.7 (C), 53.3 (CH), 52.2 (CH_2), 28.5 (3 × CH_3); MS (ESI) *m/z* 467 (M + Na^+, 100); HRMS (ESI) *m/z*: $[M + Na]^+$ Calcd for $C_{21}H_{21}{}^{35}ClN_4O_5Na$ 467.1093; Found 467.1099.

(2*S*)-2-(*tert*-Butoxycarbonylamino)-3-[7’-(3’’,4’’-methylenedioxybenzene)-1’,2’,3’-benzotriazin-4’(3*H*)-one]propanoic acid (S3).

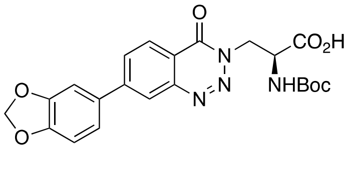

The reaction was performed as described above using methyl (2*S*)-2-(*tert*-butoxycarbonylamino)-3-[7’-(3’’,4’’-methylenedioxybenzene)-1’,2’,3’-benzotriazin-4’(3*H*)-one]propanoate (**9c**) (0.170 g, 0.363 mmol) and caesium carbonate (0.154 g, 0.472 mmol). This gave (2*S*)-2-(*tert*-butoxycarbonylamino)-3-[7’-(3’’,4’’-methylenedioxybenzene)-1’,2’,3’-benzotriazin-4’(3*H*)-one]propanoic acid (**S3**) (0.121 g, 73%) as a white solid. Mp 229–234 °C; IR (neat) 3422, 2924, 2361, 1686, 1613, 1238, 1161, 1034 cm^{-1}; $[\alpha]_D^{21}$ –43.8 (*c* 0.1, MeOH); ^1H NMR (400 MHz, DMSO-d_6) δ 8.39 (d, 1H, *J* = 1.7 Hz), 8.25 (d, 1H, *J* = 8.4 Hz), 8.19 (dd, 1H, *J* = 8.4, 1.7 Hz), 7.53 (d, 1H, *J* = 1.8 Hz), 7.42 (dd, 1H, *J* = 8.1, 1.8 Hz), 7.28 (d, 1H, *J* = 7.9 Hz), 7.08 (d, 1H, *J* = 8.1 Hz), 6.12 (s, 2H), 4.86 (dd, 1H, *J* = 11.1, 2.6 Hz), 4.57–4.44 (m, 2H), 1.22 (s, 9H); $^{13}C\{^1H\}$ NMR (101 MHz, DMSO-d_6) δ 171.2 (C), 155.3 (C), 154.9 (C), 148.4 (C), 148.3 (C), 146.4 (C), 144.3 (C), 131.7 (C), 131.0 (CH), 125.2 (CH), 124.6 (CH), 121.7 (CH), 117.5

(C), 109.0 (CH), 107.7 (CH), 101.6 (CH_2), 78.4 (C), 51.4 (CH), 50.3 (CH_2), 28.0 (3 × CH_3); MS (ESI) *m/z* 477 (M + Na^+, 100); HRMS (ESI) *m/z*: $[M + Na]^+$ Calcd for $C_{22}H_{22}N_4O_7Na$ 477.1381; Found 477.1380.

(2*S*)-2-(*tert*-Butoxycarbonylamino)-3-[7'-(4''-methoxyphenyl)-1',2',3'-benzotriazin-4'(3*H*)-one]propanoic acid (S4).

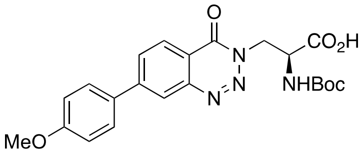

The reaction was performed as described above using methyl (2*S*)-2-(*tert*-butoxycarbonylamino)-3-[7'-(4''-methoxyphenyl)-1',2',3'-benzotriazin-4'(3*H*)-one]propanoate (**9d**) (0.10 g, 0.22 mmol) and caesium carbonate (0.094 g, 0.29 mmol). This gave (2*S*)-2-(*tert*-butoxycarbonylamino)-3-[7'-(4''-methoxyphenyl)-1',2',3'-benzotriazin-4'(3*H*)-one]propanoic acid (**S4**) (0.093 g, 95%) as a white solid. Mp 210–215 °C (decomposition); IR (neat) 3372, 2248, 1974, 1733, 1705, 1651, 1521, 1159, 908, 728 cm^{-1}; $[\alpha]_D^{25}$ +1.7 (*c* 0.1, $CHCl_3$); ^1H NMR (400 MHz, $CDCl_3$) δ 8.36 (d, 1H, J = 8.3 Hz), 8.29 (d, 1H, J = 1.6 Hz), 8.00 (dd, 1H, J = 8.3, 1.6 Hz), 7.67 (d, 2H, J = 8.7 Hz), 7.05 (d, 2H, J = 8.7 Hz), 5.60 (d, 1H, J = 7.4 Hz), 5.01 (dd, 1H, J = 13.2, 4.1 Hz), 4.96–4.89 (m, 1H), 4.83 (dd, 1H, J = 13.2, 7.4 Hz), 3.89 (s, 3H), 1.35 (s, 9H); $^{13}C\{^1H\}$ NMR (101 MHz, $CDCl_3$) δ 172.5 (C), 160.8 (C), 156.5 (C), 155.7 (C), 148.0 (C), 144.8 (C), 131.2 (CH), 130.9 (C), 128.8 (2 × CH), 125.9 (CH), 125.4 (CH), 117.6 (C), 114.9 (2 × CH), 80.8 (C), 55.6 (CH_3), 52.9 (CH), 50.6 (CH_2), 28.3 (3 × CH_3); MS (ESI) *m/z* 463 (M + Na^+, 100); HRMS (ESI) *m/z*: $[M + Na]^+$ Calcd for $C_{22}H_{24}N_4O_6Na$ 463.1588; Found 463.1591.

(2*S*)-2-(*tert*-Butoxycarbonylamino)-3-[7'-(2''-methoxyphenyl)-1',2',3'-benzotriazin-4'(3*H*)-one]propanoic acid (S5).

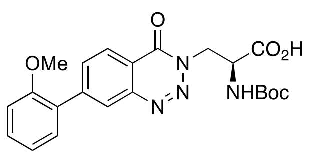

The reaction was performed as described above using methyl (2*S*)-2-(*tert*-butoxycarbonylamino)-3-[7'-(2''-methoxyphenyl)-1',2',3'-benzotriazin-4'(3*H*)-one]propanoate (**9e**) (0.10 g, 0.22 mmol) and caesium carbonate (0.093 g, 0.29 mmol). This gave (2*S*)-2-(*tert*-butoxycarbonylamino)-3-[7'-(2''-methoxyphenyl)-1',2',3'-benzotriazin-4'(3*H*)-one]propanoic acid (**S5**) (0.092 g, 95%) as an off-white solid. Mp 150–155 °C; IR (neat) 2966, 2247, 1987, 1682, 1614, 1243, 1160, 909, 731 cm^{-1}; $[\alpha]_D^{23}$ –10.6 (*c* 0.1, $CHCl_3$); ^1H NMR (400 MHz, $CDCl_3$) δ 8.35 (d, 1H, J = 8.2 Hz), 8.32 (br s, 1H), 7.99 (dd,

1H, J = 8.2, 1.3 Hz), 7.48–7.37 (m, 2H), 7.09 (t, 1H, J = 7.5 Hz), 7.04 (d, 1H, J = 8.2 Hz), 5.60 (d, 1H, J = 7.1 Hz), 5.02 (dd, 1H, J = 13.1, 4.0 Hz), 4.98–4.89 (m, 1H), 4.84 (dd, 1H, J = 13.1, 7.0 Hz), 3.84 (s, 3H), 1.35 (s, 9H); $^{13}C\{^{1}H\}$ NMR (101 MHz, $CDCl_3$) δ 172.7 (C), 156.60 (C), 156.56 (C), 155.7 (C), 146.3 (C), 144.3 (C), 134.6 (CH), 131.0 (CH), 130.6 (CH), 129.0 (CH), 128.1 (C), 124.8 (CH), 121.4 (CH), 117.9 (C), 111.6 (CH), 80.8 (C), 55.7 (CH_3), 53.0 (CH), 50.5 (CH_2), 28.3 (3 × CH_3); MS (ESI) *m*/*z* 463 (M + Na^+, 100); HRMS (ESI) *m/z*: $[M + Na]^+$ Calcd for $C_{22}H_{24}N_4O_6Na$ 463.1588; Found 463.1586.

(2*S*)-2-Amino-3-[7'-phenyl-1',2',3'-benzotriazin-4'(3*H*)-one]propanoic acid hydrochloride (10a).

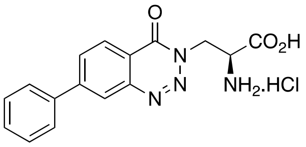

A solution of (2*S*)-2-(*tert*-butoxycarbonylamino)-3-[7'-phenyl-1',2',3'-benzotriazin-4'(3*H*)-one]propanoic acid (**S1**) (0.030 g, 0.073 mmol) in 2 M aqueous hydrochloric acid (2 mL) was heated to 50 °C and stirred for 1 h. The reaction mixture was cooled to room temperature and concentrated *in vacuo*. Purification by recrystallization from methanol and diethyl ether gave (2*S*)-2-amino-3-[7'-phenyl-1',2',3'-benzotriazin-4'(3*H*)-one]propanoic acid hydrochloride (**10a**) (0.023 g, 92%) as white solid. Mp 223–228°C; IR (neat) 3422, 2920, 2153, 1960, 1732, 1674, 1616, 1497, 1204, 1034, 760 cm^{-1}; $[\alpha]_D^{22}$ –11.9 (*c* 0.1, MeOH); ^{1}H NMR (400 MHz, DMSO-d_6) δ 8.52 (d, 1H, J = 1.7 Hz), 8.33 (d, 1H, J = 8.3 Hz), 8.29 (dd, 1H, J = 8.3, 1.7 Hz), 7.93 (d, 2H, J = 9.0 Hz), 7.58 (t, 2H, J = 9.0 Hz), 7.52 (t, 1H, J = 9.0 Hz), 4.95 (dd, 1H, J = 14.3, 5.2 Hz), 4.79 (dd, 1H, J = 14.3, 7.7 Hz), 4.44 (dd, 1H, J = 7.7, 5.2 Hz); $^{13}C\{^{1}H\}$ NMR (101 MHz, DMSO-d_6) δ 168.3 (C), 155.3 (C), 146.9 (C), 144.3 (C), 137.6 (C), 131.5 (CH), 129.3 (2 × CH), 129.2 (CH), 127.4 (2 × CH), 125.4 (2 × CH), 118.4 (C), 50.8 (CH), 48.4 (CH_2); MS (ESI) *m*/*z* 333 (M + Na^+, 100); HRMS (ESI) *m/z*: $[M + Na]^+$ Calcd for $C_{16}H_{14}N_4O_3Na$ 333.0958; Found 333.0955.

(2*S*)-2-Amino-3-[7'-(4''-chlorophenyl)-1',2',3'-benzotriazin-4'(3*H*)-one]propanoic acid hydrochloride (10b).

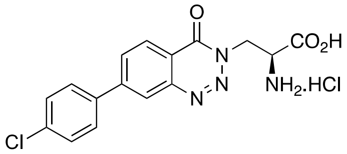

The reaction was performed as described for **10a** using (2*S*)-2-(*tert*-butoxycarbonylamino)-3-[7'-(4''-chlorophenyl)-1',2',3'-benzotriazin-4'(3*H*)-one]propanoic acid (**S2**) (0.040 g, 0.090 mmol). This gave

(2*S*)-2-amino-3-[7’-(4’’-chlorophenyl)-1’,2’,3’-benzotriazin-4’(3*H*)-one]propanoic acid hydrochloride (**10b**) (0.035 g, 95%) as a white solid. Mp 230–235 °C; IR (neat) 3457, 2928, 2361, 2168, 1975, 1682, 1593, 1512, 1343, 1312, 1099, 853 cm^{-1}; $[\alpha]_D^{21}$ +46.9 (*c* 0.1, MeOH); ^1H NMR (400 MHz, DMSO-d_6) δ 8.55 (d, 1H, J = 1.3 Hz), 8.33 (d, 1H, J = 8.0 Hz), 8.29 (dd, 1H, J = 8.0, 1.3 Hz), 8.01–7.95 (m, 2H), 7.66–7.61 (m, 2H), 4.94 (dd, 1H, J = 14.3, 5.3 Hz), 4.78 (dd, 1H, J = 14.3, 7.6 Hz), 4.45 (dd, 1H, J = 7.6, 5.3 Hz); $^{13}C\{^1H\}$ NMR (101 MHz, DMSO-d_6) δ 168.4 (C), 155.3 (C), 145.6 (C), 144.3 (C), 136.4 (C), 134.3 (C), 131.4 (CH), 129.33 (2 × CH), 129.30 (2 × CH), 125.51 (CH), 125.48 (CH), 118.6 (C), 50.9 (CH), 48.4 (CH_2); MS (ESI) *m/z* 367 (M + Na^+, 100); HRMS (ESI) *m/z*: $[M + Na]^+$ Calcd for $C_{16}H_{13}{}^{35}ClN_4O_3Na$ 367.0568; Found 367.0550.

(2*S*)-2-Amino-3-[7’-(3’’,4’’-methylenedioxybenzene)-1’,2’,3’-benzotriazin-4’(3*H*)-one]propanoic acid hydrochloride (10c).

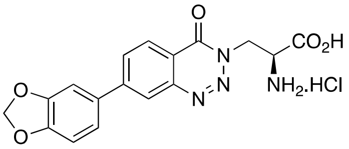

The reaction was performed as described for **10a** using (2*S*)-2-(*tert*-butoxycarbonylamino)-3-[7’-(3’’,4’’-methylenedioxybenzene)-1’,2’,3’-benzotriazin-4’(3*H*)-one]propanoic acid (**S3**) (0.025 g, 0.055 mmol). This gave (2*S*)-2-amino-3-[7’-(3’’,4’’-methylenedioxybenzene)-1’,2’,3’-benzotriazin-4’(3*H*)-one]propanoic acid hydrochloride (**10c**) (0.020 g, 95%) as a white solid. Mp 223–227 °C; IR (neat) 3140, 2909, 1767, 1678, 1609, 1501, 1034, 814 cm^{-1}; $[\alpha]_D^{21}$ +57.7 (*c* 0.1, DMSO); ^1H NMR (400 MHz, DMSO-d_6) δ 8.47 (d, 1H, J = 1.3 Hz), 8.28 (d, 1H, J = 8.4 Hz), 8.24 (dd, 1H, J = 8.4, 1.3 Hz), 7.58 (d, 1H, J = 1.9 Hz), 7.46 (dd, 1H, J = 8.1, 1.9 Hz), 7.11 (d, 1H, J = 8.1 Hz), 6.13 (s, 2H), 4.94 (dd, 1H, J = 14.4, 5.1 Hz), 4.76 (dd, 1H, J = 14.4, 7.7 Hz), 4.42 (dd, 1H, J = 7.7, 5.1 Hz); $^{13}C\{^1H\}$ NMR (101 MHz, DMSO-d_6) δ 168.4 (C), 155.3 (C), 148.41 (C), 148.38 (C), 146.6 (C), 144.3 (C), 131.6 (CH), 131.2 (C), 125.2 (CH), 124.8 (CH), 121.7 (CH), 117.9 (C), 109.0 (CH), 107.7 (CH), 101.6 (CH_2), 50.8 (CH), 48.3 (CH_2); MS (ESI) *m/z* 377 (M + Na^+, 100); HRMS (ESI) *m/z*: $[M + Na]^+$ Calcd for $C_{17}H_{14}N_4O_5Na$ 377.0856; Found 377.0850.

(2*S*)-2-Amino-3-[7’-(4’’-methoxyphenyl)-1’,2’,3’-benzotriazin-4’(3*H*)-one]propanoic acid hydrochloride (10d).

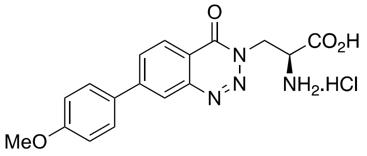

The reaction was performed as described for **10a** using (2*S*)-2-(*tert*-butoxycarbonylamino)-3-[7’-(4’’-methoxyphenyl)-1’,2’,3’-benzotriazin-4’(3*H*)-one]propanoic acid (0.044 g, 0.10 mmol) (**S4**). This gave (2*S*)-2-amino-3-[7’-(4’’-methoxyphenyl)-1’,2’,3’-benzotriazin-4’(3*H*)-one]propanoic acid hydrochloride (**10d**) (0.032 g, 84%) as a yellow solid. Mp 228–233 °C; IR (neat) 3403, 2922, 2219, 1765, 1678, 1601, 1251, 1034, 852 cm^{-1}; $[\alpha]_D^{22}$ +48.3 (*c* 0.1, MeOH); ^{1}H NMR (400 MHz, DMSO-d_6) δ 8.46 (s, 1H), 8.32–8.22 (m, 2H), 7.91 (d, 2H, *J* = 8.7 Hz), 7.12 (d, 2H, *J* = 8.7 Hz), 4.93 (dd, 1H, *J* = 14.4, 4.9 Hz), 4.76 (dd, 1H, *J* = 14.4, 7.7 Hz), 4.45–4.36 (m, 1H), 3.84 (s, 3H); ^{13}C{^{1}H} NMR (101 MHz, DMSO-d_6) δ 168.3 (C), 160.3 (C), 155.3 (C), 146.6 (C), 144.4 (C), 130.9 (CH), 129.7 (C), 128.8 (2 × CH), 125.3 (CH), 124.4 (CH), 117.7 (C), 114.8 (2 × CH), 55.4 (CH_3), 51.0 (CH), 48.5 (CH_2); MS (ESI) *m/z* 339 ([M–H]$^-$, 100); HRMS (ESI) *m/z*: [M–H]$^-$ Calcd for $C_{17}H_{15}N_4O_4$ 339.1099; Found 339.1098.

(2*S*)-2-Amino-3-[7’-(2’’-methoxyphenyl)-1’,2’,3’-benzotriazin-4’(3*H*)-one]propanoic acid hydrochloride (10e).

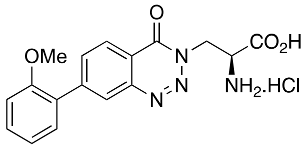

The reaction was performed as described for **10a** using (2*S*)-2-(*tert*-butoxycarbonylamino)-3-[7’-(2’’-methoxyphenyl)-1’,2’,3’-benzotriazin-4’(3*H*)-one]propanoic acid (**S5**) (0.020 g, 0.045 mmol). This gave (2*S*)-2-amino-3-[7’-(2’’-methoxyphenyl)-1’,2’,3’-benzotriazin-4’(3*H*)-one]propanoic acid hydrochloride (**10e**) (0.017 g, 95%) as a yellow solid. Mp 208–211 °C; IR (neat) 3430, 2836, 2496, 1745, 1682, 1614, 1513, 1242, 1122, 980, 757 cm^{-1}; $[\alpha]_D^{23}$ –7.1 (*c* 0.1, MeOH); ^{1}H NMR (500 MHz, CD_3OD) δ 8.29 (d, 1H, *J* = 8.2 Hz), 8.27 (d, 1H, *J* = 1.2 Hz), 8.04 (dd, 1H, *J* = 8.2, 1.2 Hz), 7.45–7.39 (m, 2H), 7.13 (d, 1H, *J* = 8.2 Hz), 7.07 (t, 1H, *J* = 7.4 Hz), 5.08 (dd, 1H, *J* = 14.6, 4.5 Hz), 4.91 (dd, 1H, *J* = 14.6, 7.2 Hz), 4.60 (dd, 1H, *J* = 7.2, 4.5 Hz), 3.82 (s, 3H); ^{13}C{^{1}H} NMR (126 MHz, CD_3OD) δ 169.2 (C), 157.9 (C), 157.8 (C), 147.9 (C), 145.4 (C), 135.8 (CH), 131.77 (CH), 131.76 (CH), 129.8 (CH), 129.0 (C), 125.4 (CH), 122.3 (CH), 119.1 (C), 112.9 (CH), 56.1 (CH_3), 53.3 (CH), 49.8 (CH_2);

MS (ESI) *m/z* 363 (M + Na^+, 100); HRMS (ESI) *m/z*: $[M + Na]^+$ Calcd for $C_{17}H_{16}N_4O_4Na$ 363.1064; Found 363.1063.

(2*S*)-2-[(9*H*-Fluoren-9-ylmethoxycarbonyl)amino]-3-[7'-(2''-methoxyphenyl)-1',2',3'-benzotriazin-4'(3*H*)-one]propanoic acid (11).

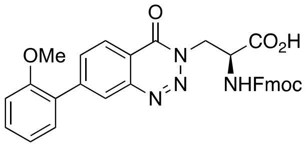

To a stirred solution of (2*S*)-2-amino-3-[7'-(2''-methoxyphenyl)-1',2',3'-benzotriazin-4'(3*H*)-one]propanoic acid hydrochloride (**10e**) (0.156 g, 0.414 mmol) in dioxane (1.5 mL) and water (1.5 mL) was added sodium hydrogen carbonate (0.139 g, 1.66 mmol) and *N*-(9-fluorenylmethoxycarbonyloxy)succinimide (0.137 g, 0.406 mmol). The reaction mixture was stirred at room temperature for 24 h and then concentrated *in vacuo.* The reaction mixture was diluted in water (10 mL) and acidified to pH 2 using 1 M aqueous hydrochloric acid and extracted with diethyl ether (2 × 20 mL) and ethyl acetate (3 × 20 mL). The organic layer was dried ($MgSO_4$), filtered and concentrated *in vacuo.* Purification by flash column chromatography eluting with acetone followed by 10% methanol in acetone gave (2*S*)-2-[(9*H*-fluoren-9-ylmethoxycarbonyl)amino]-3-[7'-(2''-methoxyphenyl)-1',2',3'-benzotriazin-4'(3*H*)-one]propanoic acid hydrochloride (**11**) (0.173 g, 76%) as a white solid. Mp 181–185 °C; IR (neat) 3314, 2160, 2029, 1728, 1670, 1601, 1404, 1242, 1022, 741 cm^{-1}; $[\alpha]_D^{18}$ –35.9 (*c* 0.1, DMSO); ^1H NMR (400 MHz, DMSO-d_6) δ 8.20 (d, 1H, *J* = 8.2 Hz), 8.10 (br s, 1H), 7.94 (dd, 1H, *J* = 8.2, 1.7 Hz), 7.85 (d, 2H, *J* = 7.6 Hz), 7.60 (d, 1H, *J* = 7.5 Hz), 7.47 (t, 2H, *J* = 7.6 Hz), 7.43–7.26 (m, 4H), 7.22 (t, 1H, *J* = 7.5 Hz), 7.18 (d, 1H, *J* = 8.3 Hz), 7.08 (t, 1H, *J* = 7.5 Hz), 6.76 (br s, 1H), 4.91–5.04 (m, 1H), 4.39–4.27 (m, 2H), 4.07 (dd, 1H, *J* = 10.1, 6.7 Hz), 4.02–3.95 (m, 1H), 3.85 (dd, 1H, *J* = 10.1, 6.7 Hz), 3.76 (s, 3H); ^{13}C NMR data unavailable due to partial decomposition in DMSO-d_6 over time;[3] MS (ESI) *m/z* 563 (M + H^+, 100); HRMS (ESI) *m/z*: $[M + H]^+$ Calcd for $C_{32}H_{26}N_4O_6H$ 563.1925; Found 563.1918.

Synthesis of Peptide 12

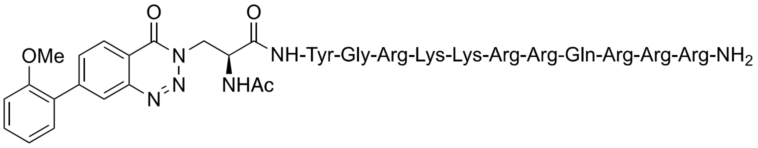

Peptide **12** was synthesized on a Biotage Initiator+ Alstra peptide synthesizer using an Fmoc/^{t}Bu protecting group strategy (0.1 mmol synthetic scale) and Rink Amide ChemMatrix© resin (0.45

mmol/g). Microwave-assisted SPPS was used for all coupling reactions. Following resin swelling in DMF at 70 °C for 20 min, the resin bound peptide was synthesized by first loading Fmoc-Arg(Pbf)-OH to the resin and by introduction of the Fmoc-protected amino acids (5 equiv., 0.2 M in DMF) successively by treatment with DIC (5 equiv., 0.5 M in DMF) and OxymaPure (5 equiv., 0.5 M in DMF) at 90 °C for 2 min. Arg residues were coupled at room temperature for 45 min, followed by 90 °C for 5 min, then double coupled at 90 °C for 10 min. Fmoc groups were removed using morpholine (20% in DMF with 5% formic acid, 4 mL) at 90 °C for 1 min. The resin was washed with DMF between deprotection and coupling (4 × 4 mL) and after coupling (2 × 4 mL). The Fmoc-deprotected resin bound peptide was suspended in DMF (2 mL) followed by addition of (2*S*)-2-[(9*H*-fluoren-9-ylmethoxycarbonyl)amino]-3-[7'-(2''-methoxyphenyl)-1',2',3'-benzotriazin-4'(3*H*)-one]propanoic acid hydrochloride (**11**) (2 equiv., 0.2 M in DMF), DIC (2 equiv., 0.5 M in DMF) and OxymaPure (2 equiv., 0.5 M in DMF). The reaction mixture was agitated at 75 °C for 10 min. The solution was filtered, and the resin was washed with DMF (2 × 5 mL) and dichloromethane (2 × 5 mL). *N*-Terminal acetylation was achieved on-resin with acetic anhydride (3 equiv.), DIPEA (4.5 equiv.) and DMF (7 mL for 0.1 mmol of resin) for 20 min with agitation. The resin was then washed with DMF (3 × 5 mL) and dichloromethane (3 × 5 mL). Peptide cleavage was carried out using a cleavage cocktail (10 mL) of trifluoroacetic acid (95%), triisopropylsilane (2.5%) and water (2.5%). The reaction mixture was agitated at room temperature for 4 h. The resin was filtered, and the cleavage cocktail evaporated using a stream of N_2. Peptide **12** was precipitated from a solution of cold diethyl ether, centrifuged (4500 rpm for 5 min) and washed with ice-cold diethyl ether. Peptide **12** was dissolved in a mixture of water and acetonitrile with 0.1% TFA and lyophilized on a Christ Alpha 1-2 LD plus freeze dryer.

Purification: Peptide **12** was purified using RP-HPLC on an Agilent Technologies 1260 Infinity RP-HPLC system (monitoring at 214 nm and 280 nm) with a Phenomenex Gemini column (5 mm C18, 250 × 21.2 mm). A gradient was run using a solvent system consisting of A (water + 0.1% TFA) and B (MeCN + 0.1% TFA), and collected fractions were lyophilized on a Christ Alpha 1-2 LD plus freeze dryer. Peptide **12** was analyzed on a Shimadzu RP-HPLC system with Shimadzu LC-20AT pumps, a Shimadzu SIL20A autosampler and a Shimadzu SPD-20A UV-vis detector using a Phenomenex Aeris column (5 mm C18, 100 Å, 150 × 10 mm). An RP-HPLC gradient was run using a solvent system consisting of solution A (0.1% TFA in water) and B (0.1% TFA in MeCN). Two gradients were used to characterize peptide **12**, a gradient from 5–95% solution B over 50 minutes and a gradient from 5–95% solution B over 20 minutes. Analytical RP-HPLC data is reported as column retention time (t_R) in minutes (see table below). High-resolution mass spectrometry (HRMS) was performed on an Agilent 6546 LC/Q-TOF in positive mode (ESI+). HRMS data are reported as mass to charge ratio (*m/z*) = observed / MW.

Peptide	Purity by RP-HPLC	Yield (%)	t_R (20 min, 50 min gradient)	Calculated *m*/*z*	Measured *m*/*z*	Error (ppm)
**12**	>99%	3	12.043, 21.646	$[M + 3H]^{+3}$ = 641.7018	$[M + 3H]^{+3}$ = 641.7013	–0.89

50 min gradient HPLC for peptide **12:**

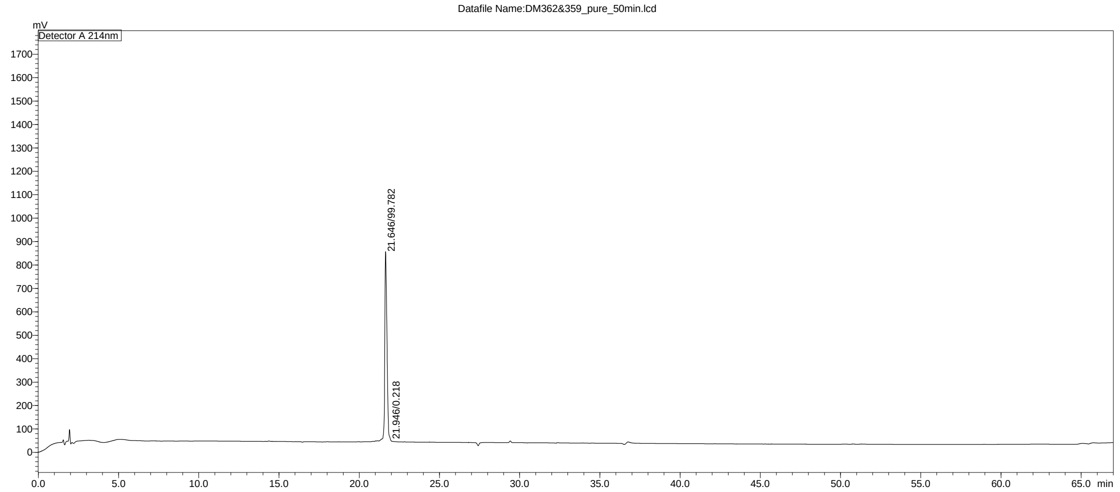

20 min gradient HPLC for peptide **12:**

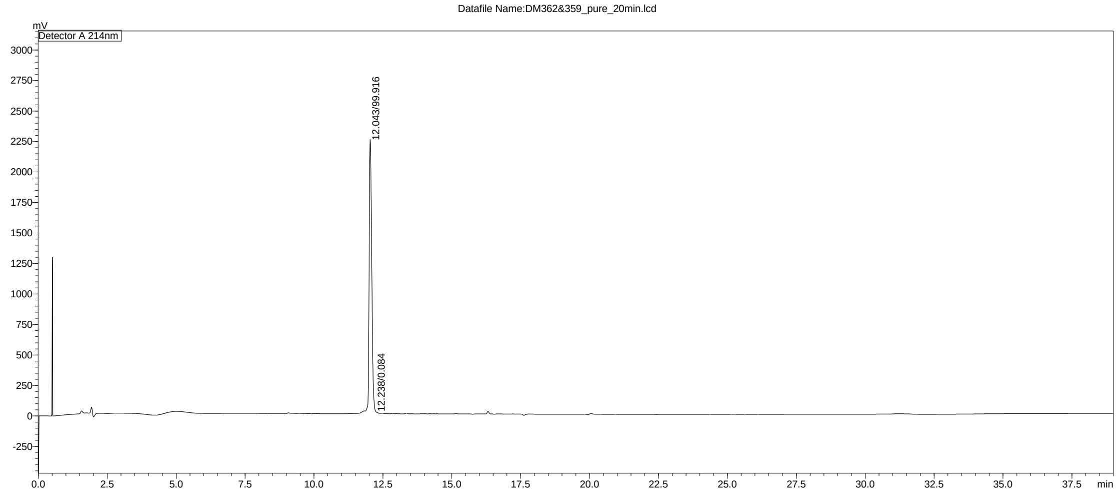

HRMS for peptide **12:**

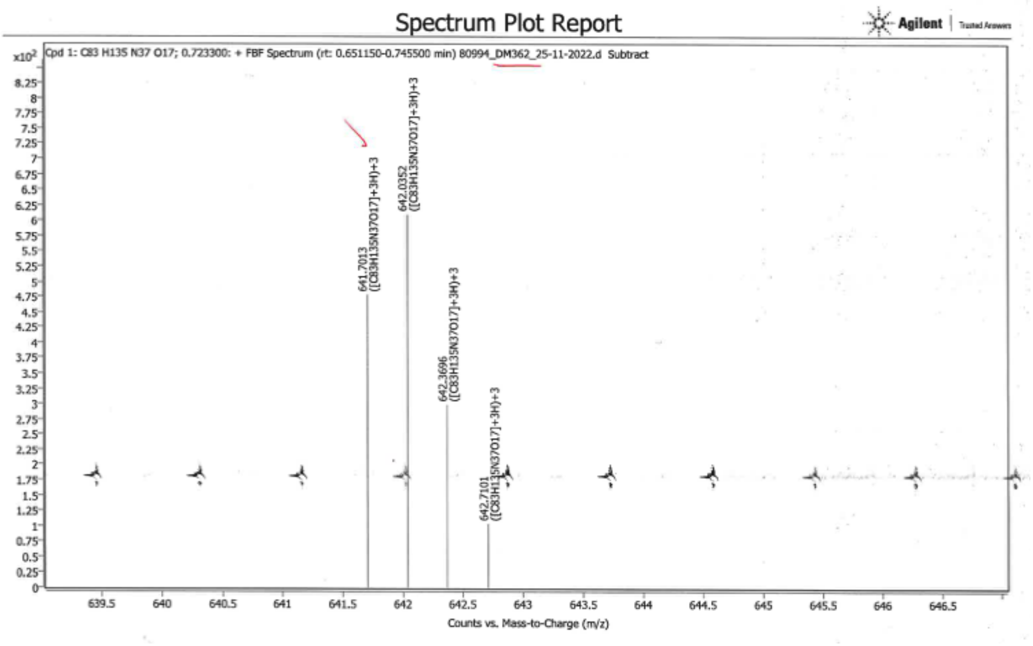

3. Photophysical Data for α-Amino Acids 10a–e and Peptide 12

Spectra were recorded at 15 μM in methanol using an excitation and emission slit width of 5 nm.

Absorption and Emission Spectra for 10a.

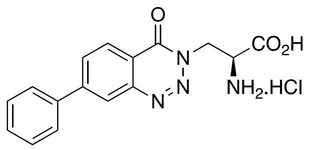

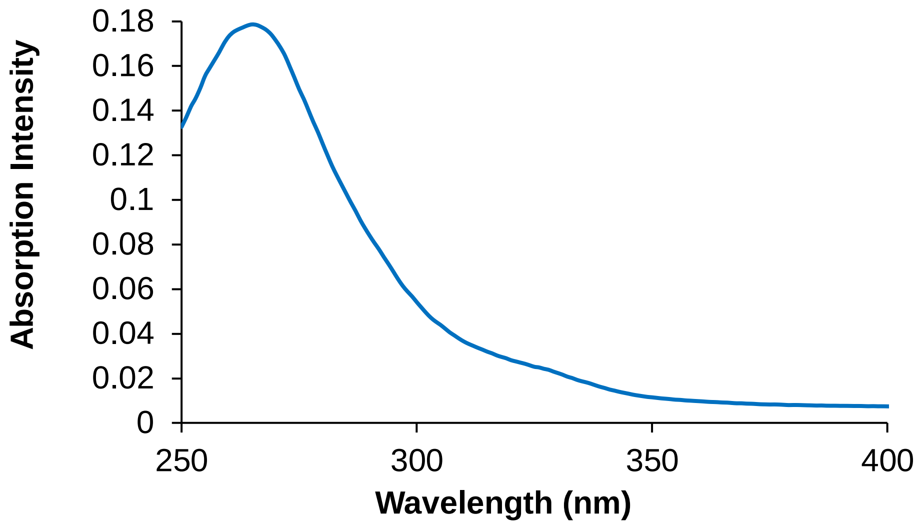

Excitation at 266 nm:

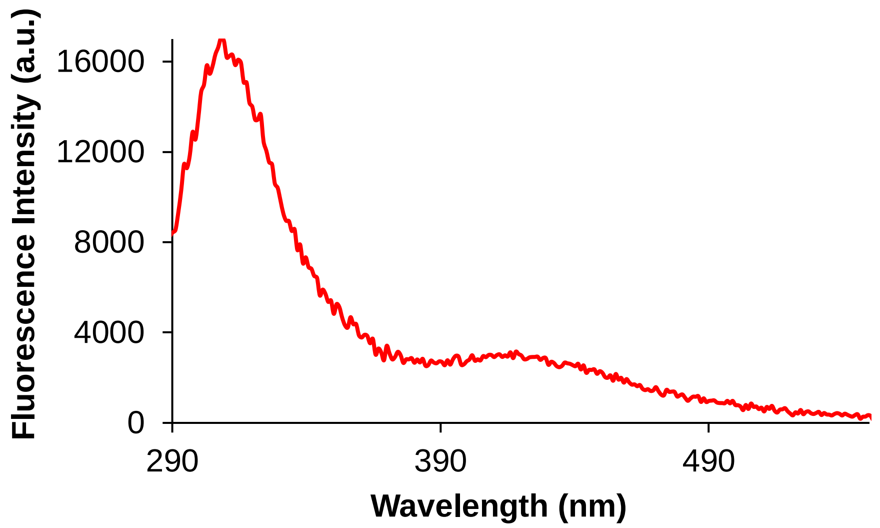

Absorption and Emission Spectra for 10b.

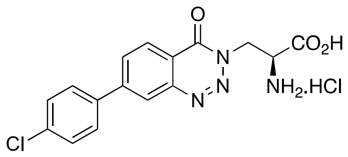

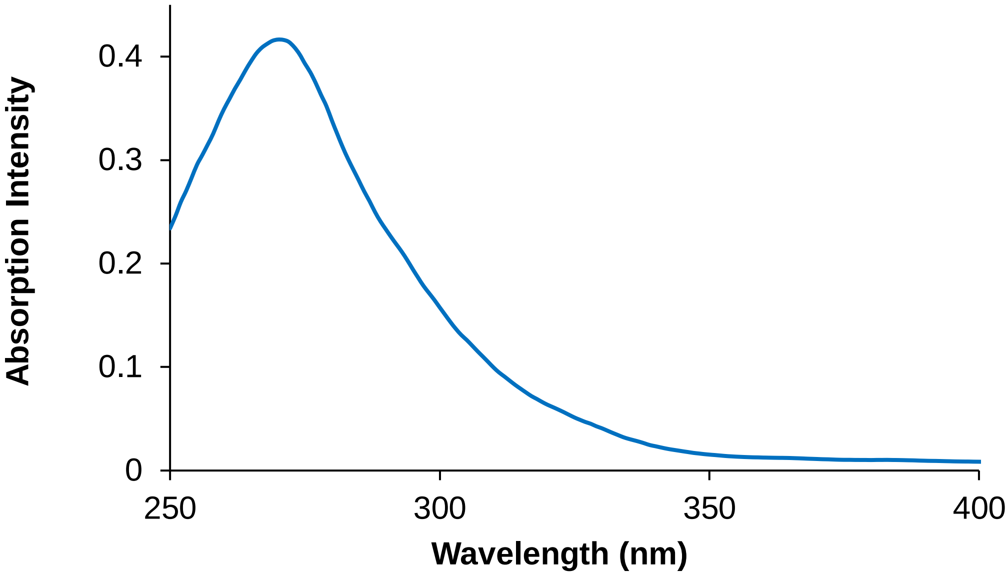

Excitation at 270 nm:

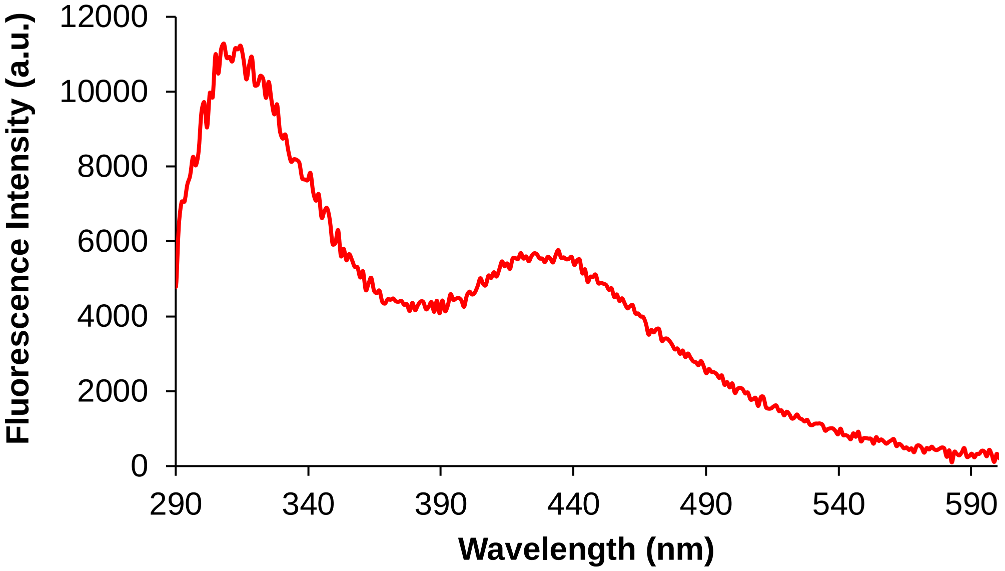

Absorption and Emission Spectra for 10c.

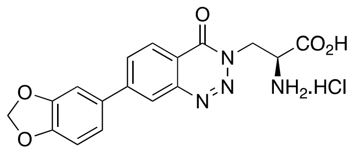

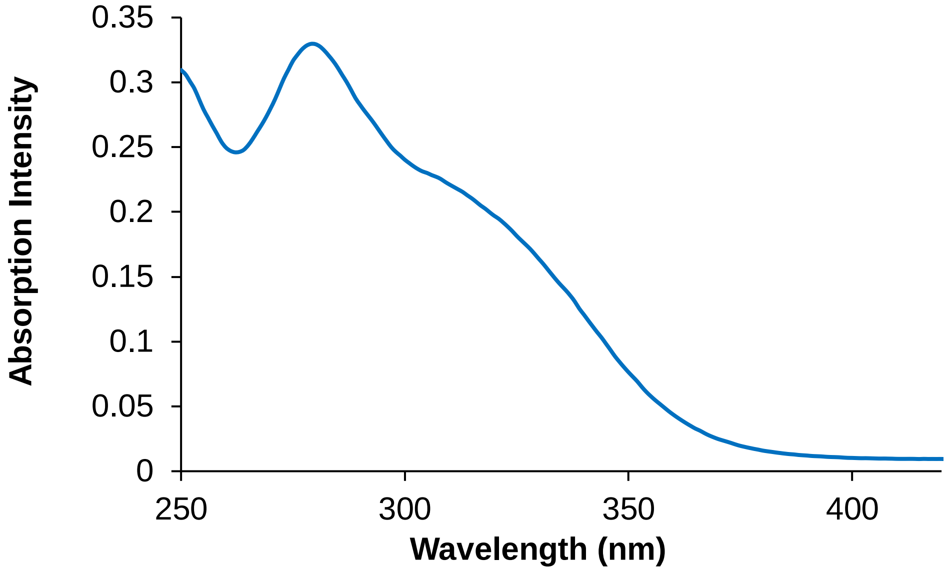

Excitation at 279 nm:

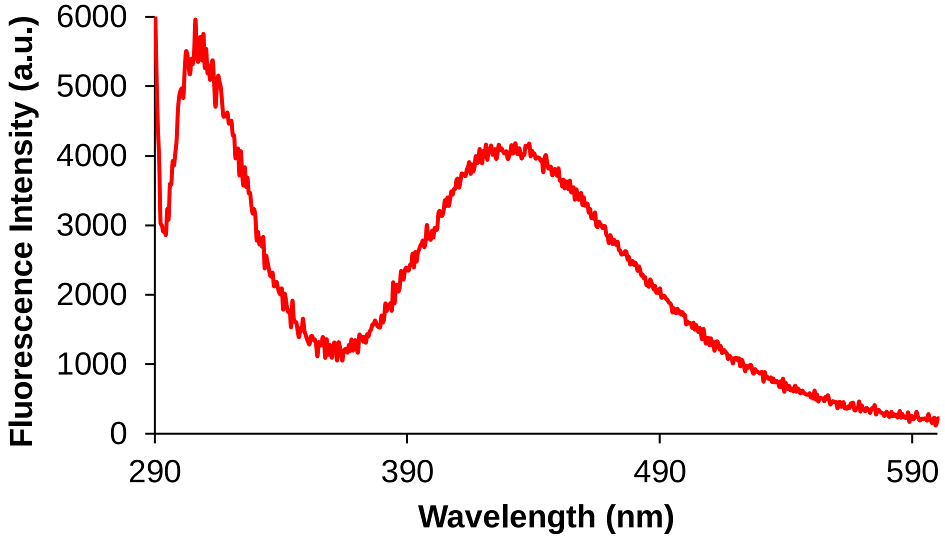

Absorption and Emission Spectra for 10d.

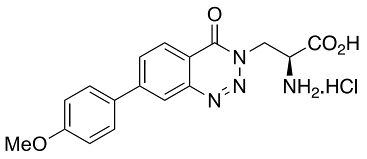

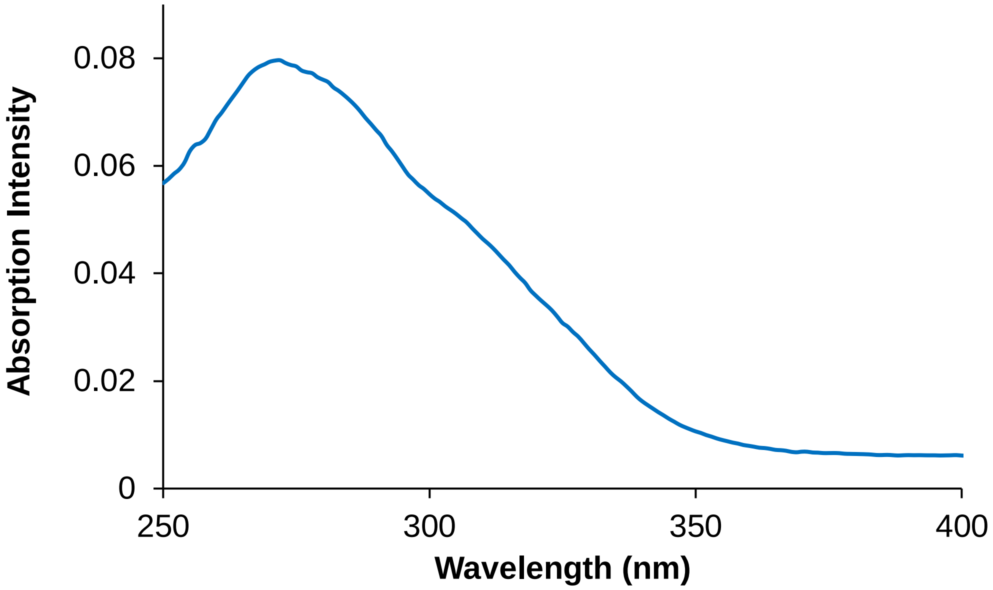

Excitation at 269 nm:

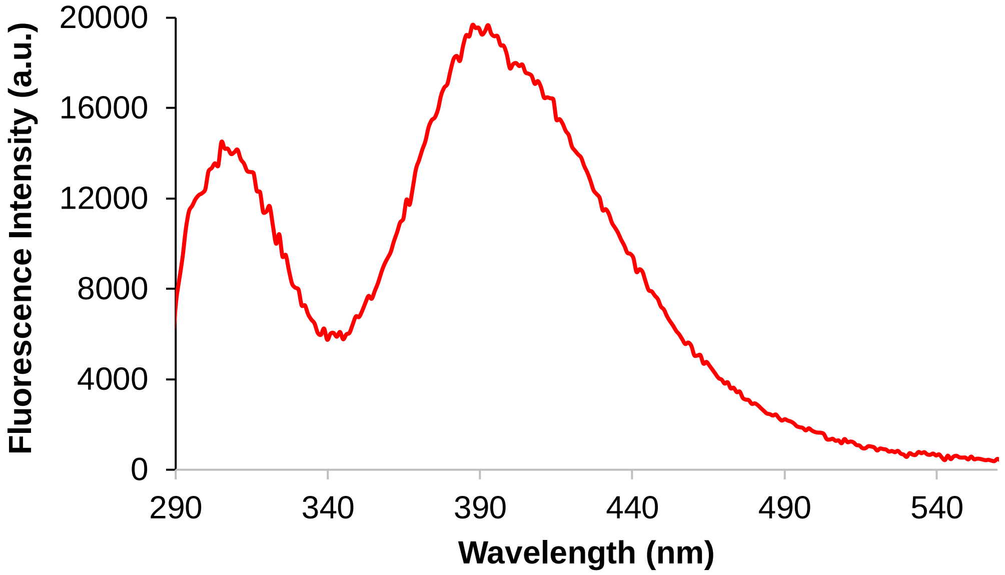

Absorption and Emission Spectra for 10e.

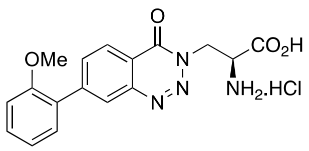

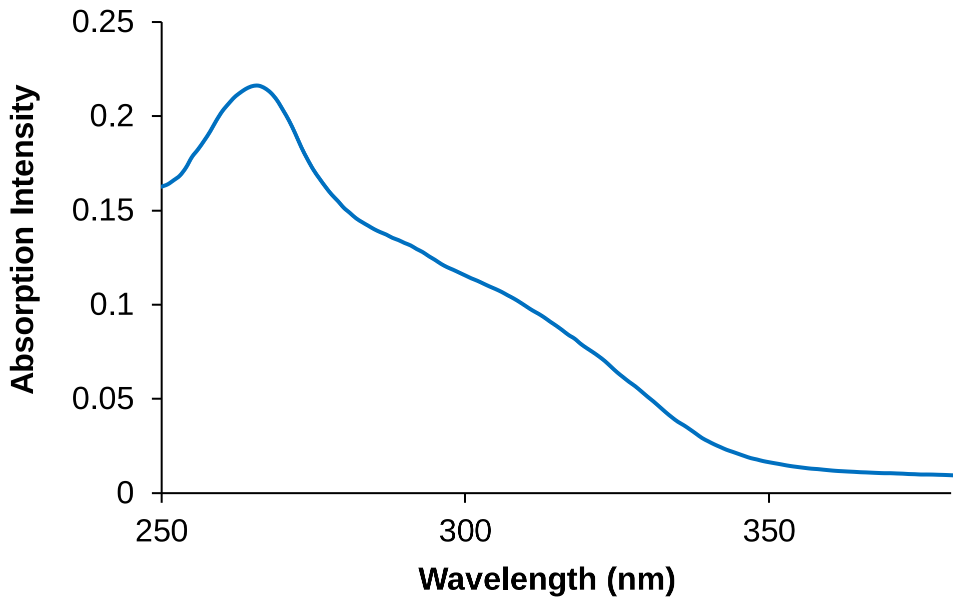

Excitation at 266 nm:

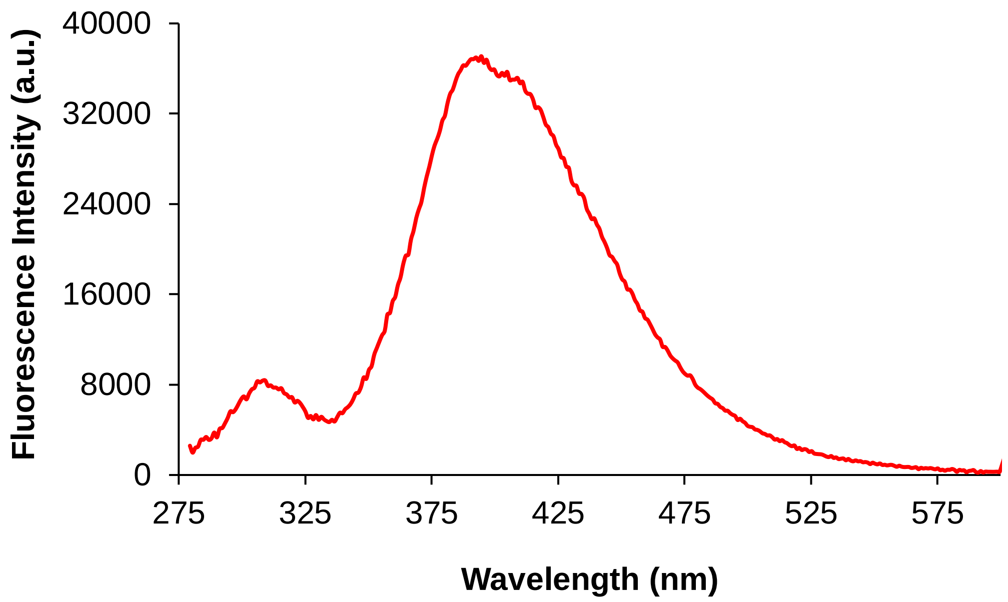

Excitation Spectra for Amino Acids 10a–10e.

The excitation spectra were recorded on a Shimadzu RF-5301PC spectrofluorophotometer at each wavelength of the two emission maxima for amino acids **10a–10e** and at a concentration of 15 μM.

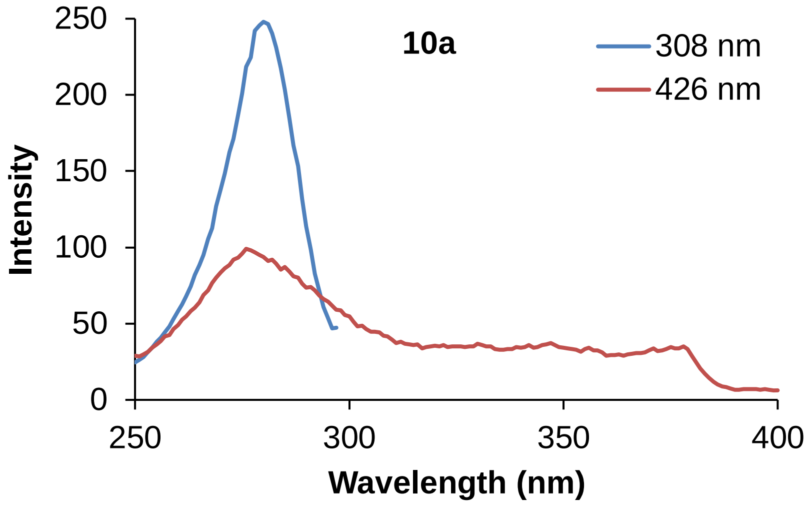

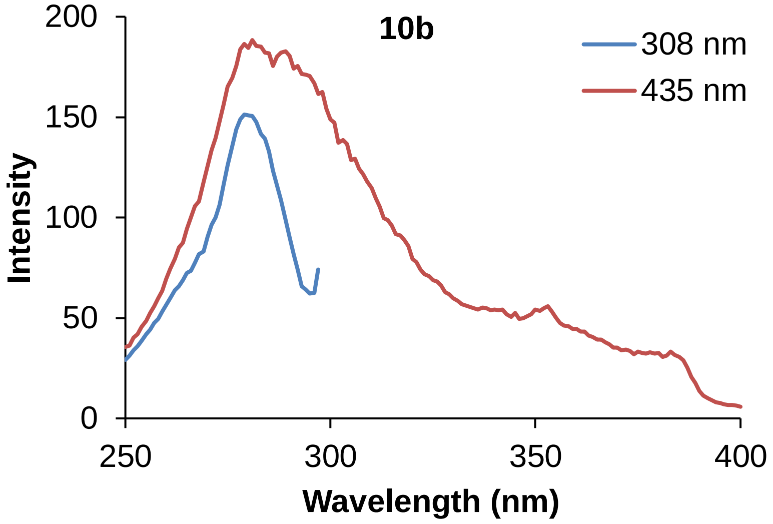

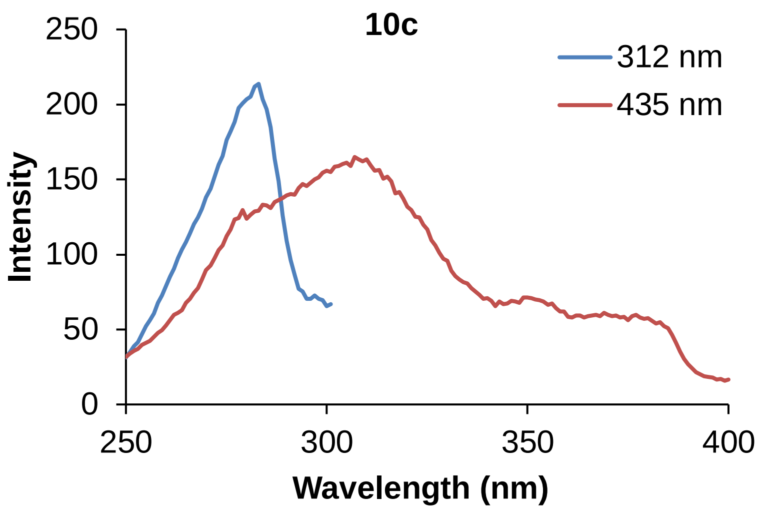
10c
312 nm
435 nm
Intensity
0
50
100
150
200
250
250
300
350
400
Wavelength (nm)

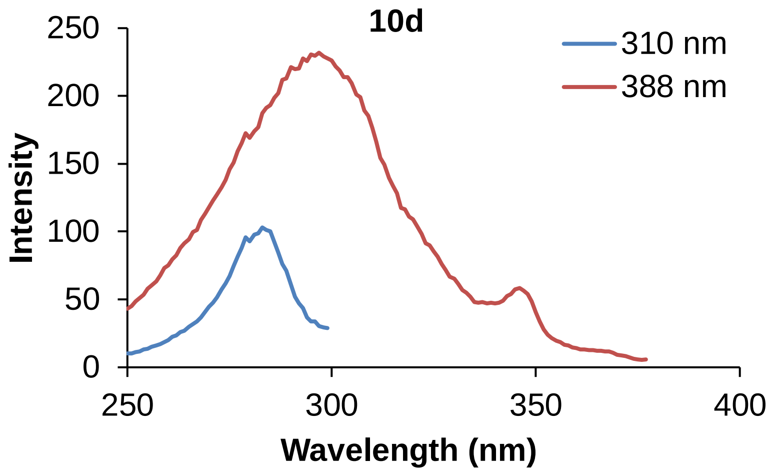
10d
310 nm
388 nm
Intensity
0
50
100
150
200
250
250
300
350
400
Wavelength (nm)

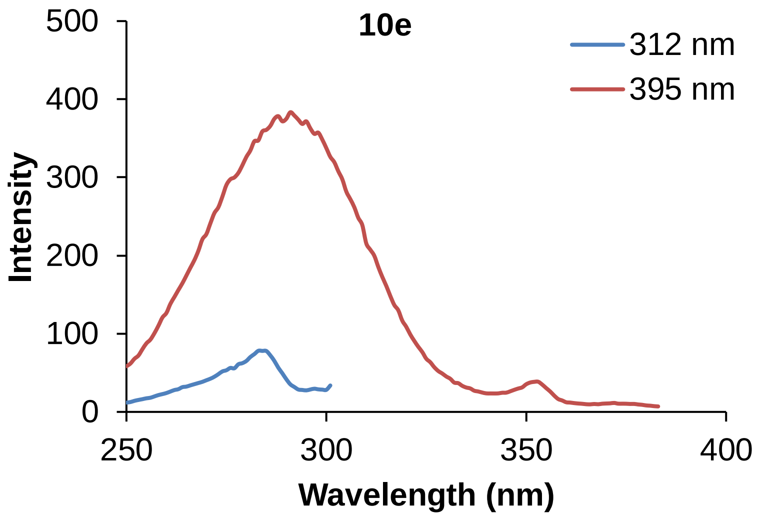
10e
312 nm
395 nm
Intensity
0
100
200
300
400
500
250
300
350
400
Wavelength (nm)

Absorption and Emission Spectra for Solvatochromic Study of 10e.

All spectra were recorded using a concentration of 5 μM.

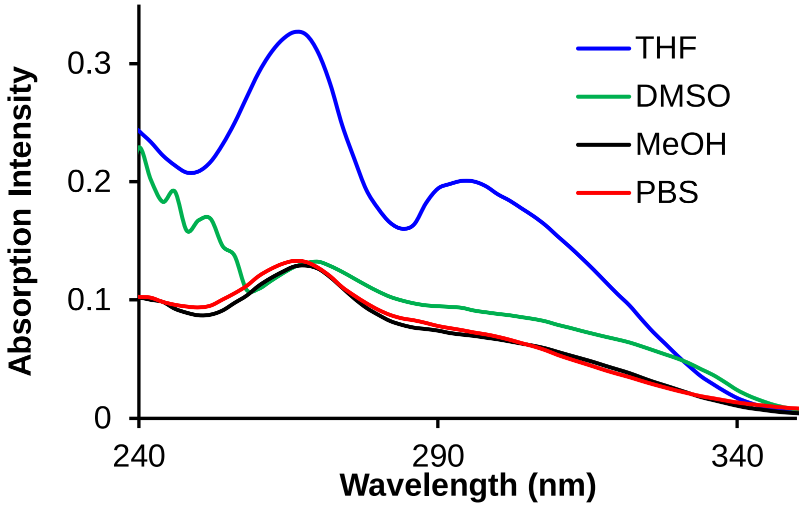

Excitation at 267 nm:

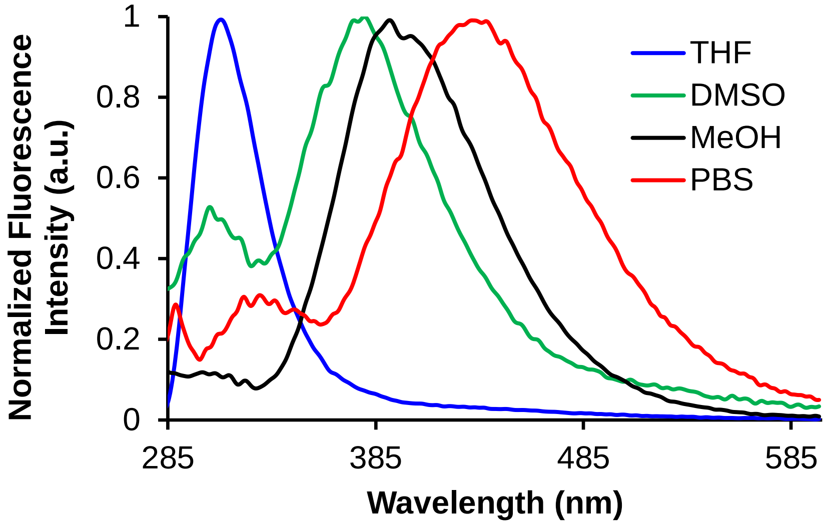

Lippert-Magata Plot for 10e.

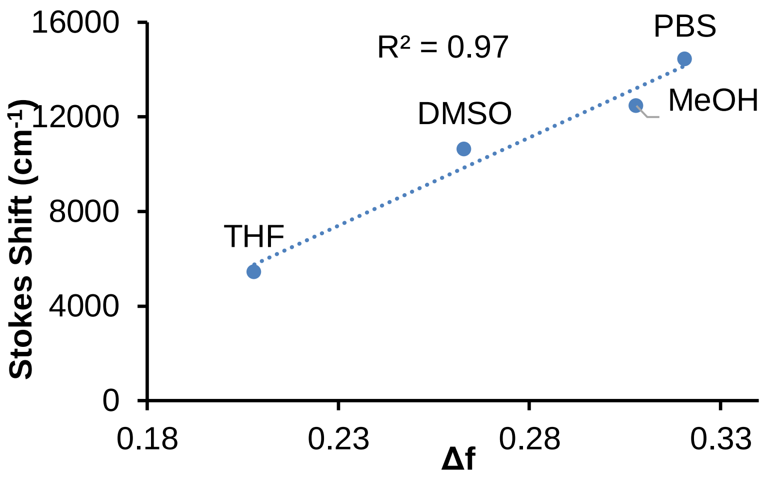

Absorption and Emission Spectra for pH Study of 10e.

To evaluate the effect of pH on the fluorescent properties of **10e**, absorption and emission spectra were obtained in neutral methanol (pH 7). Quantities of concentrated hydrochloric acid (3 M in MeOH) were then added to the stock solution to obtain samples at pH 4 and pH 1. All spectra were recorded at room temperature and using a concentration of 5 μM.

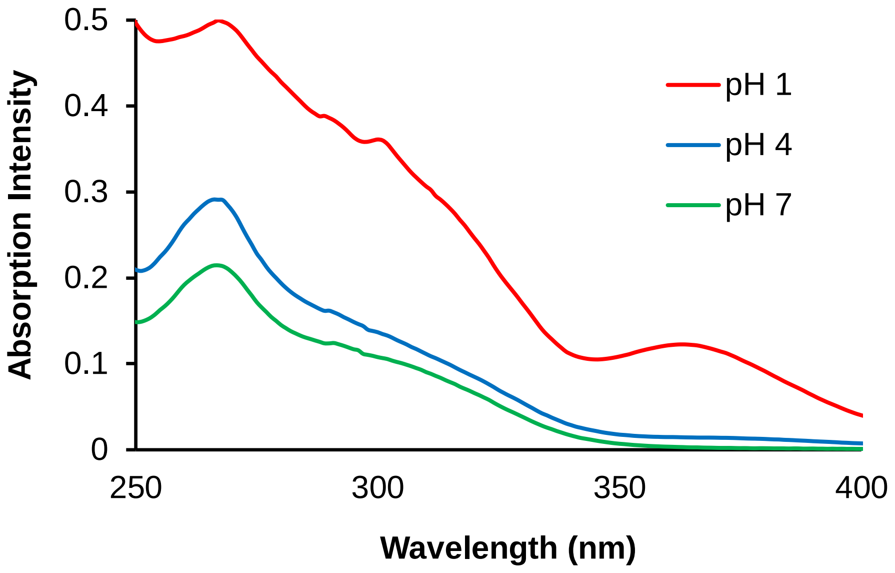

Excitation at 267 nm:

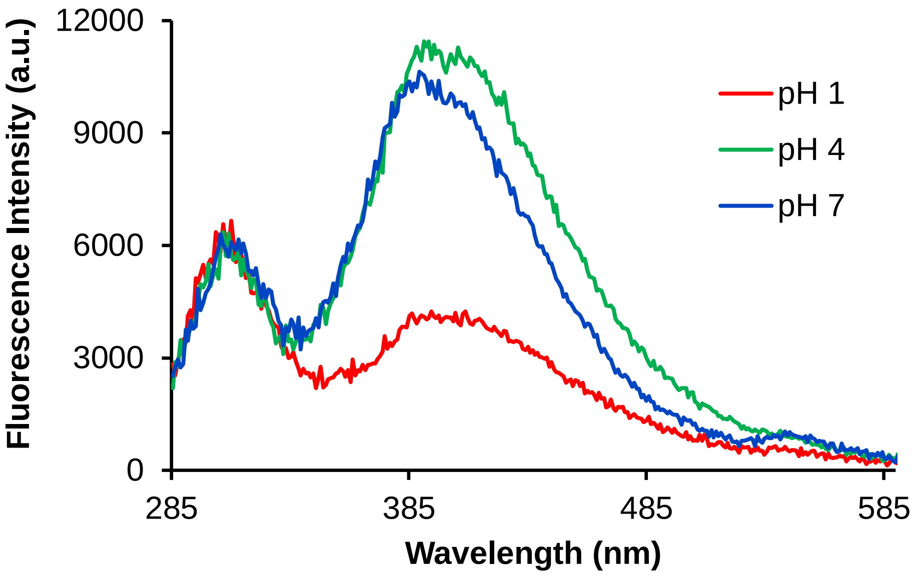

Absorption and Emission Spectra for peptide 12 (using a concentration of 7 μM in MeOH).

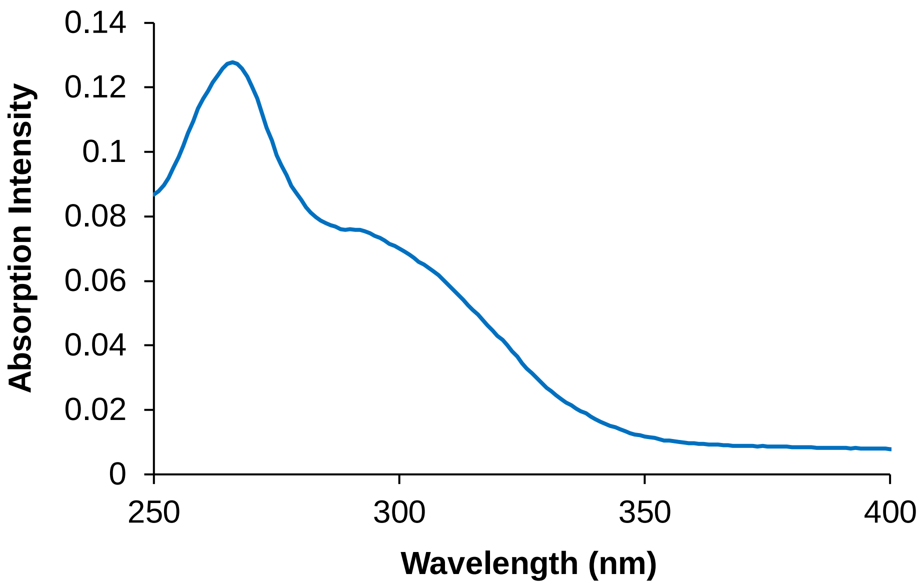

Excitation at 306 nm:

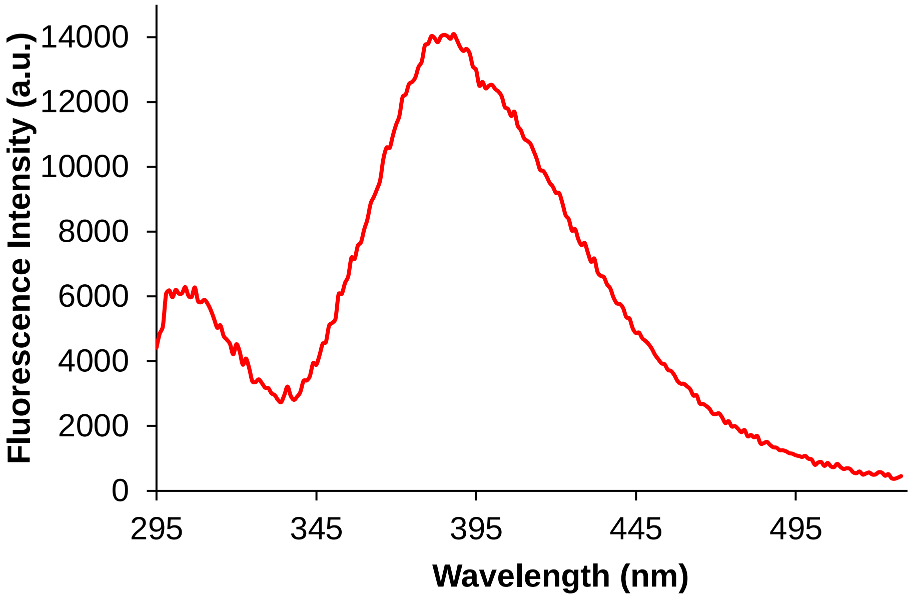

4. References

1. Stojkovic, M. R.; Piotrowski, P.; Schmuck, C.; Piantanida, I. A Short, Rigid Linker Between Pyrene and Guanidiniocarbonyl-Pyrrole Induced a New Set of Spectroscopic Responses to the ds-DNA Secondary Structure. *Org. Biomol. Chem*. **2015**, *13*, 1629–1633.

2. Williams, A. T. R.; Winfield, S. A.; Miller, J. N. Relative Fluorescence Quantum Yields Using a Computer-controlled Luminescence Spectrometer. *Analyst* **1983**, *108*, 1067–1071.

3. Höck, S.; Marti, R.; Riedl, R.; Simeunovic, M. Thermal Cleavage of the Fmoc Protection Group. *Chimia* **2010**, *64*, 200–202.

5. ^{1}H and ^{13}C NMR Spectra for all Novel Compounds

^{1}H NMR (500 MHz, $CDCl_3$)

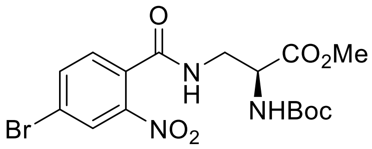

6

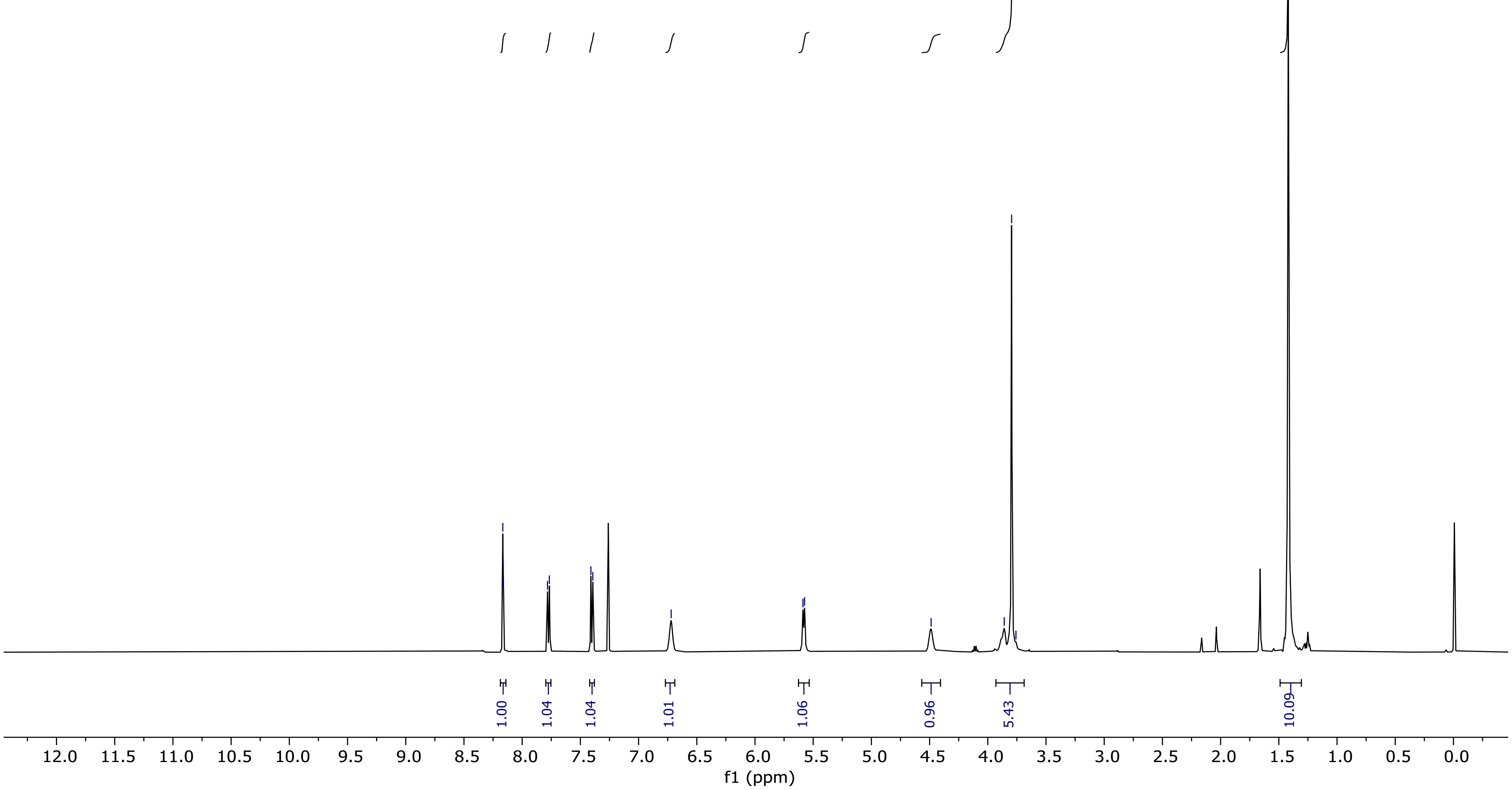

$^{13}C\{^{1}H\}$ NMR (126 MHz, $CDCl_3$)

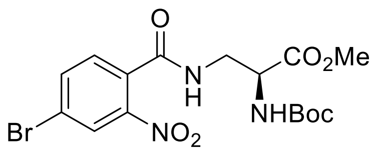

6

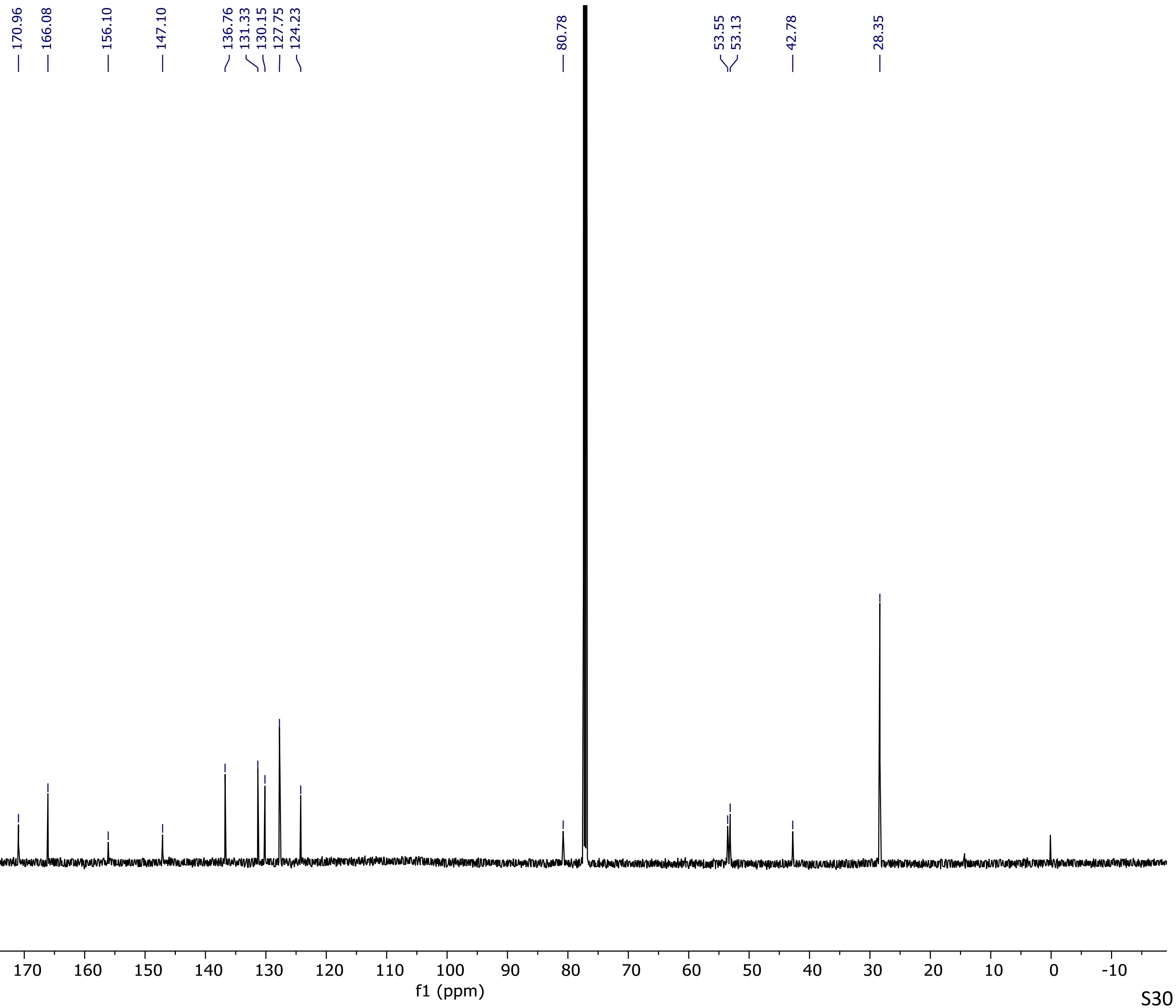

^{1}H NMR (500 MHz, CDCl$_3$)

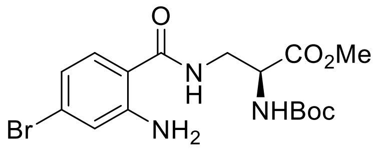

7

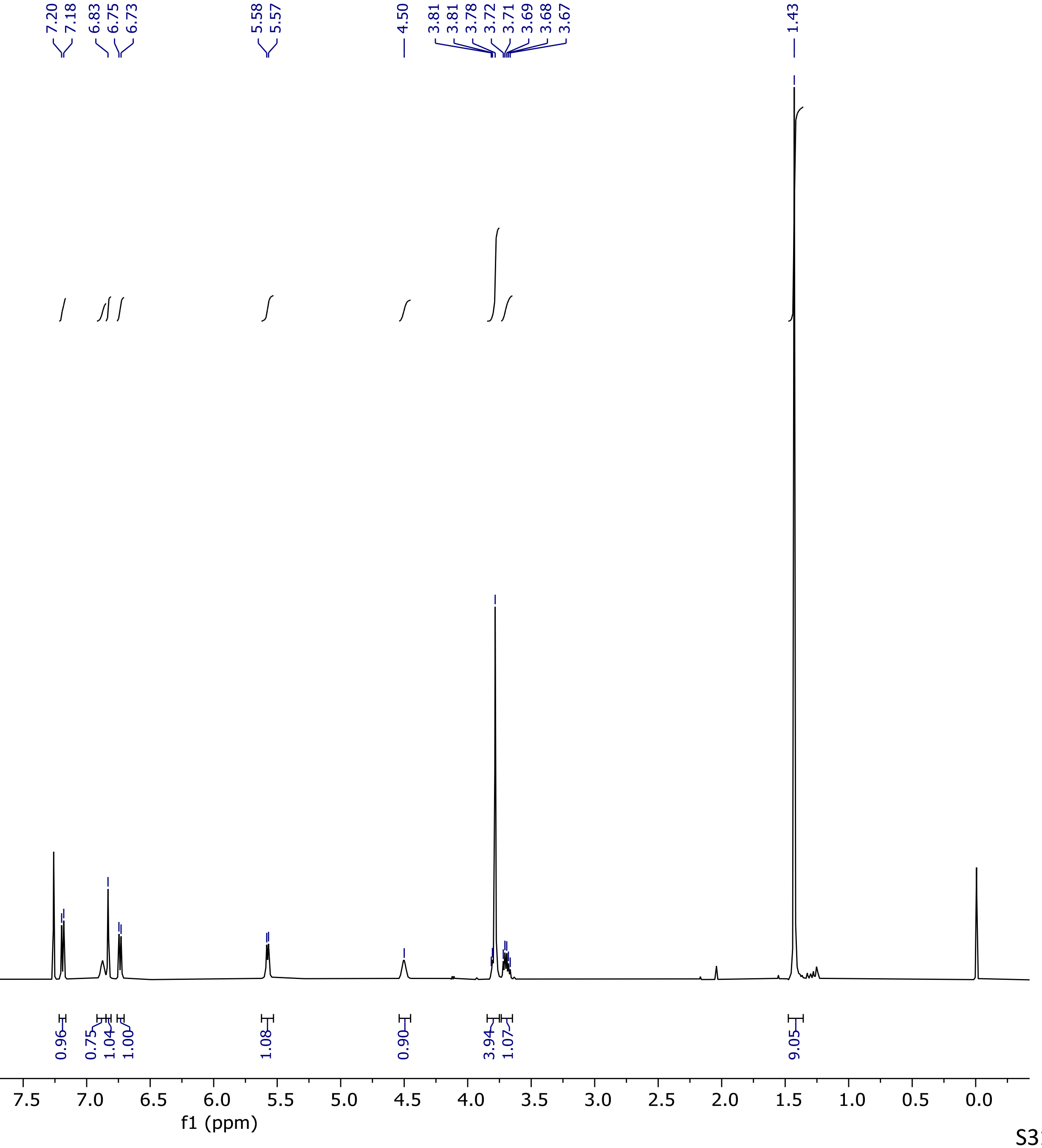

$^{13}C\{^{1}H\}$ NMR (126 MHz, $CDCl_3$)

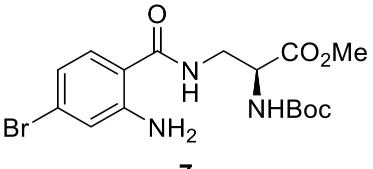

7

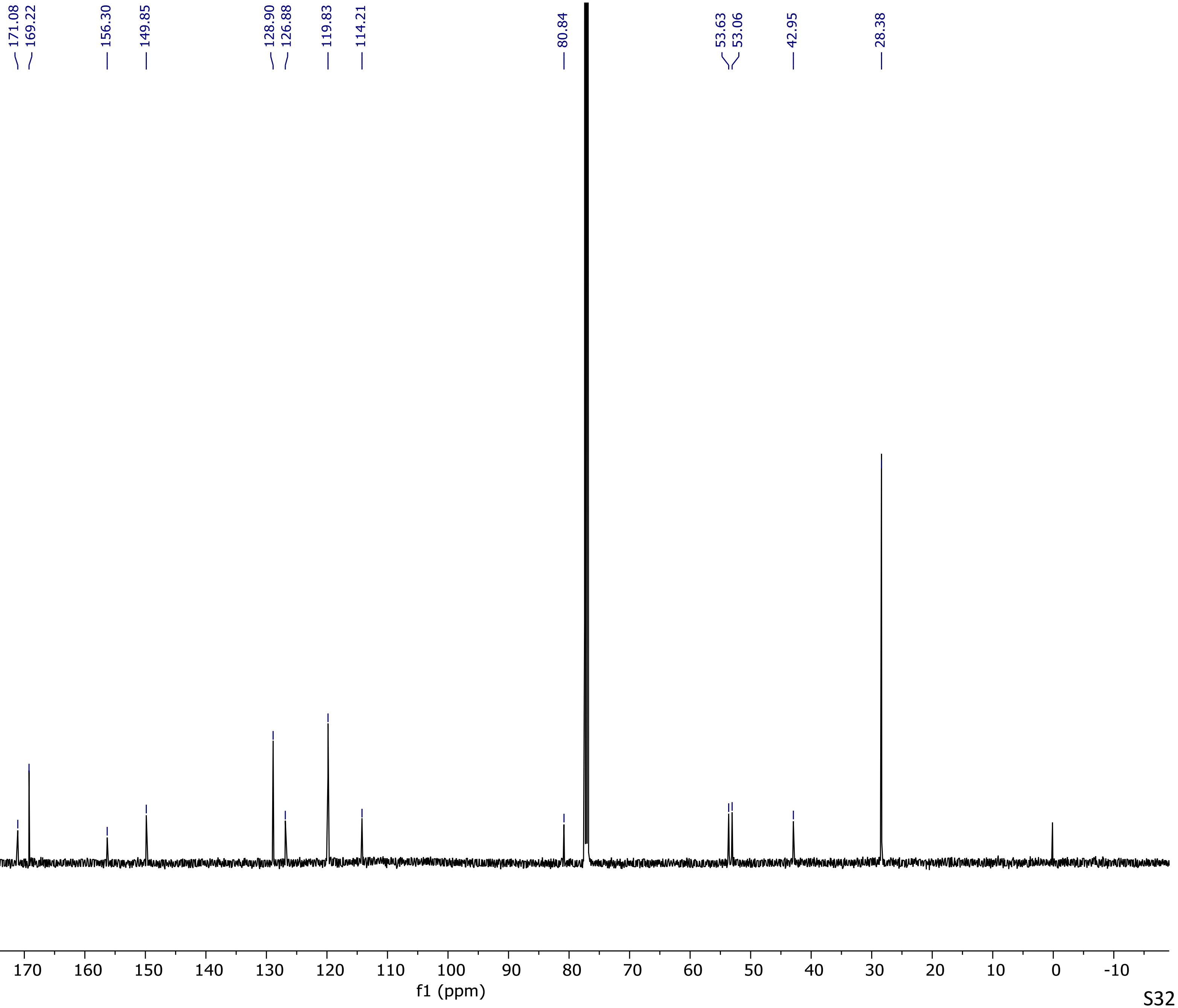

^{1}H NMR (500 MHz, $CDCl_3$)

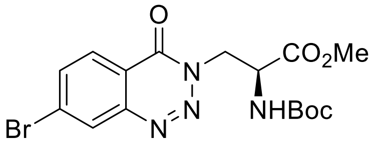

8

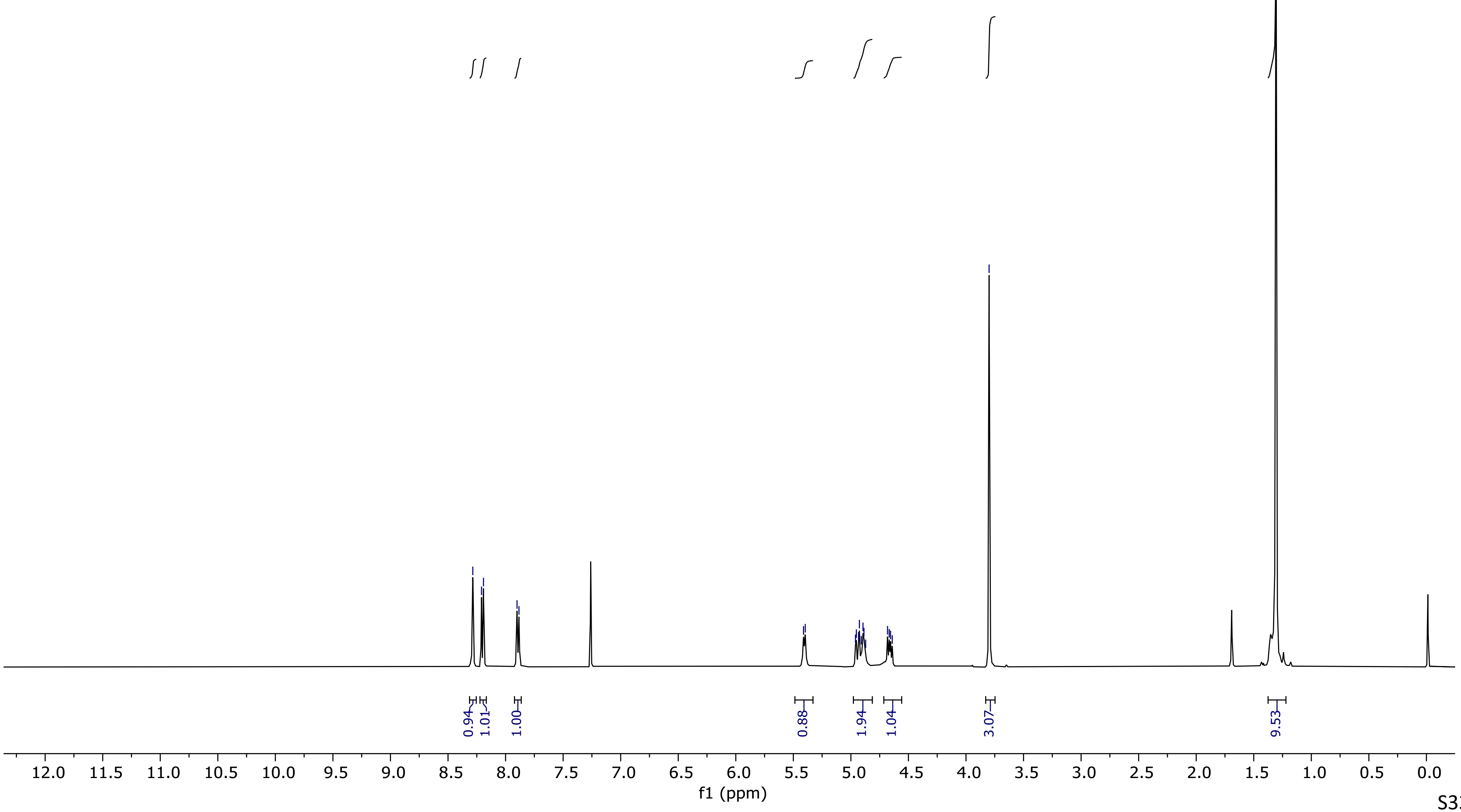

$^{13}C\{^1H\}$ NMR (126 MHz, $CDCl_3$)

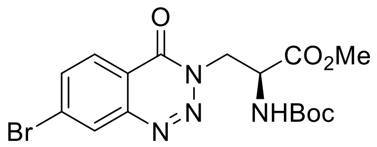

8

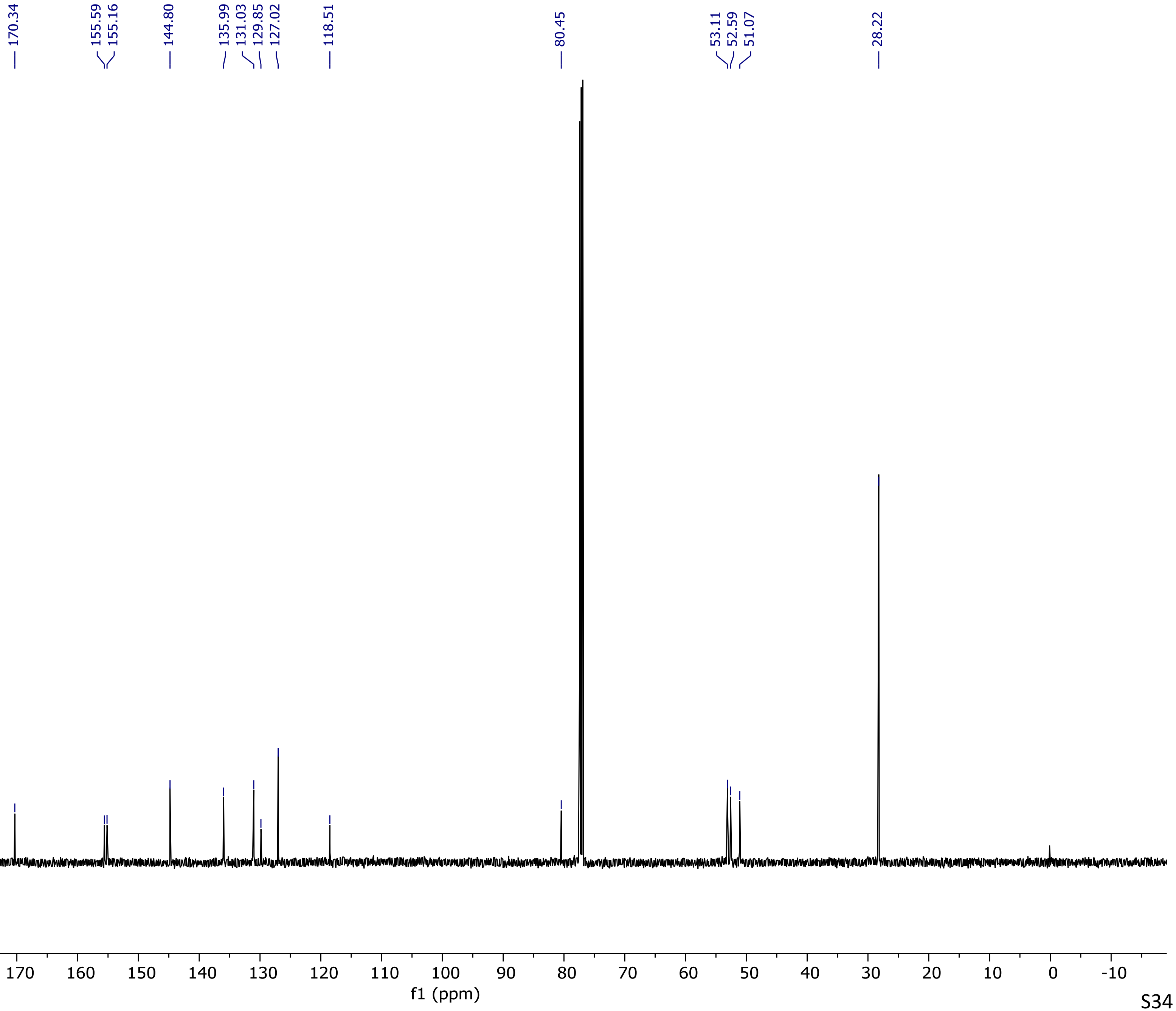

^{1}H NMR (400 MHz, $CDCl_3$)

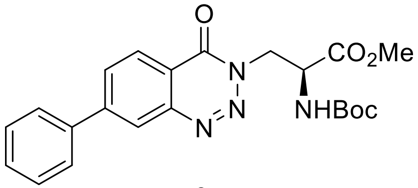

9a

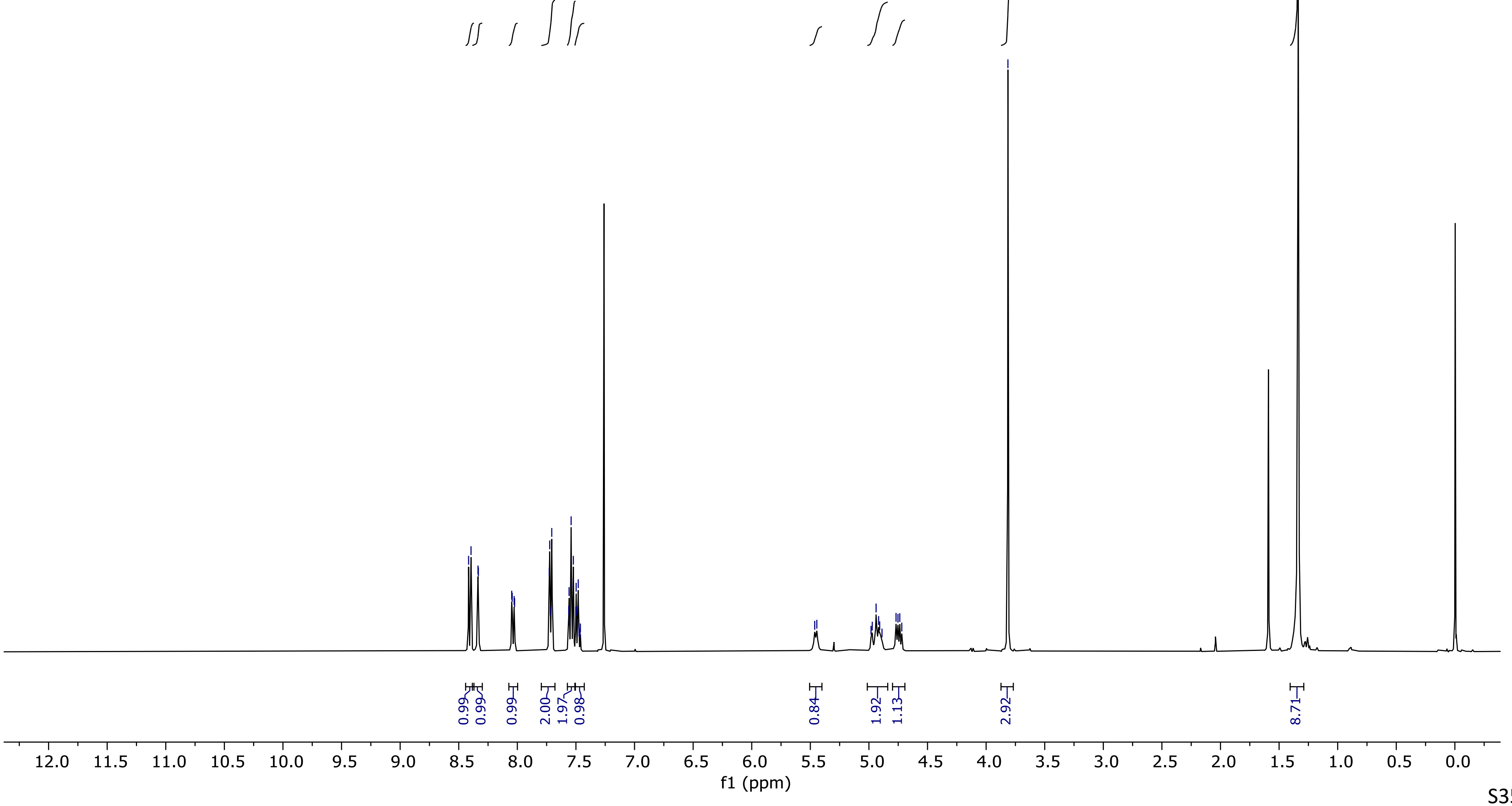

$^{13}C\{^1H\}$ NMR (101 MHz, $CDCl_3$)

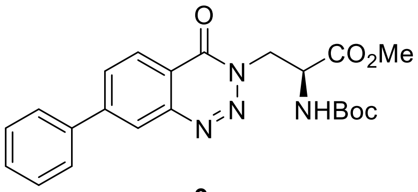

9a

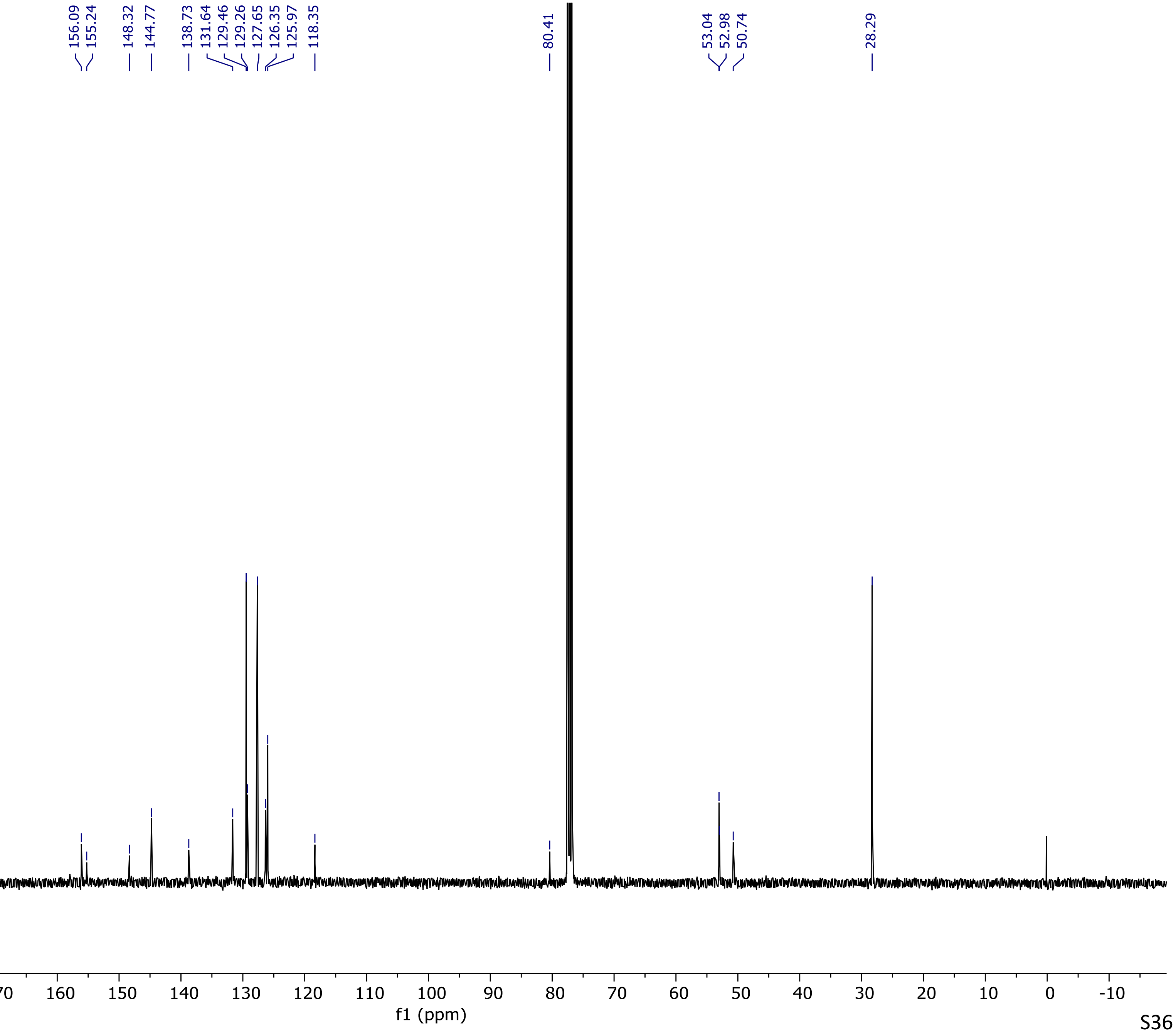

^{1}H NMR (400 MHz, $CDCl_3$)

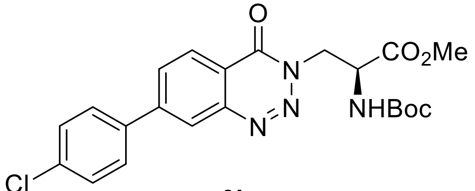

9b

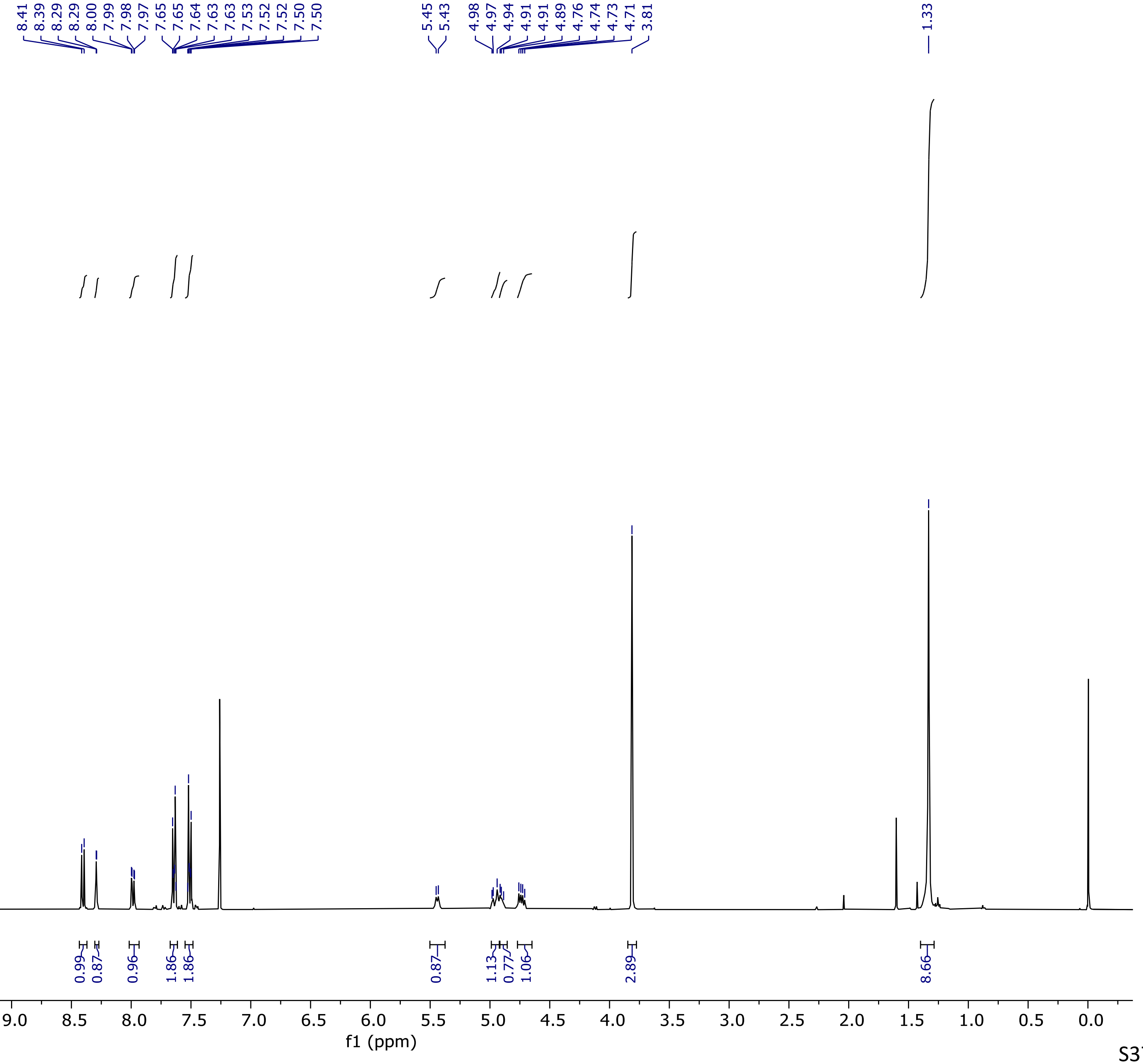

$^{13}C\{^1H\}$ NMR (101 MHz, CDCl$_3$)

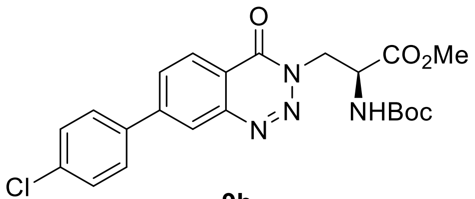

9b

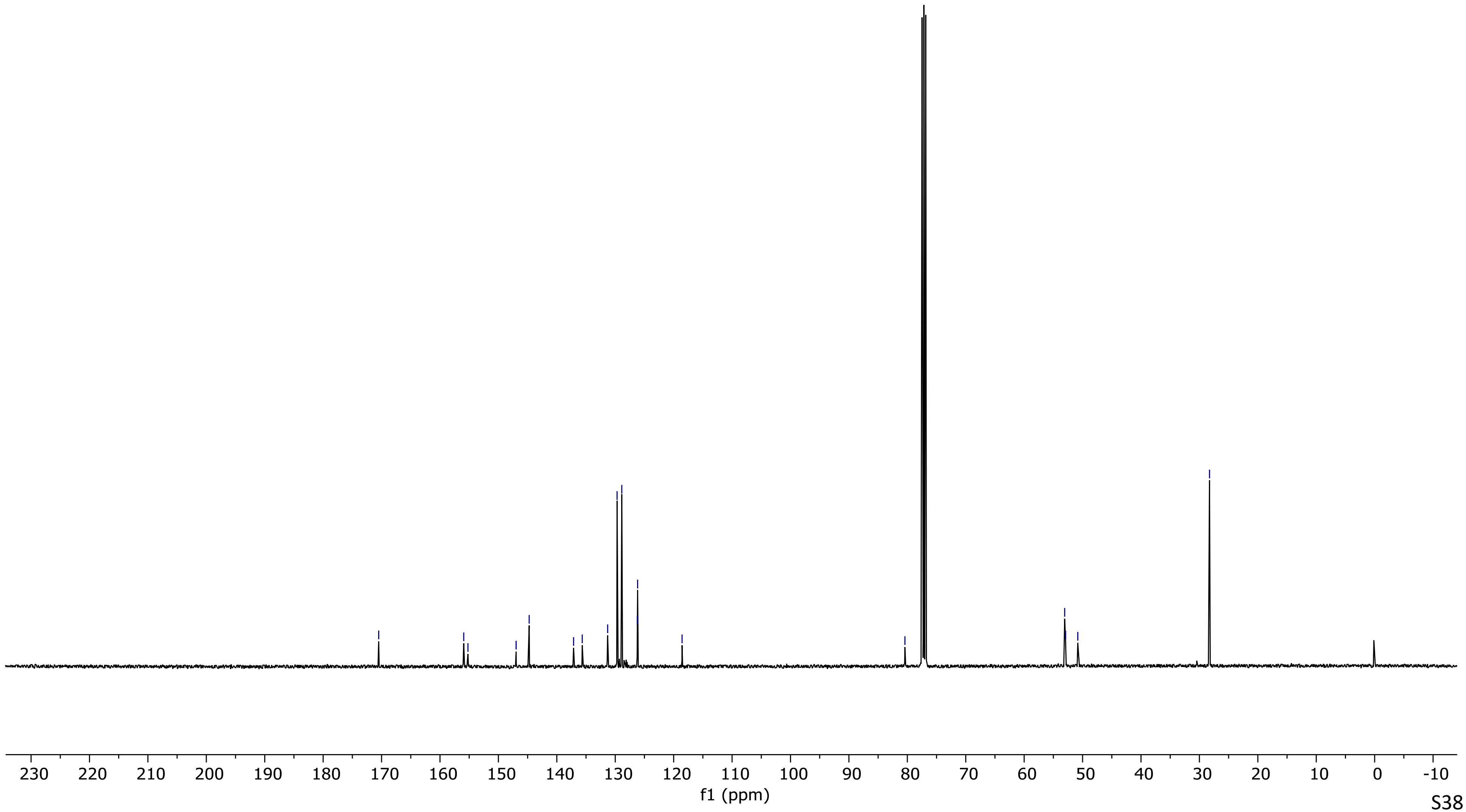

^{1}H NMR (400 MHz, CDCl$_3$)

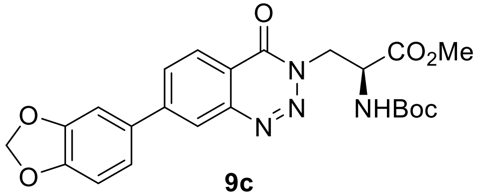

9c

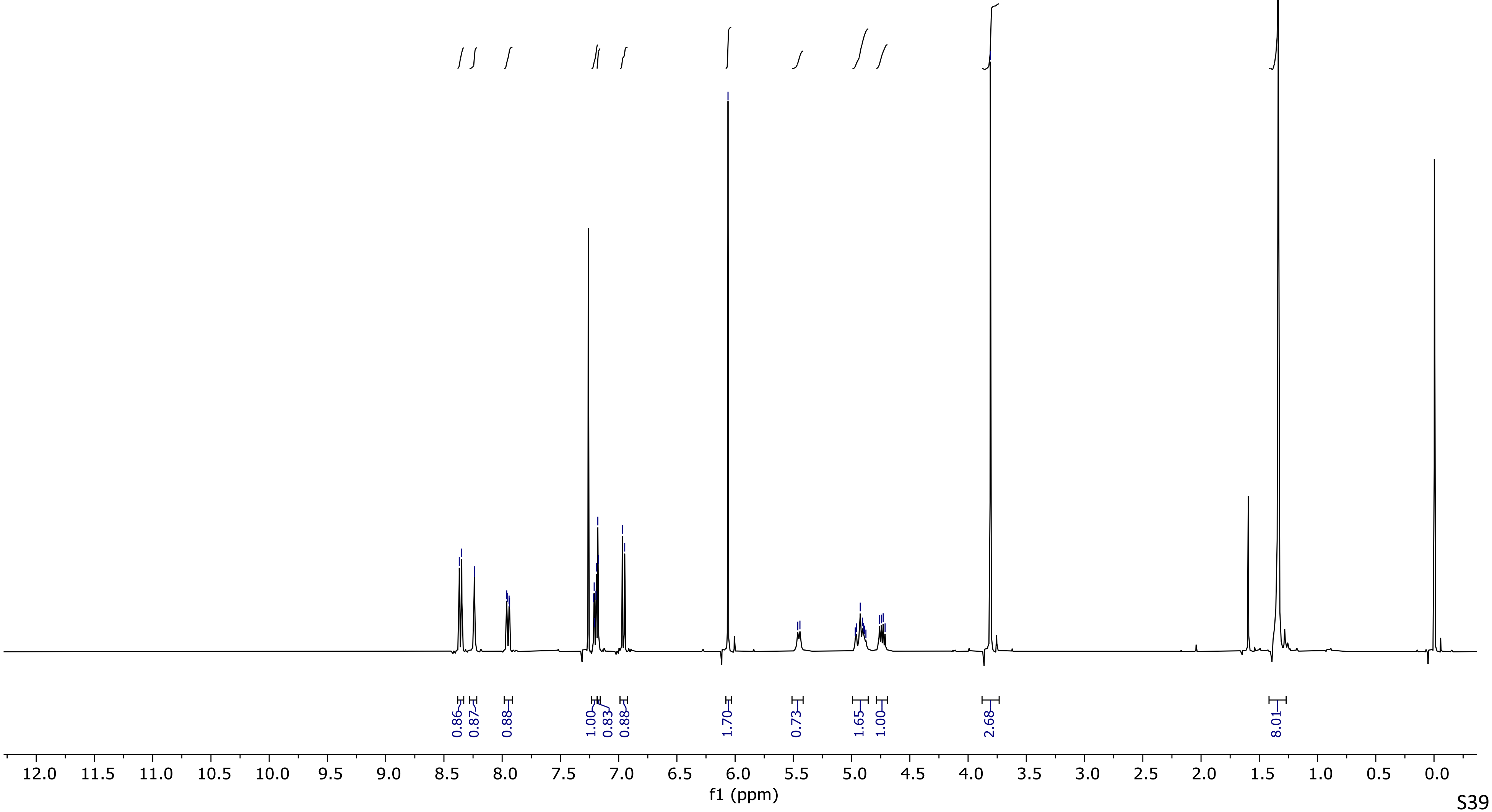

$^{13}C\{^{1}H\}$ NMR (101 MHz, $CDCl_3$)

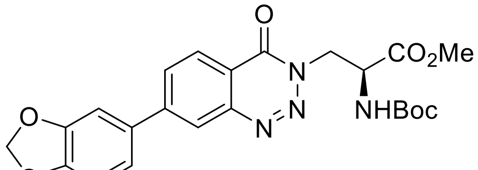

9c

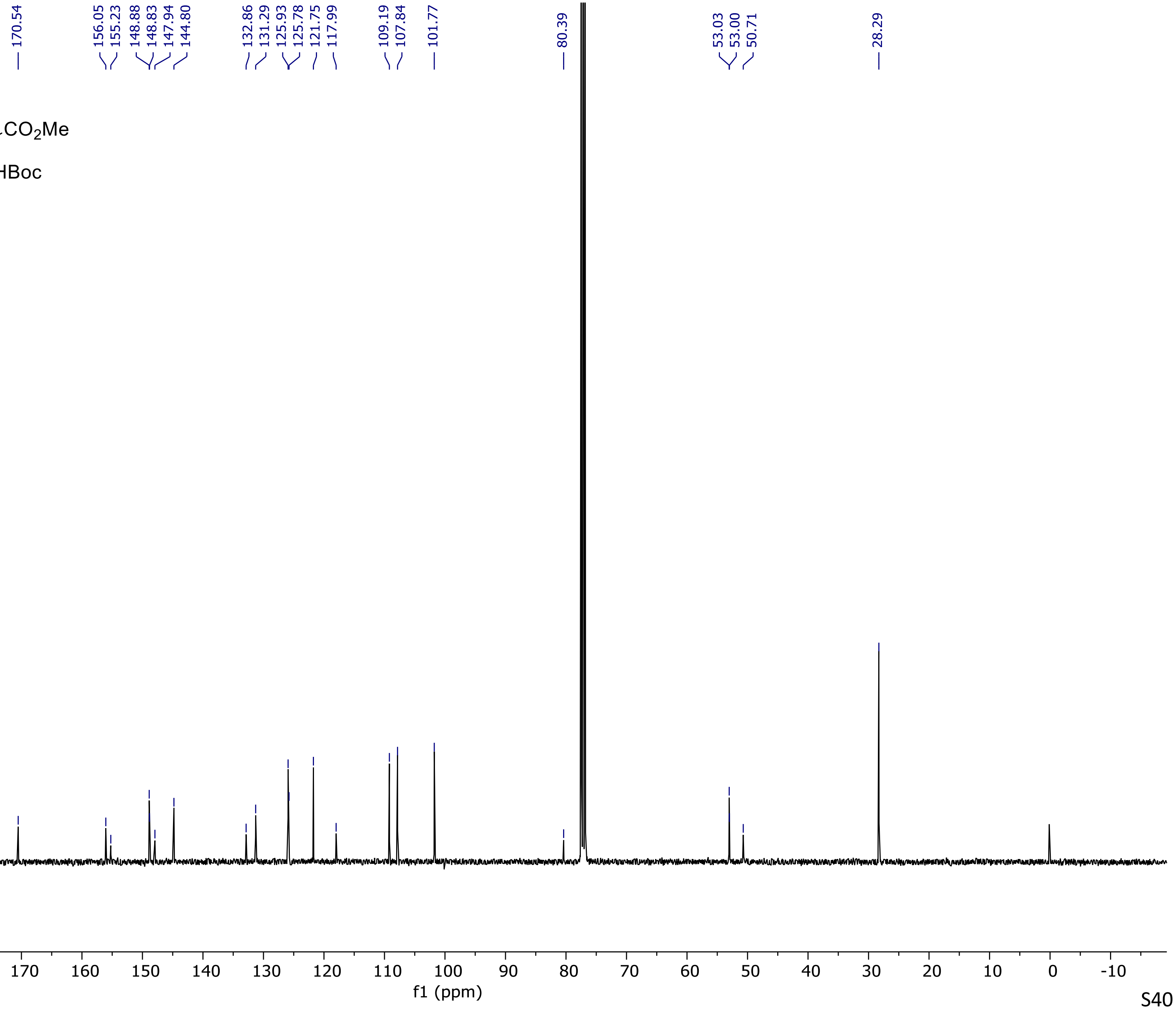

^{1}H NMR (500 MHz, $CDCl_3$)

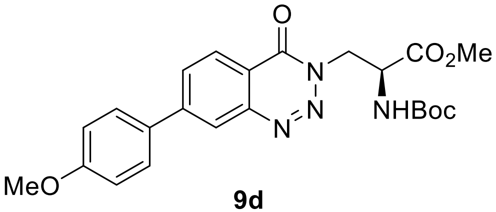

9d

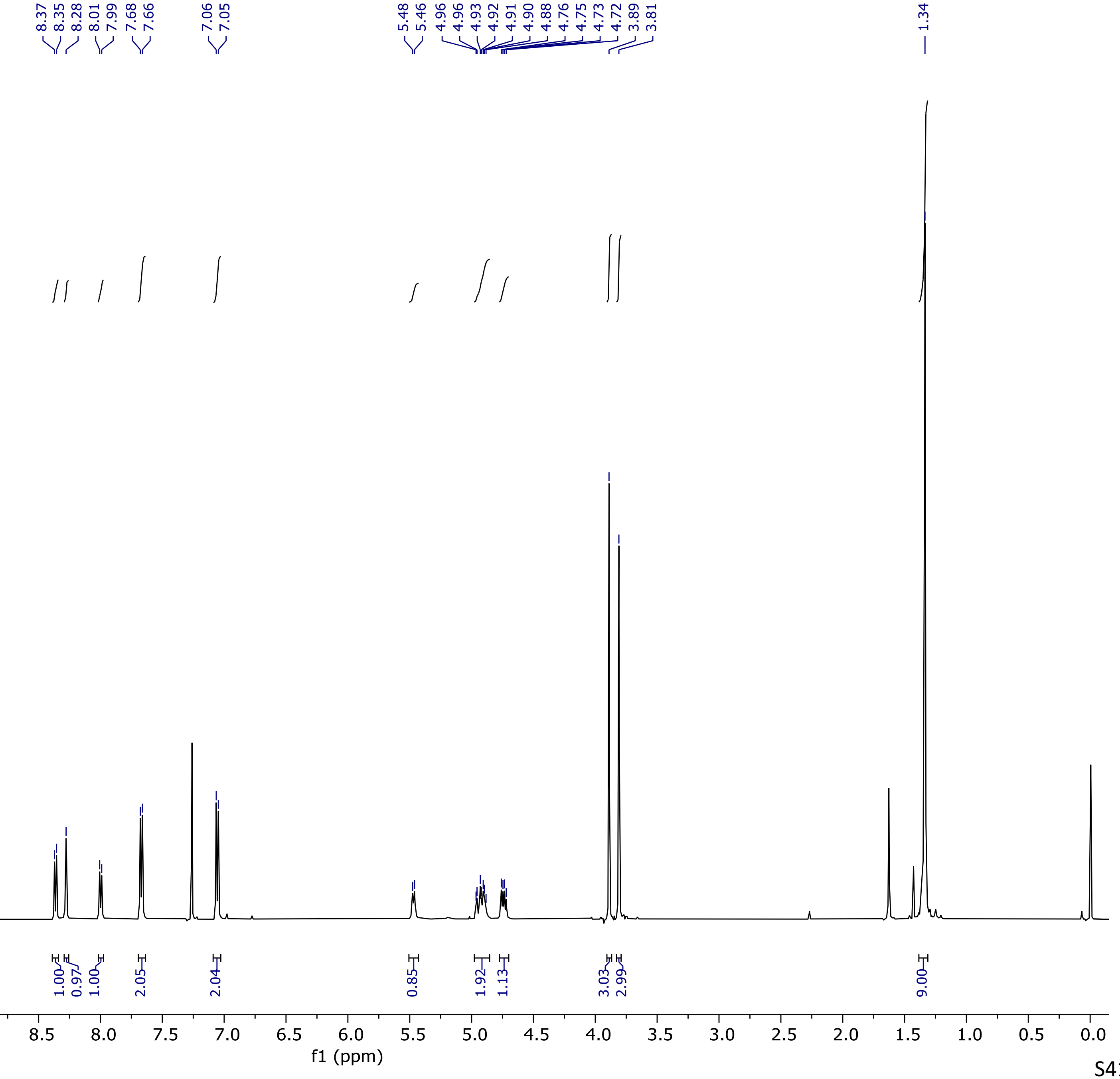

$^{13}C\{^1H\}$ NMR (126 MHz, $CDCl_3$)

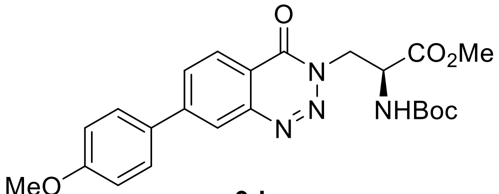

9d

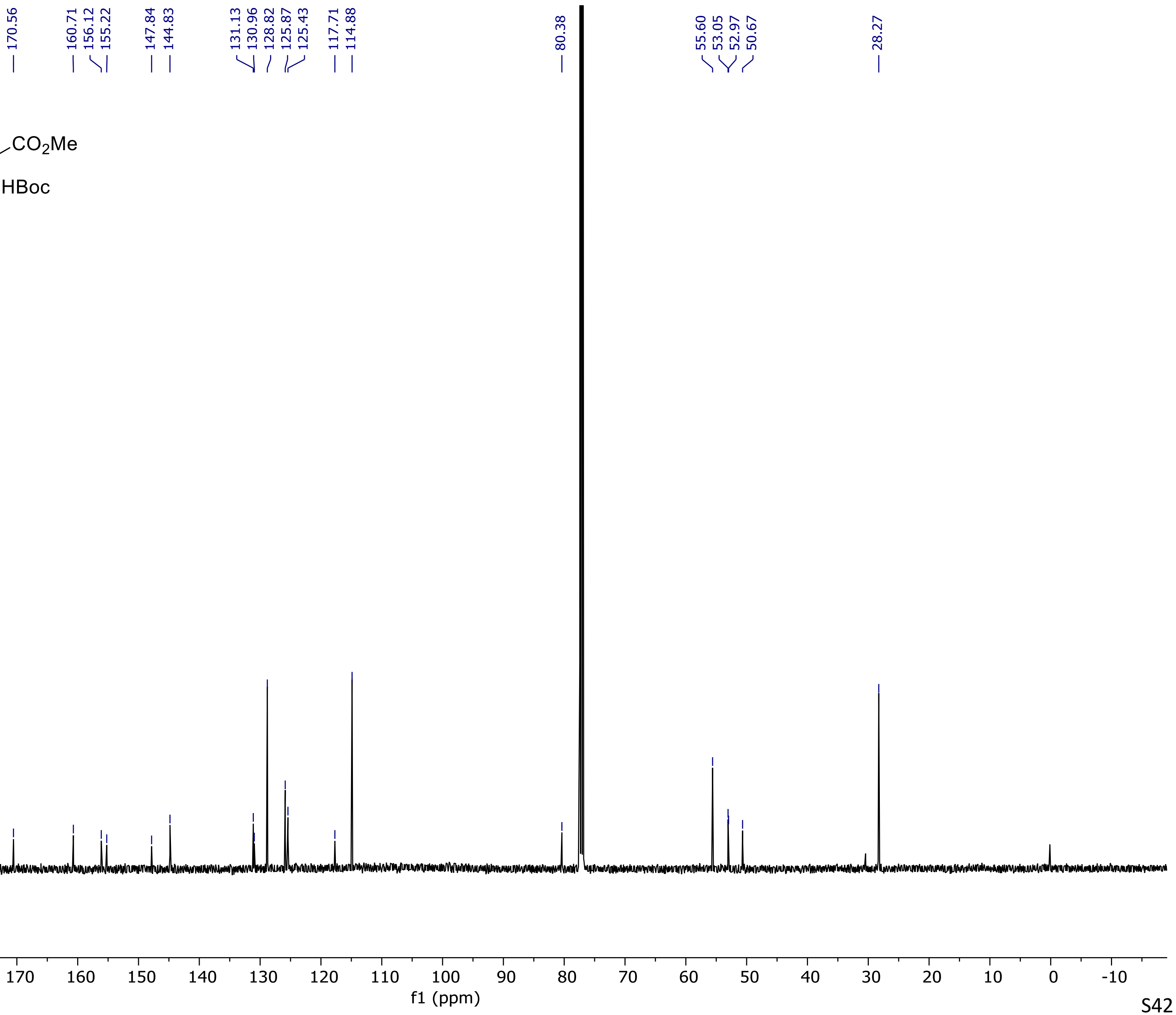

^{1}H NMR (500 MHz, $CDCl_3$)

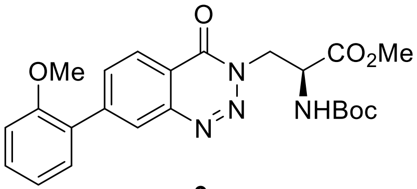

9e

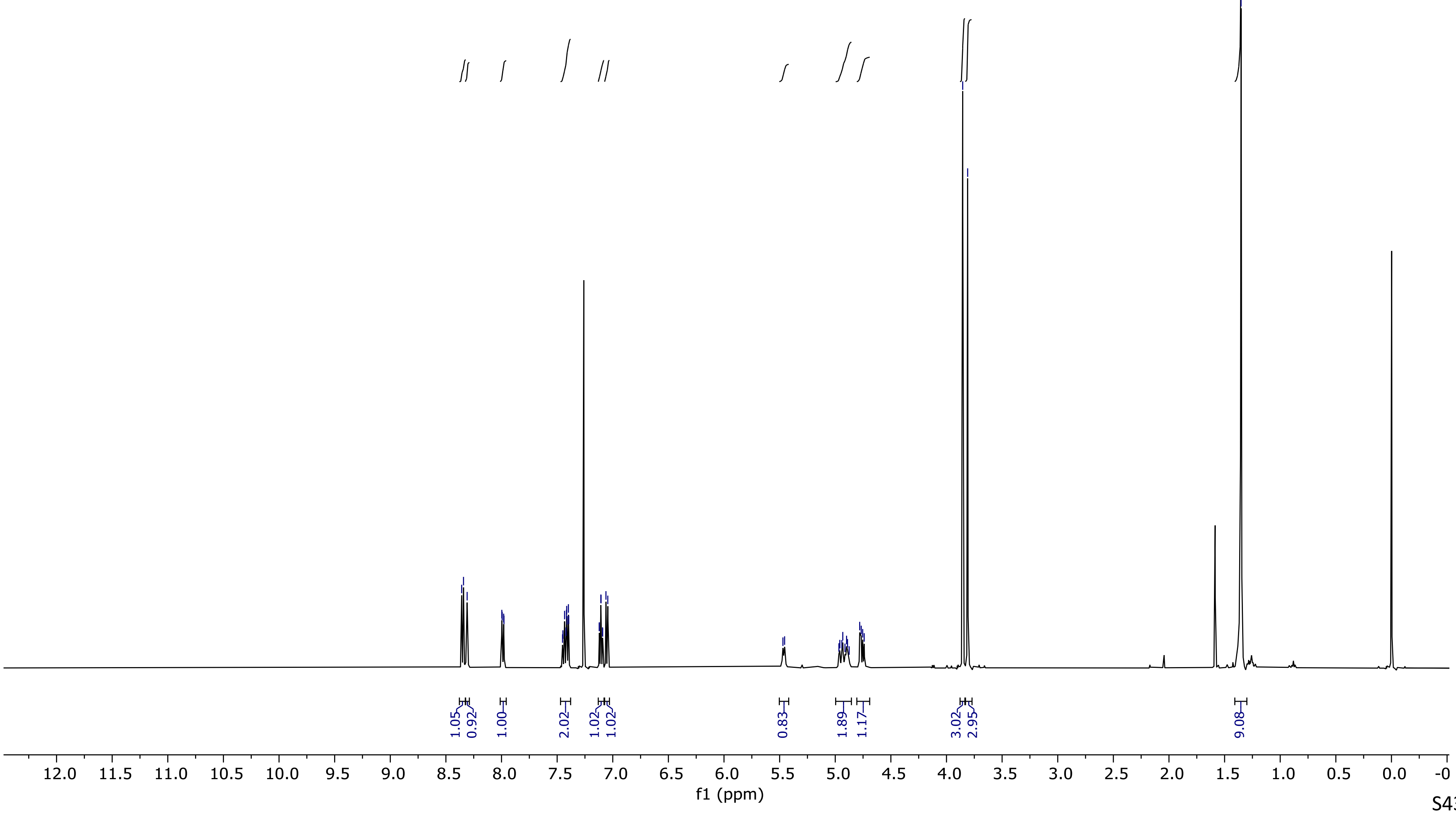

$^{13}C\{^1H\}$ NMR (126 MHz, $CDCl_3$)

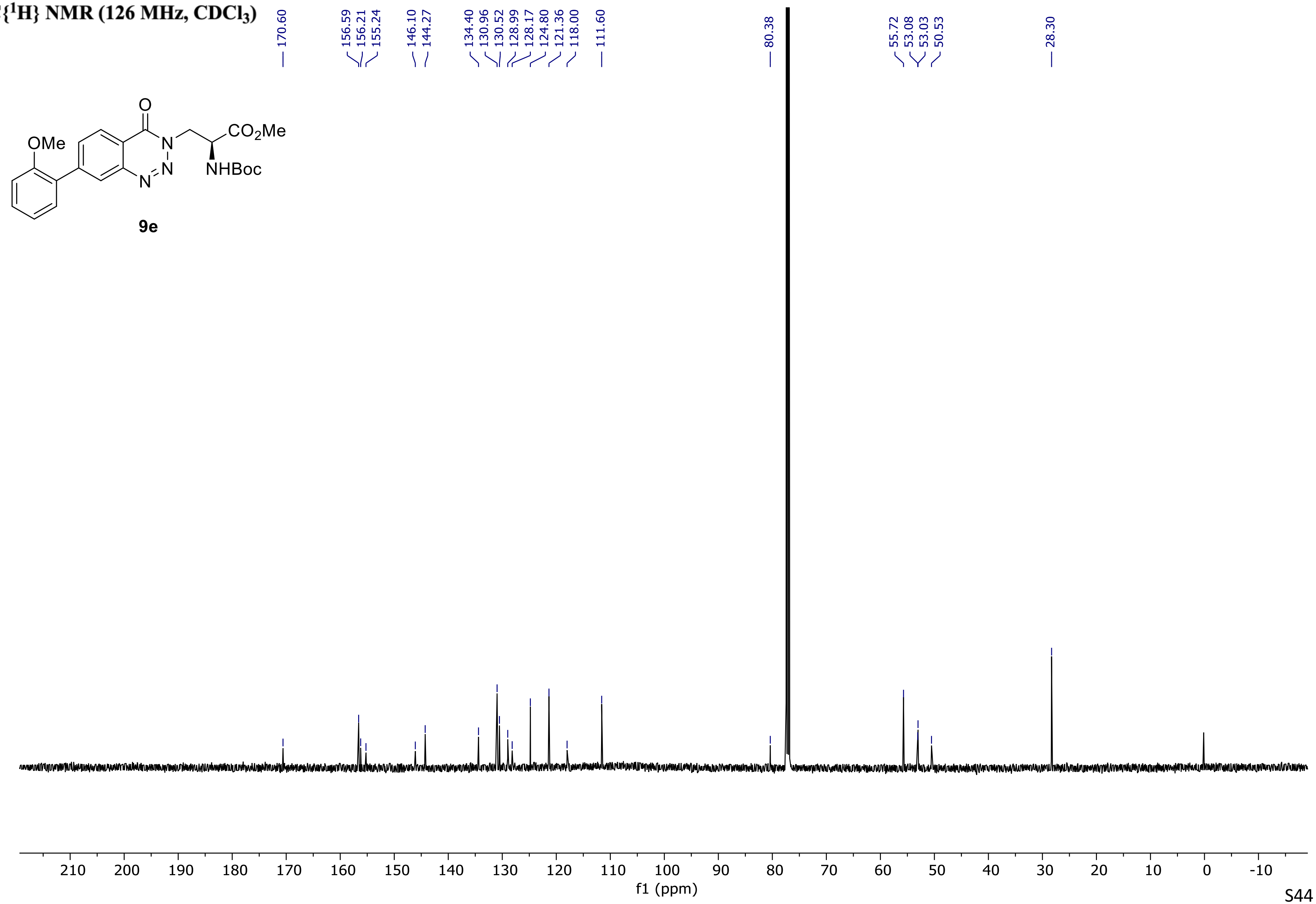

^{1}H NMR (400 MHz, $CDCl_3$)

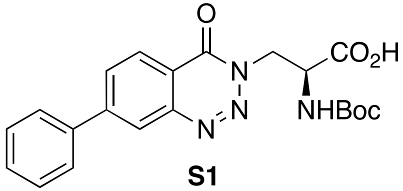

S1

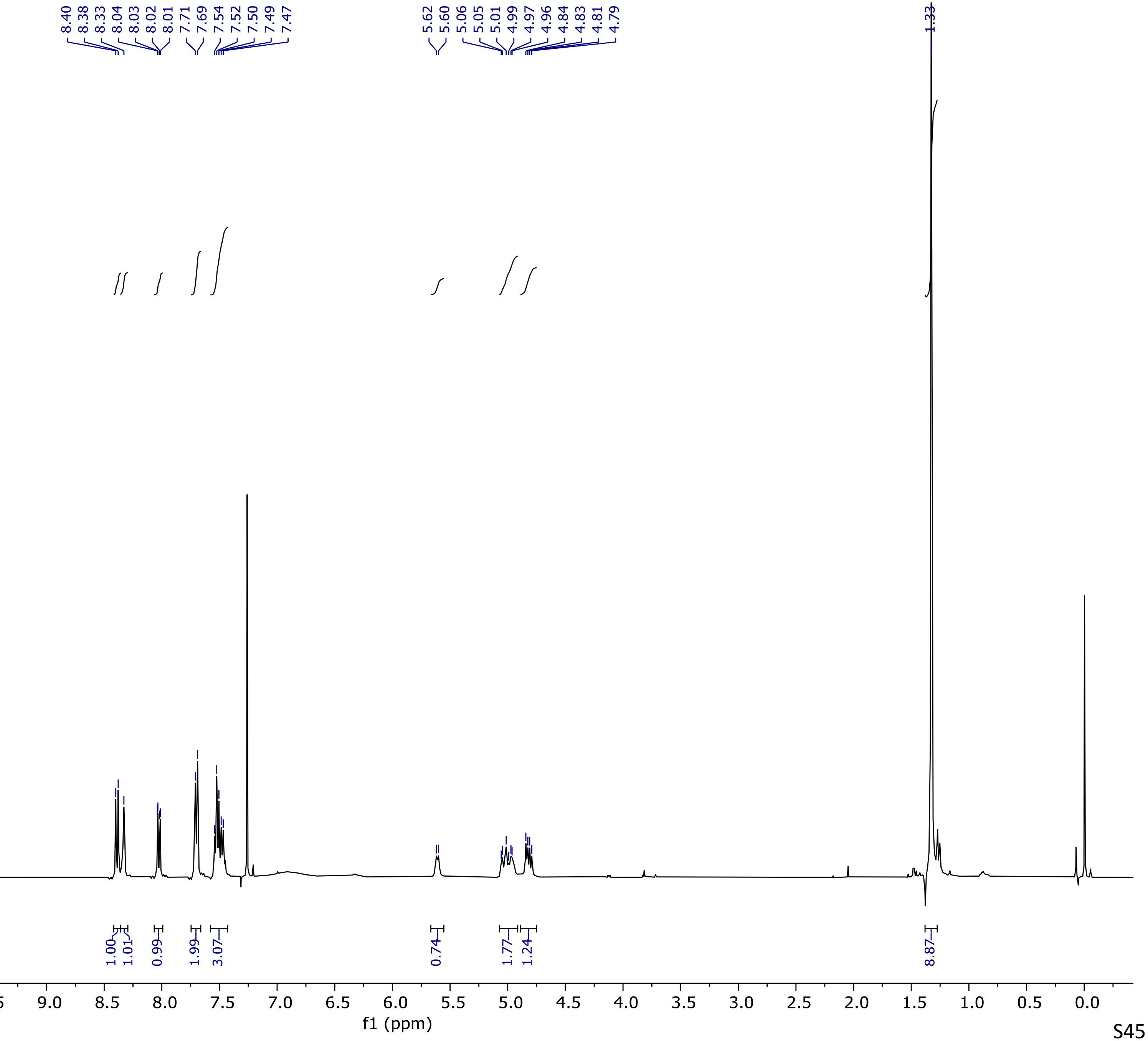

$^{13}C\{^{1}H\}$ NMR (101 MHz, $CDCl_3$)

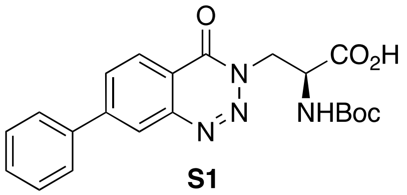

S1

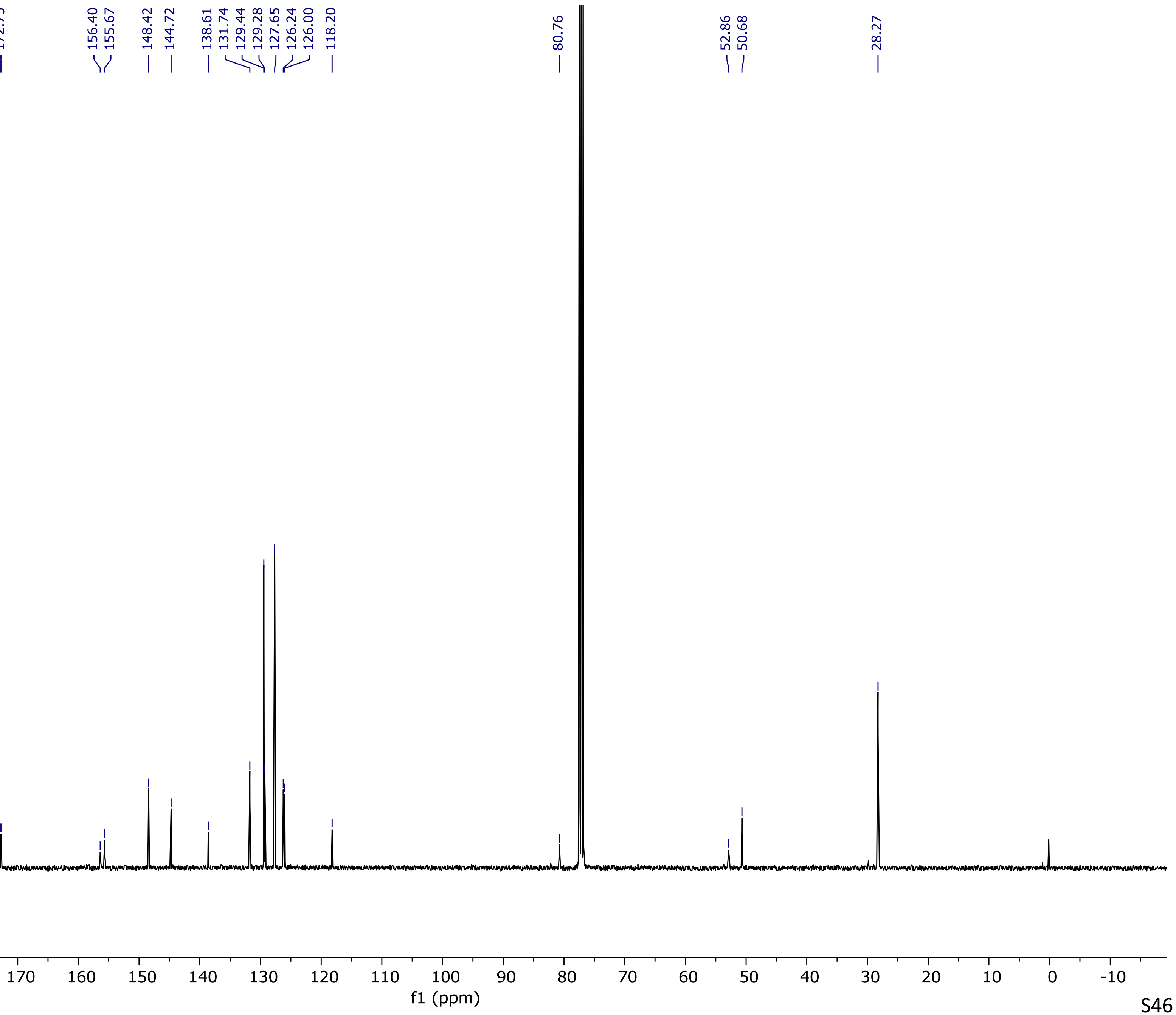

^{1}H NMR (400 MHz, CD_3OD)

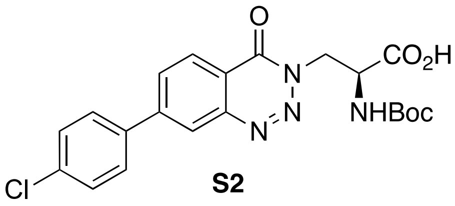

S2

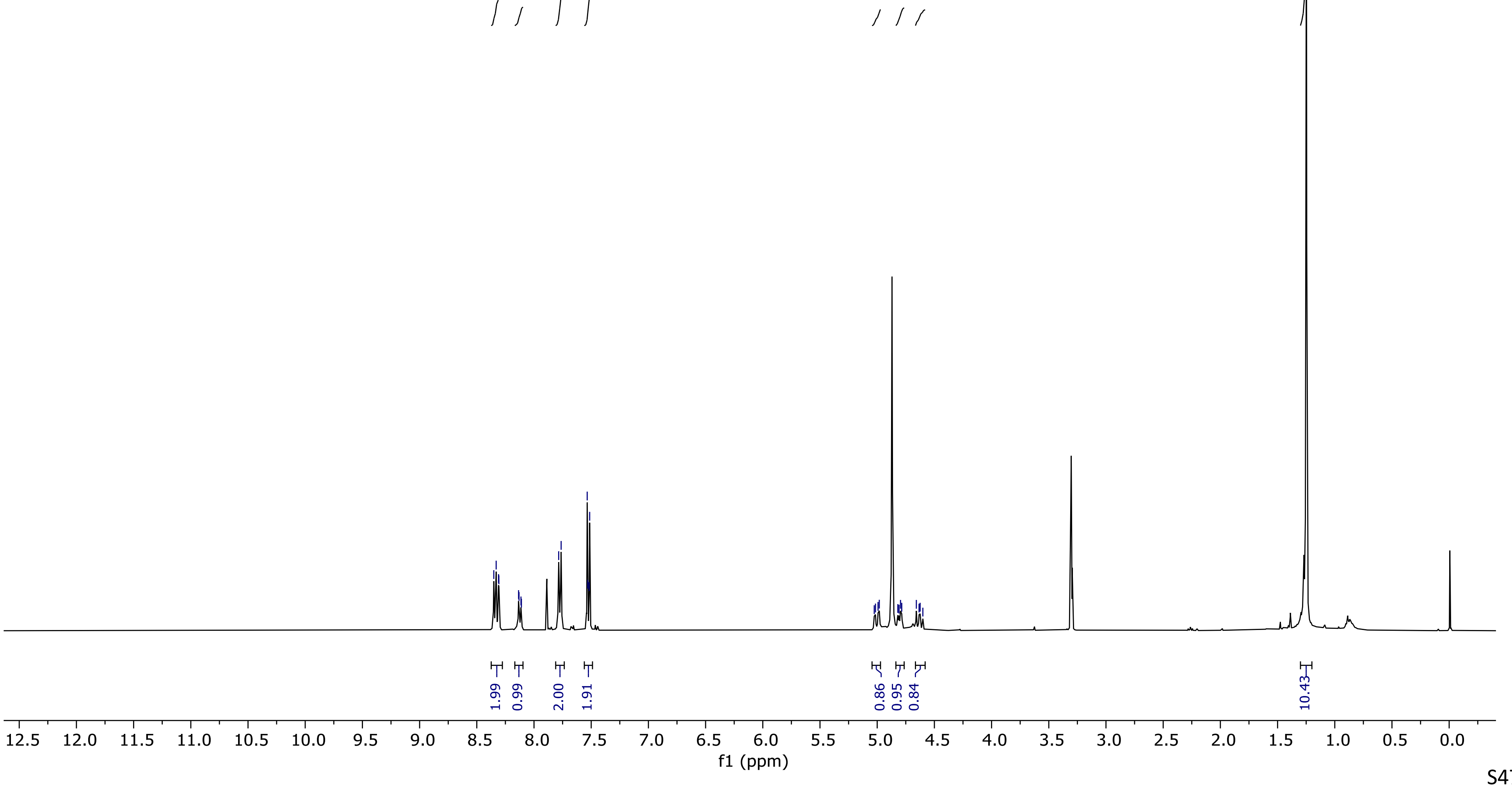

$^{13}C\{^1H\}$ NMR (101 MHz, CD_3OD)

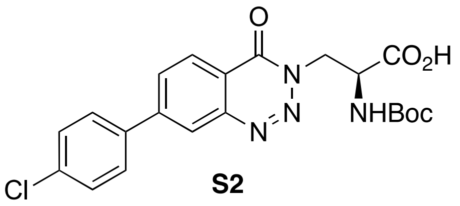

S2

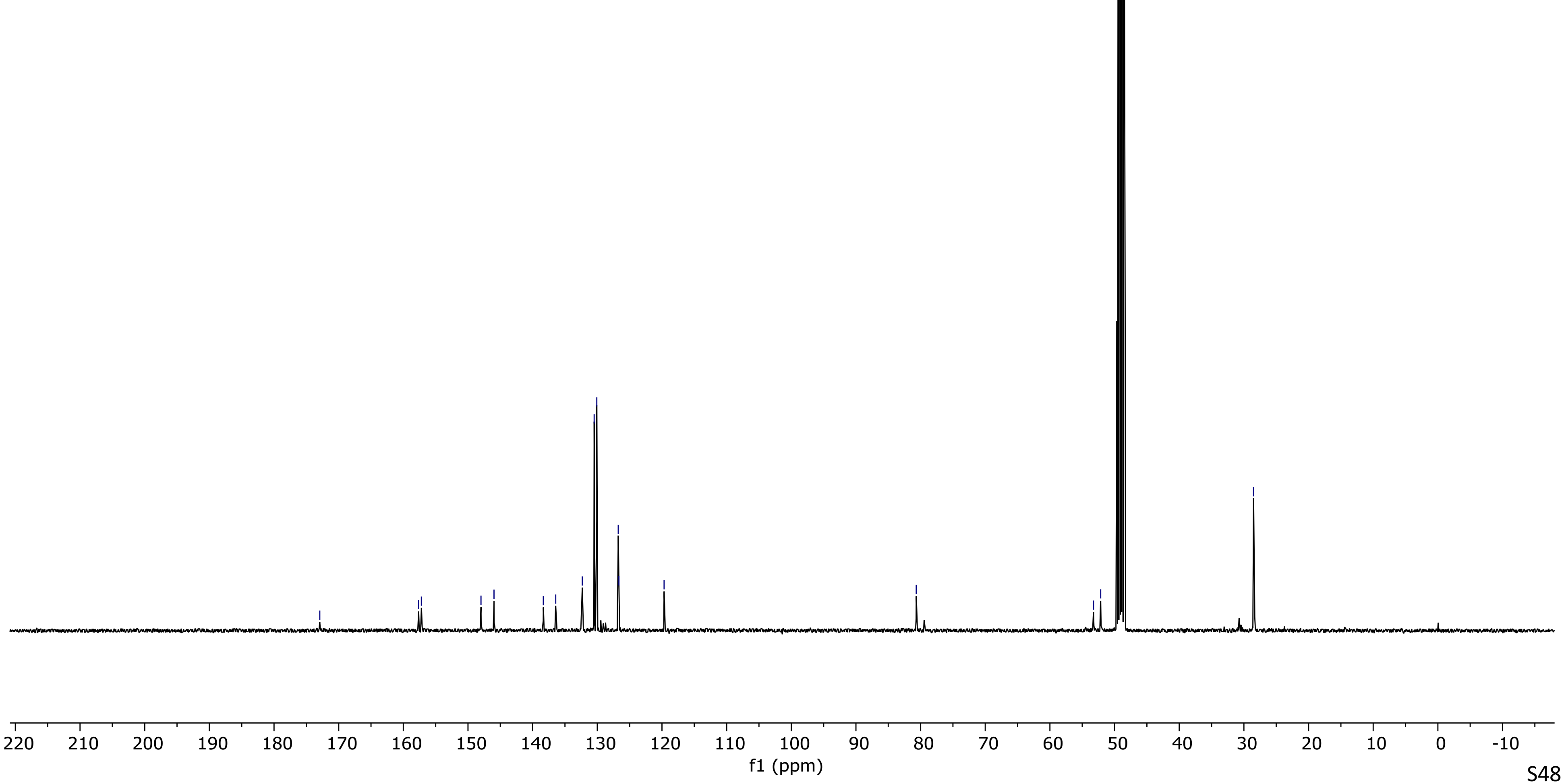

^{1}H NMR (400 MHz, DMSO-d_6)

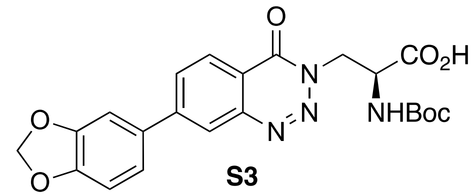

S3

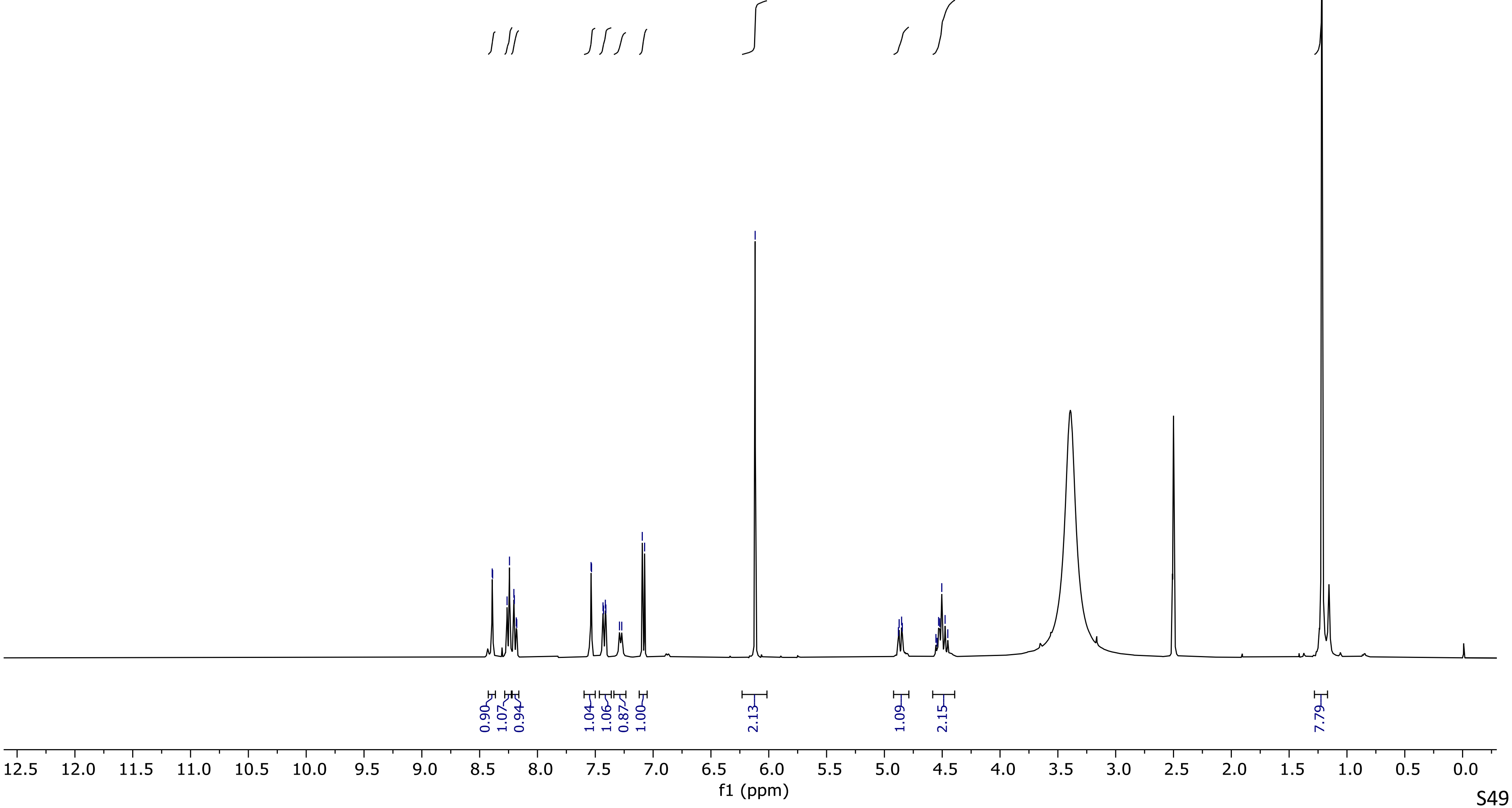

$^{13}C\{^{1}H\}$ NMR (101 MHz, DMSO-d_6)

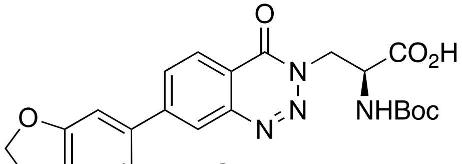

S3

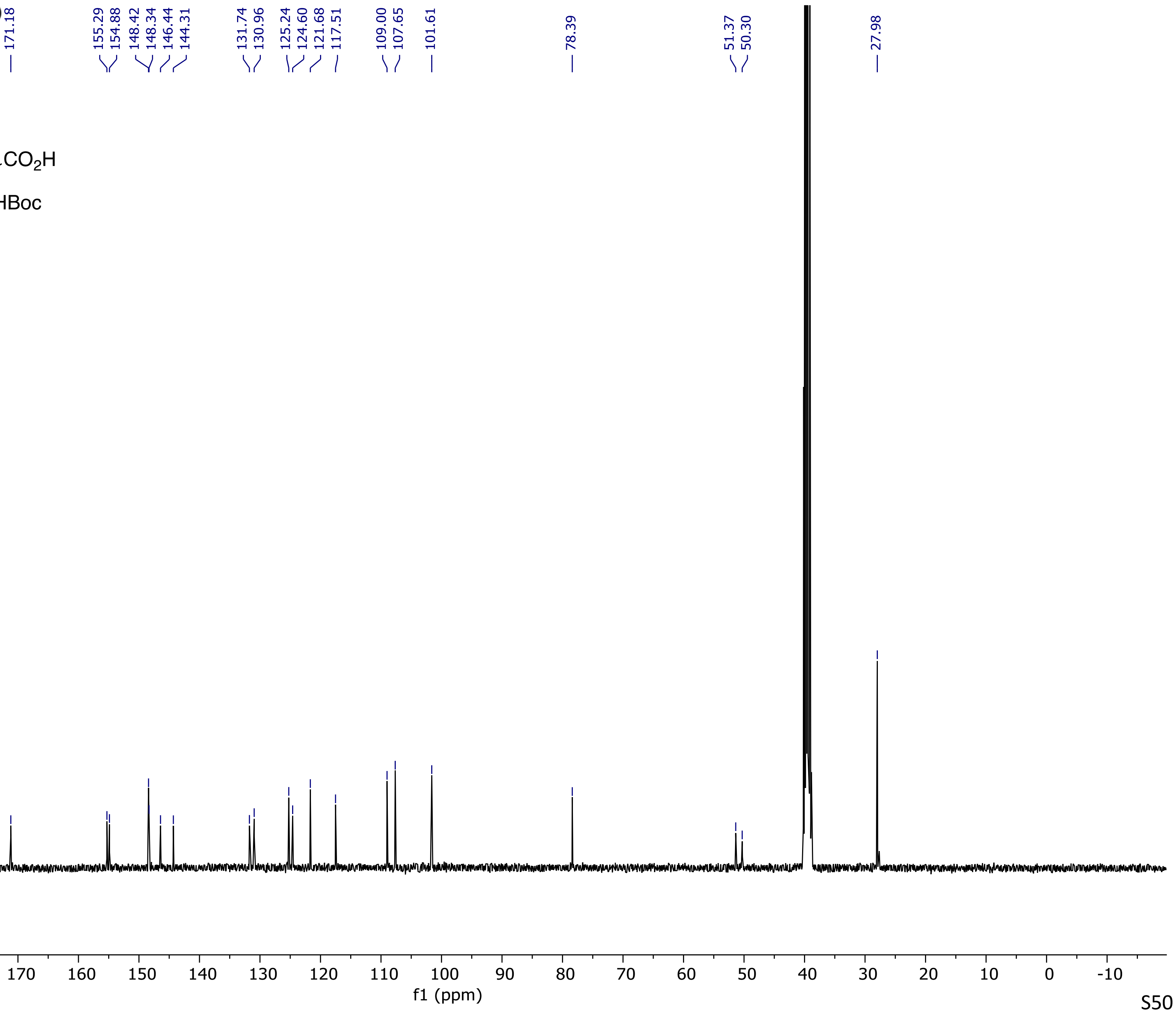

^{1}H NMR (400 MHz, $CDCl_3$)

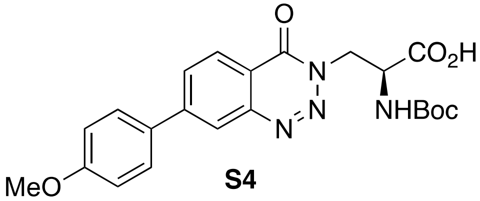

S4

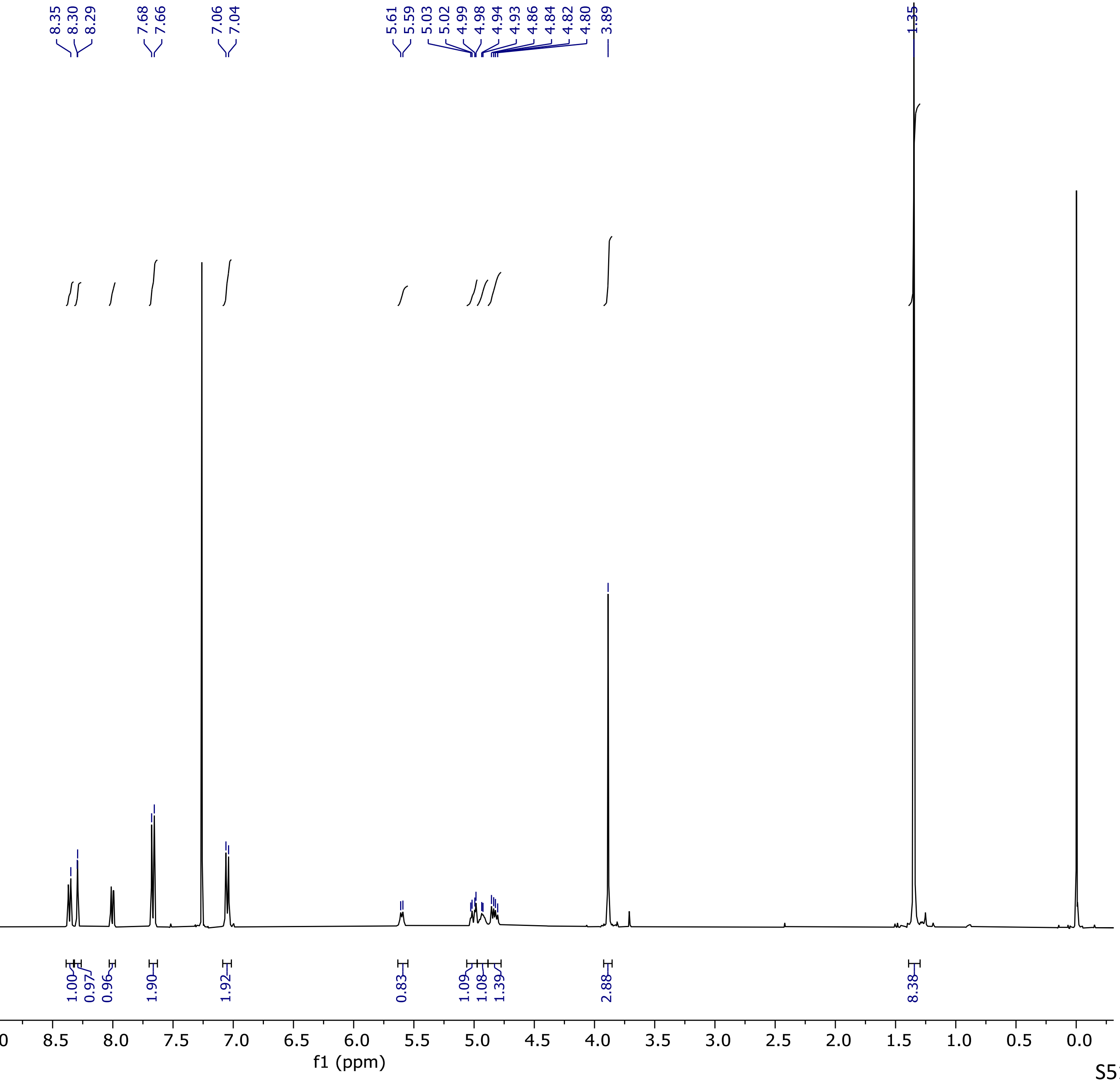

$^{13}C\{^1H\}$ NMR (101 MHz, $CDCl_3$)

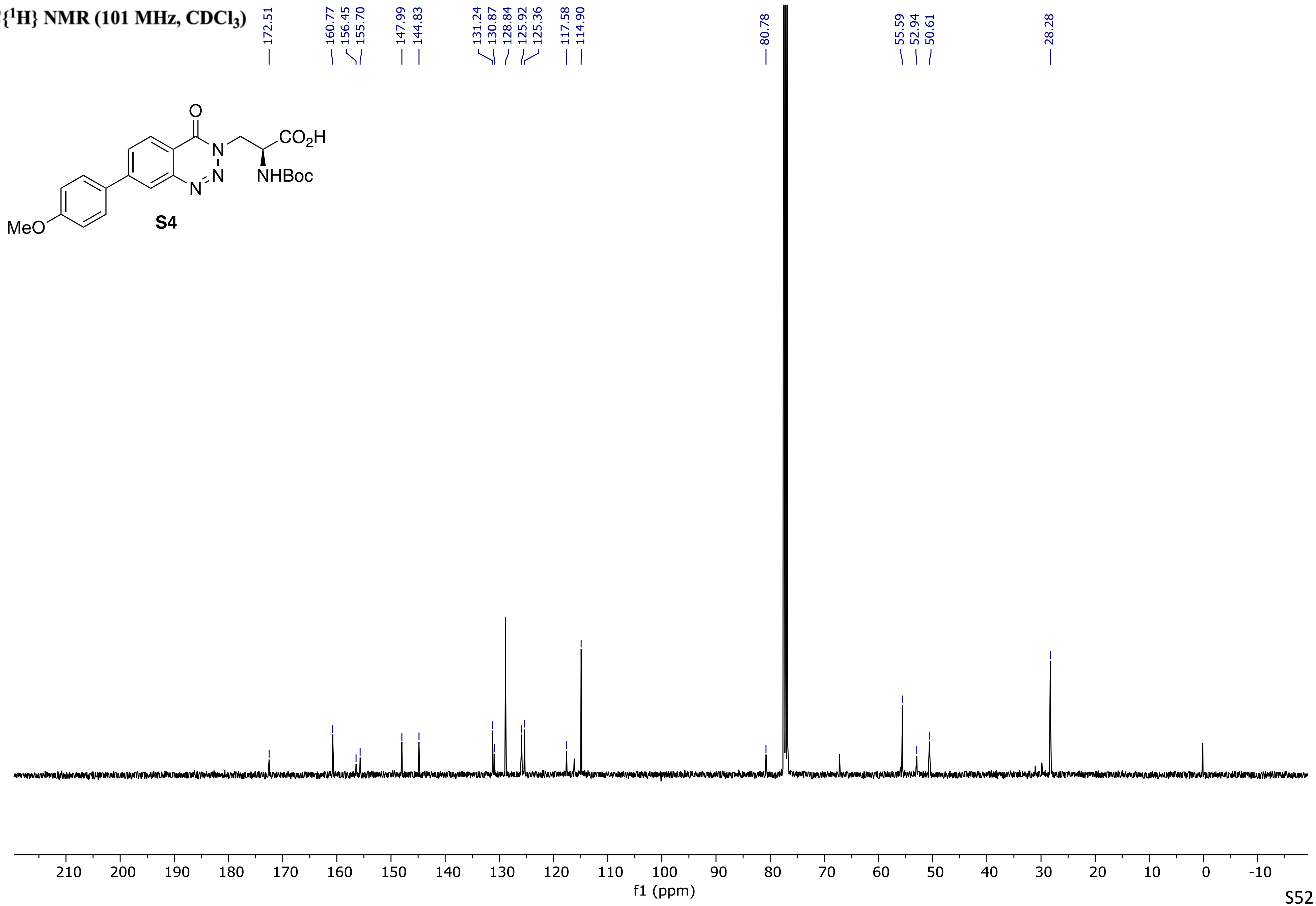

^{1}H NMR (400 MHz, CDCl$_3$)

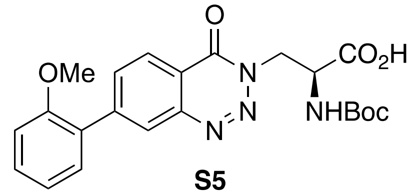

S5

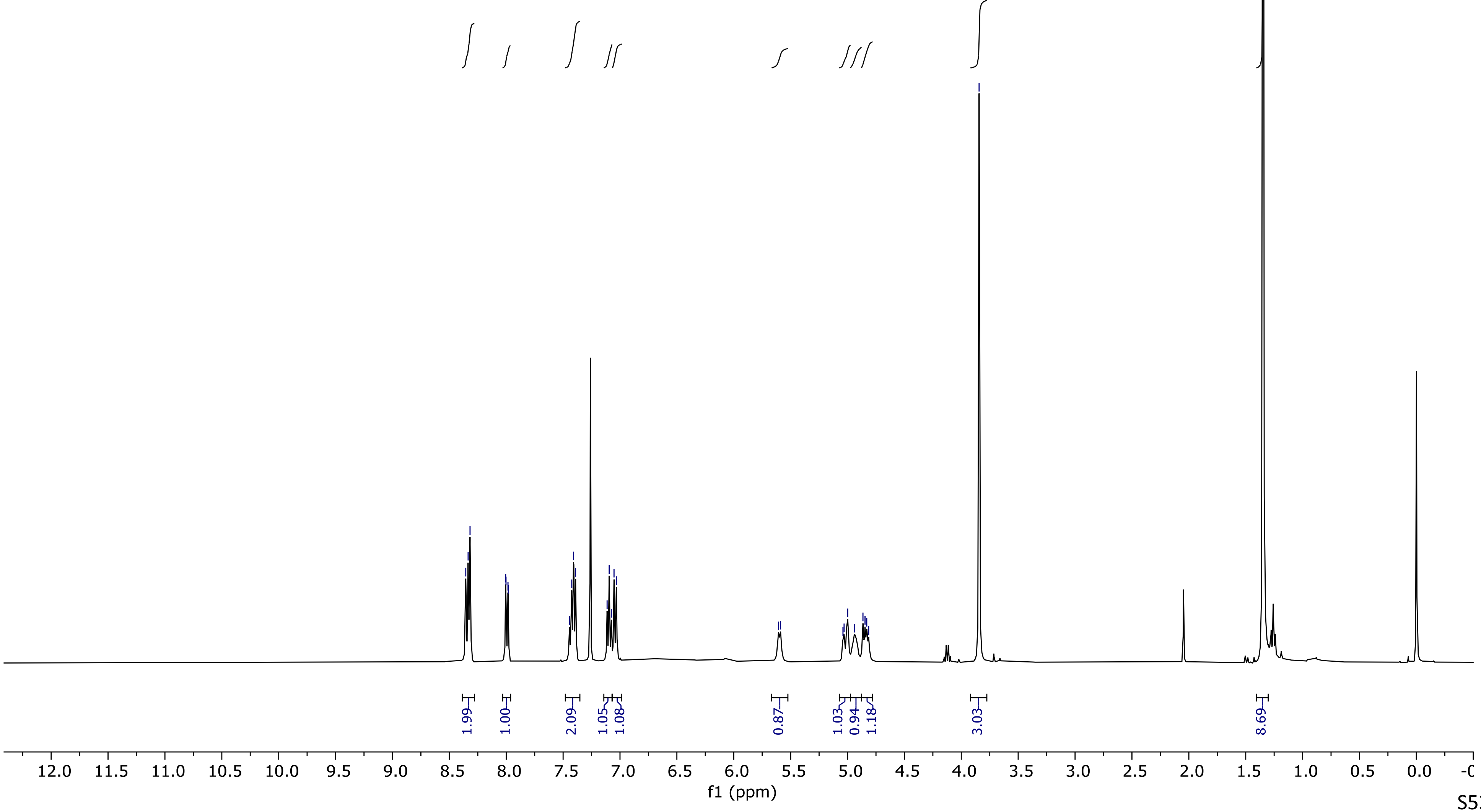

$^{13}C\{^1H\}$ NMR (101 MHz, $CDCl_3$)

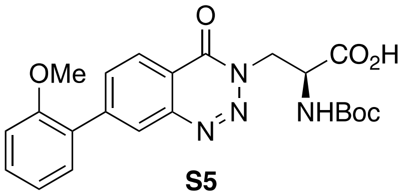

S5

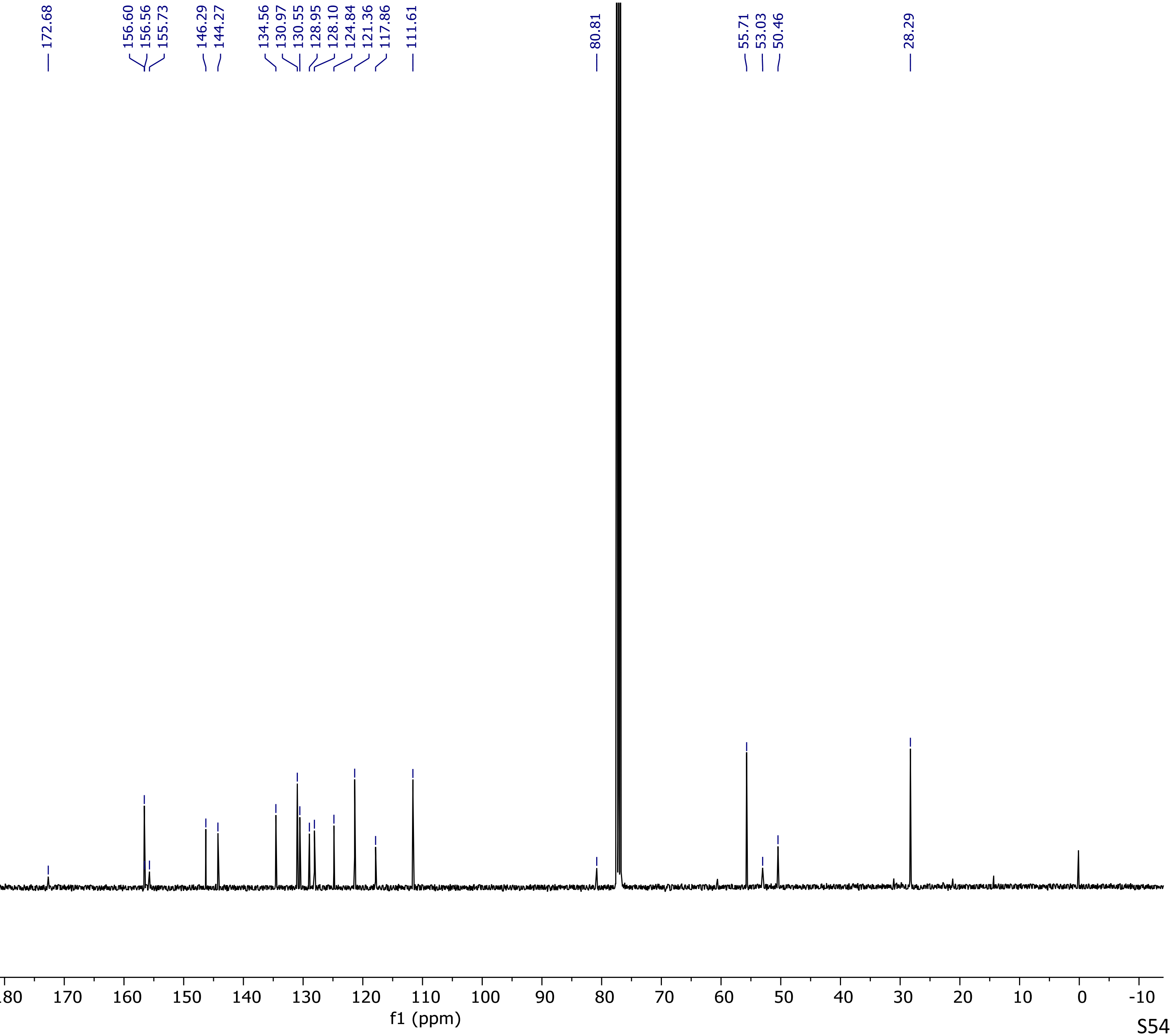

^{1}H NMR (400 MHz, DMSO-d_6)

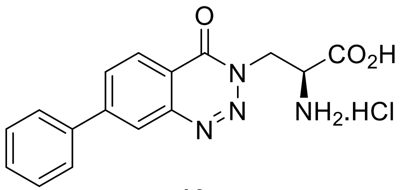

10a

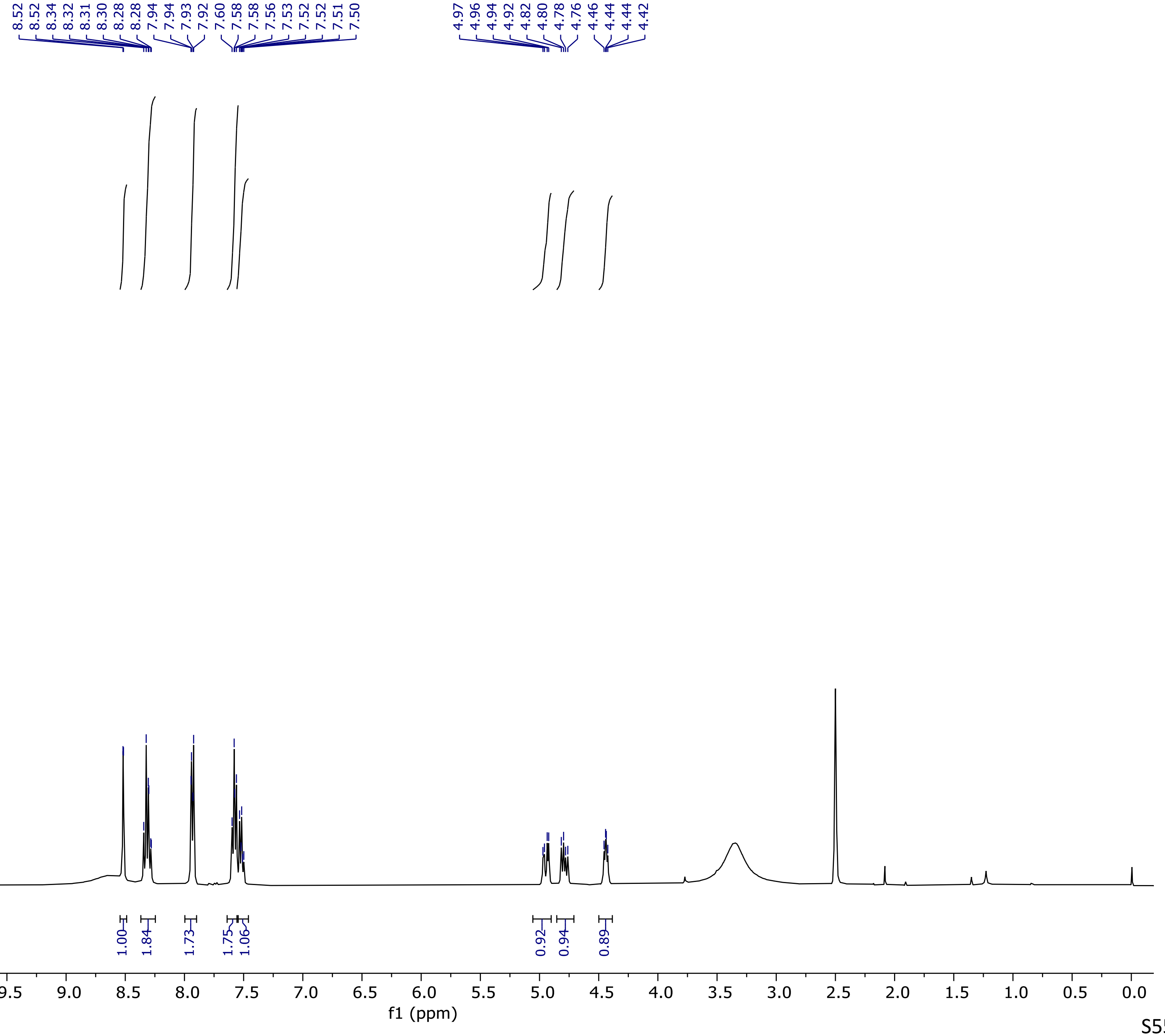

$^{13}C\{^1H\}$ NMR (101 MHz, DMSO-d_6)

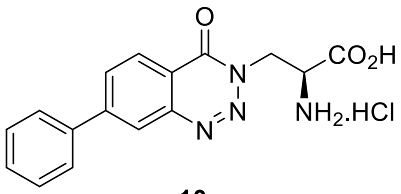

10a

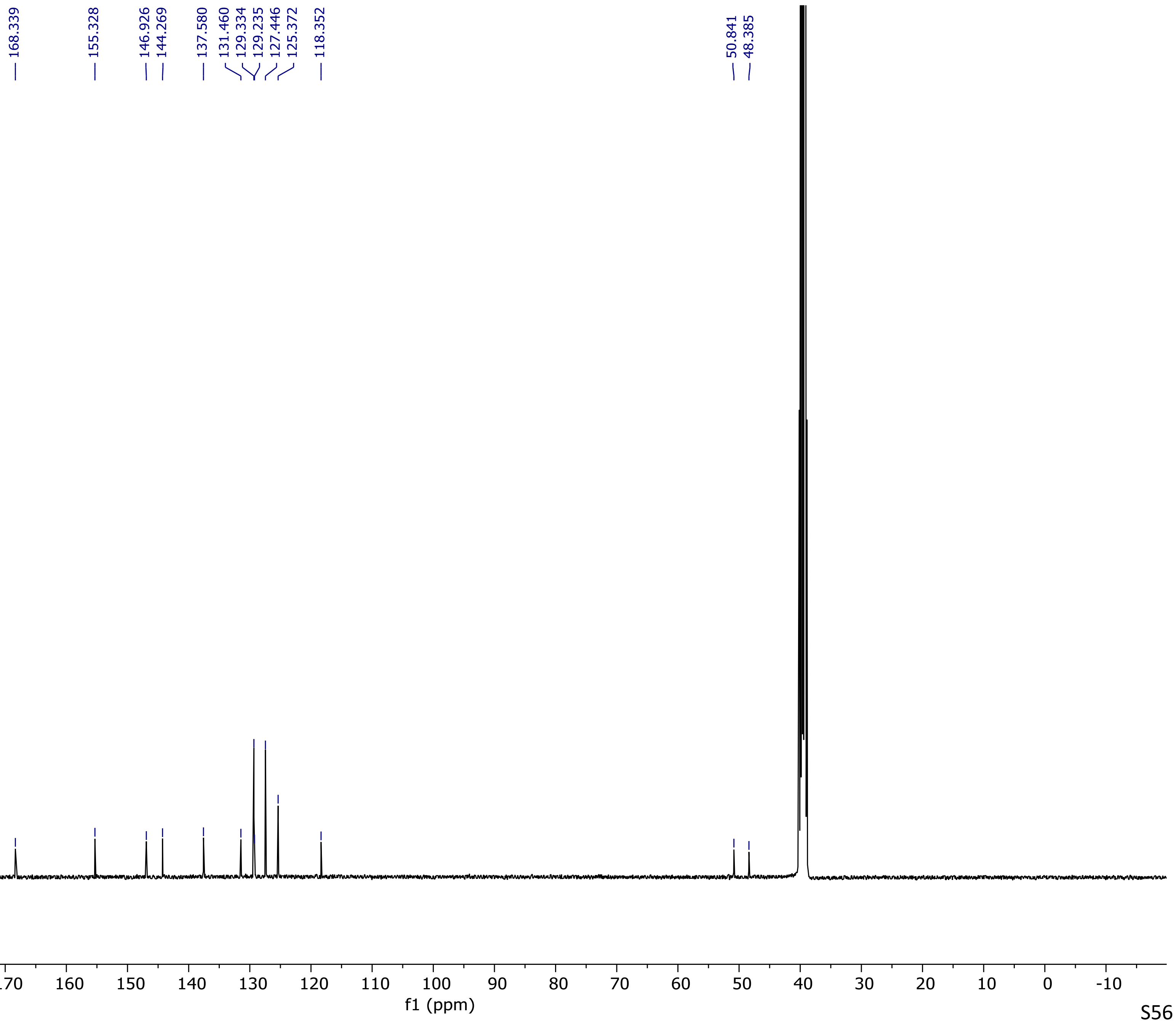

^{1}H NMR (400 MHz, DMSO-d_6)

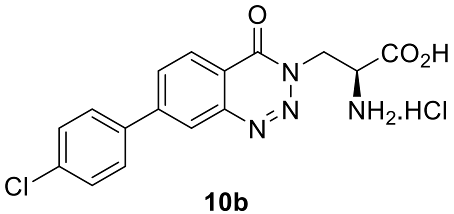

10b

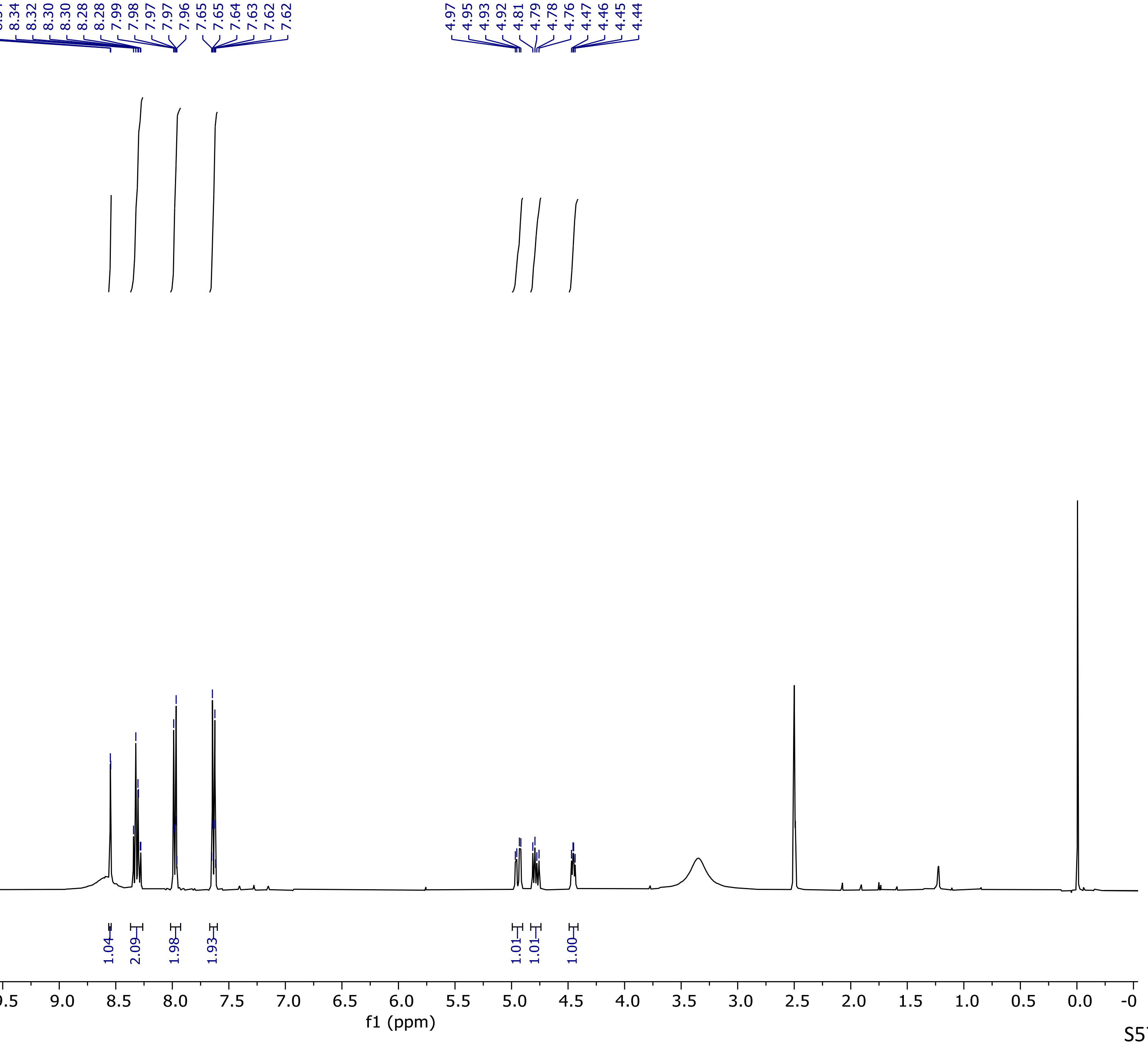

$^{13}C\{^{1}H\}$ NMR (101 MHz, DMSO-d_6)

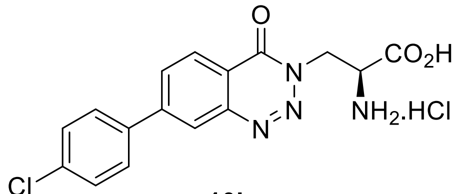

10b

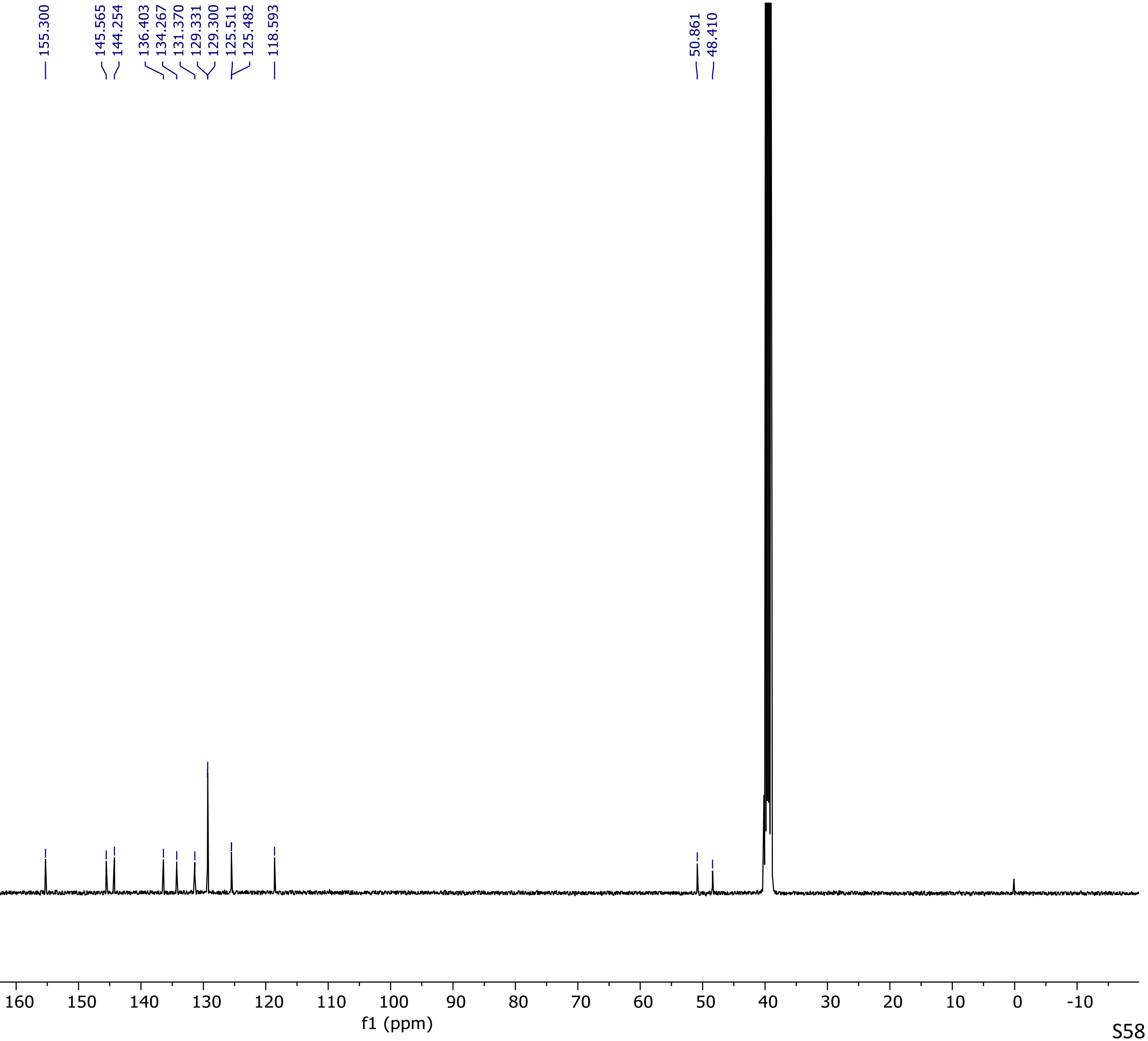

^{1}H NMR (400 MHz, DMSO-d_6)

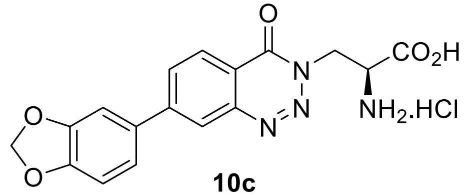

10c

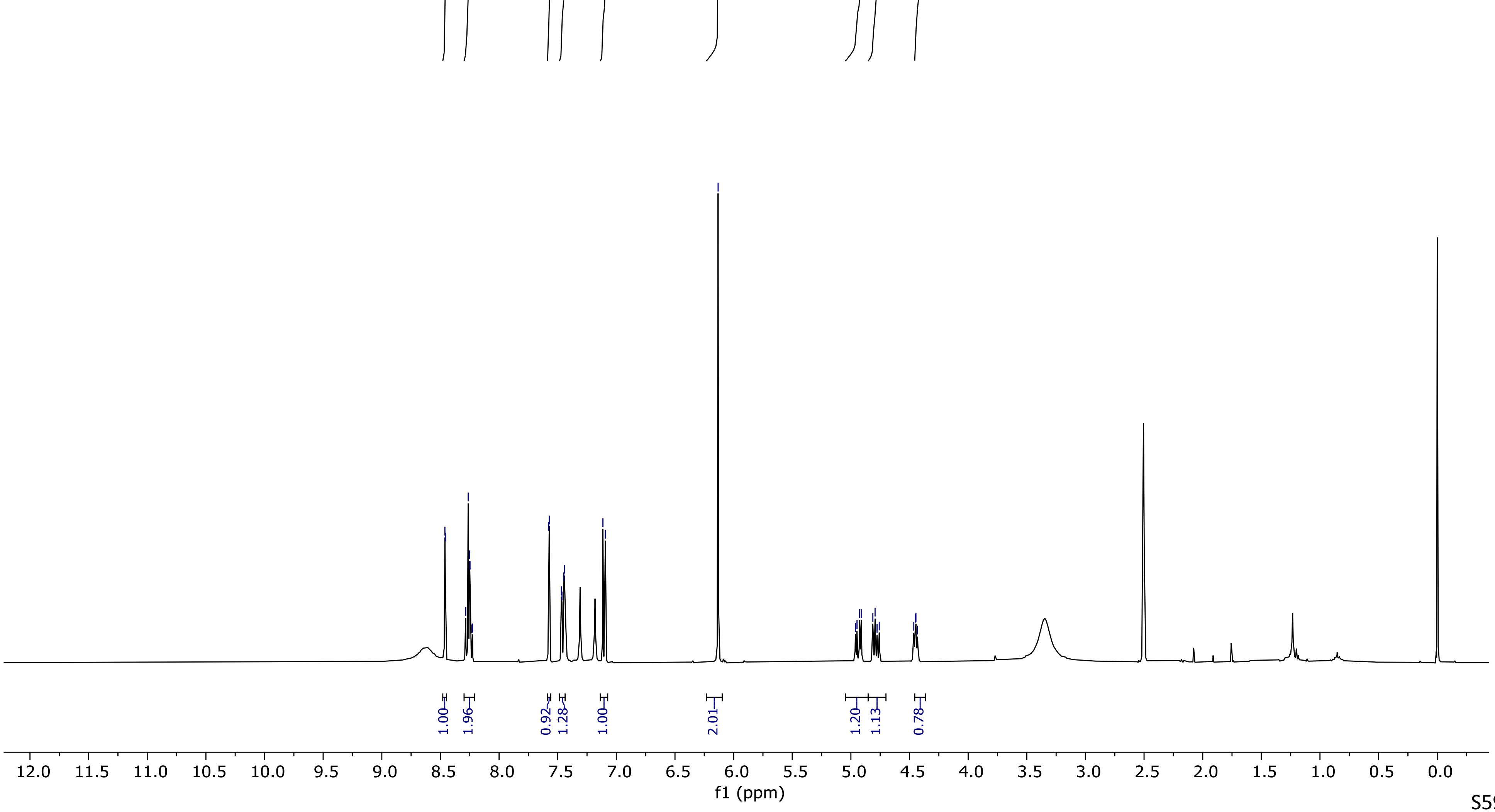

$^{13}C\{^{1}H\}$ NMR (101 MHz, DMSO-d_6)

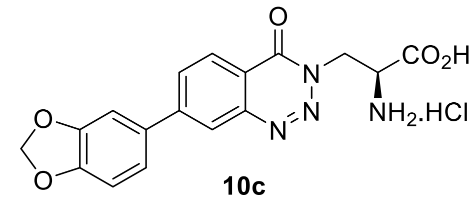

10c

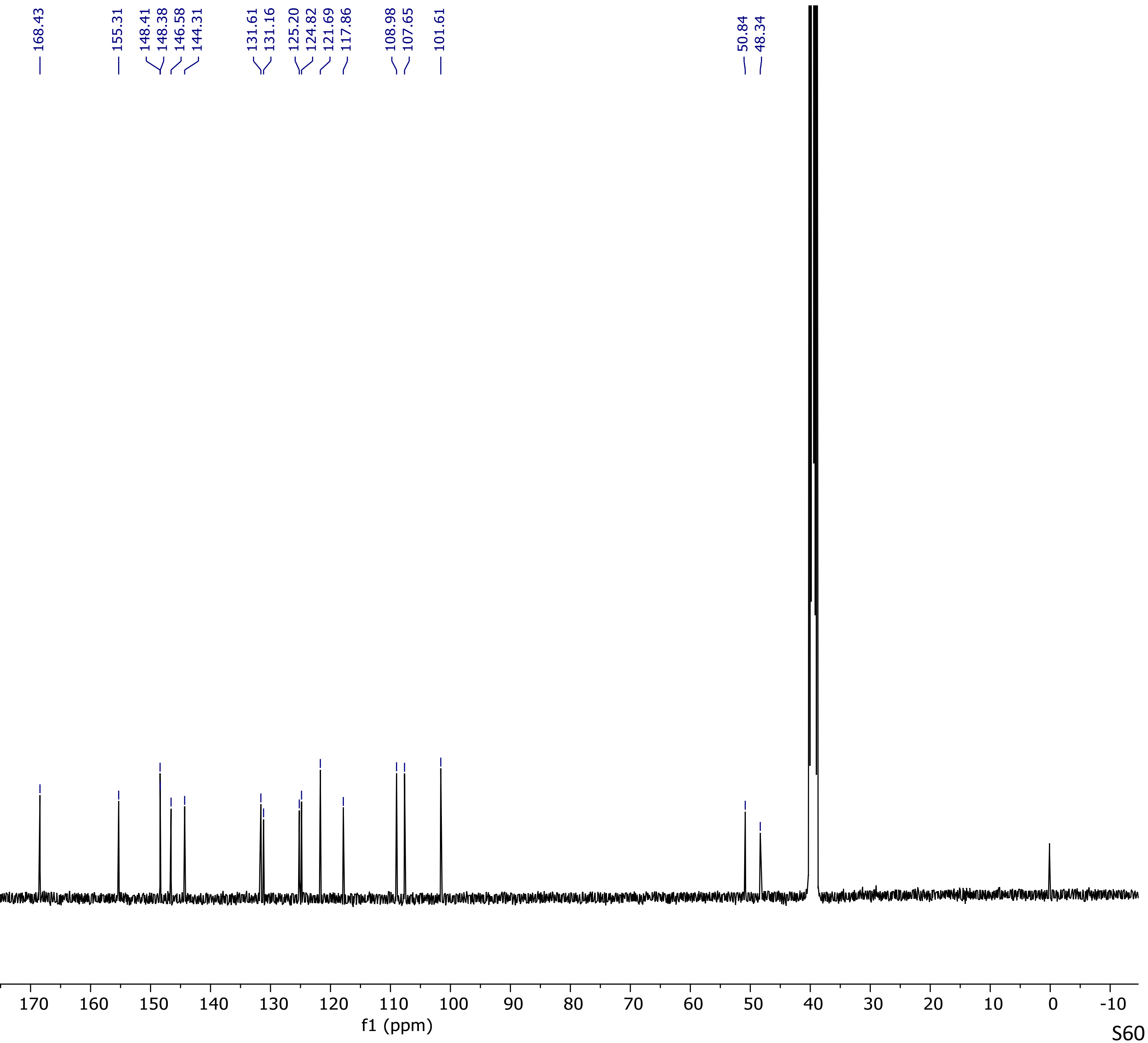

^{1}H NMR (400 MHz, DMSO-d_6)

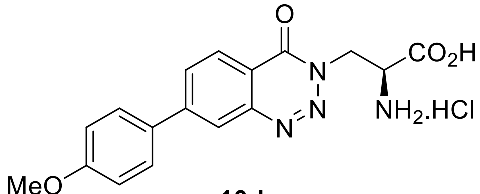

10d

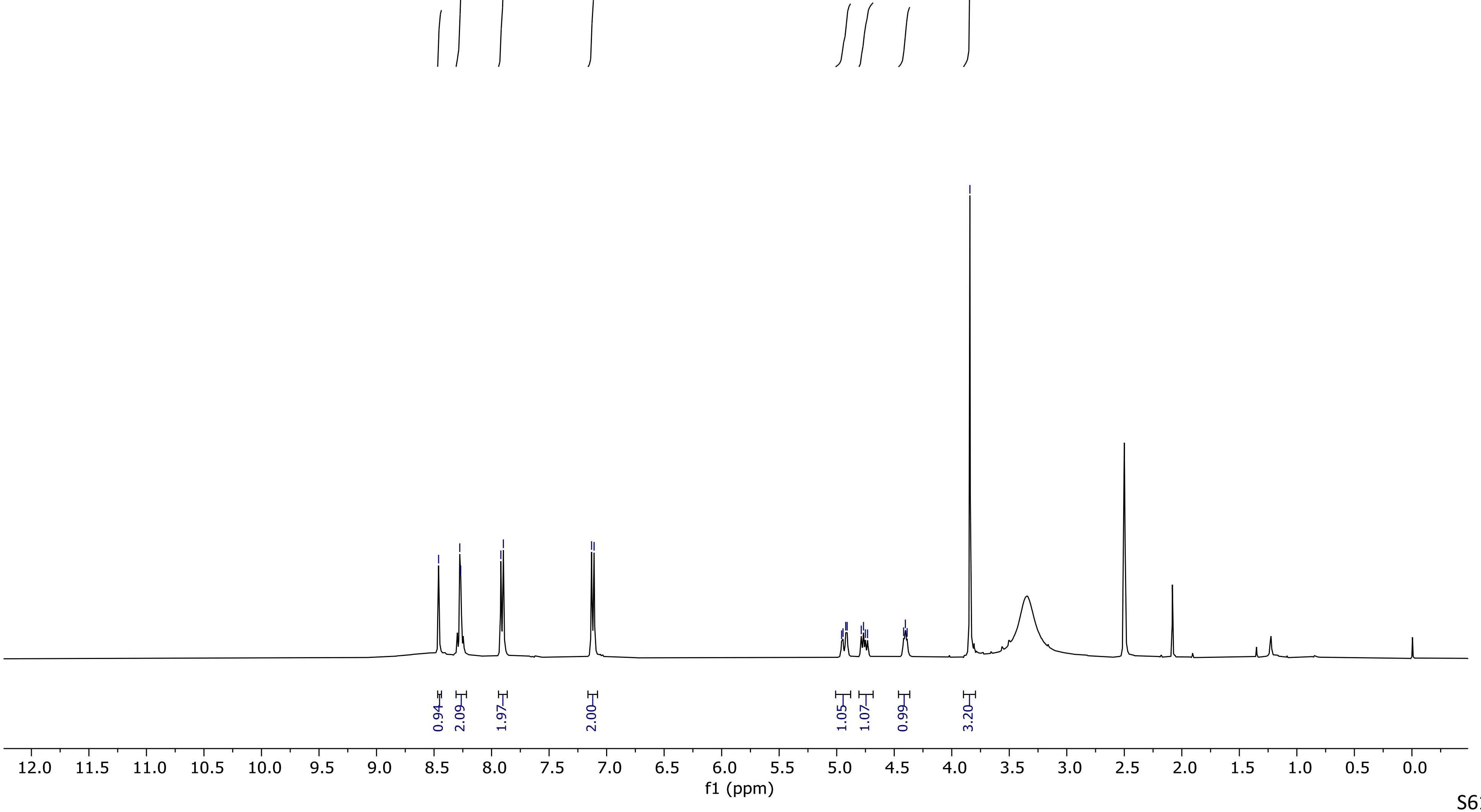

$^{13}C\{^{1}H\}$ NMR (101 MHz, DMSO-d_6)

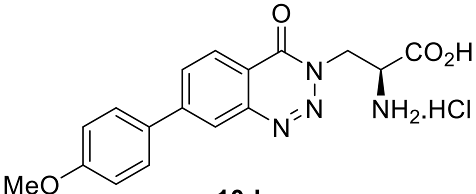

10d

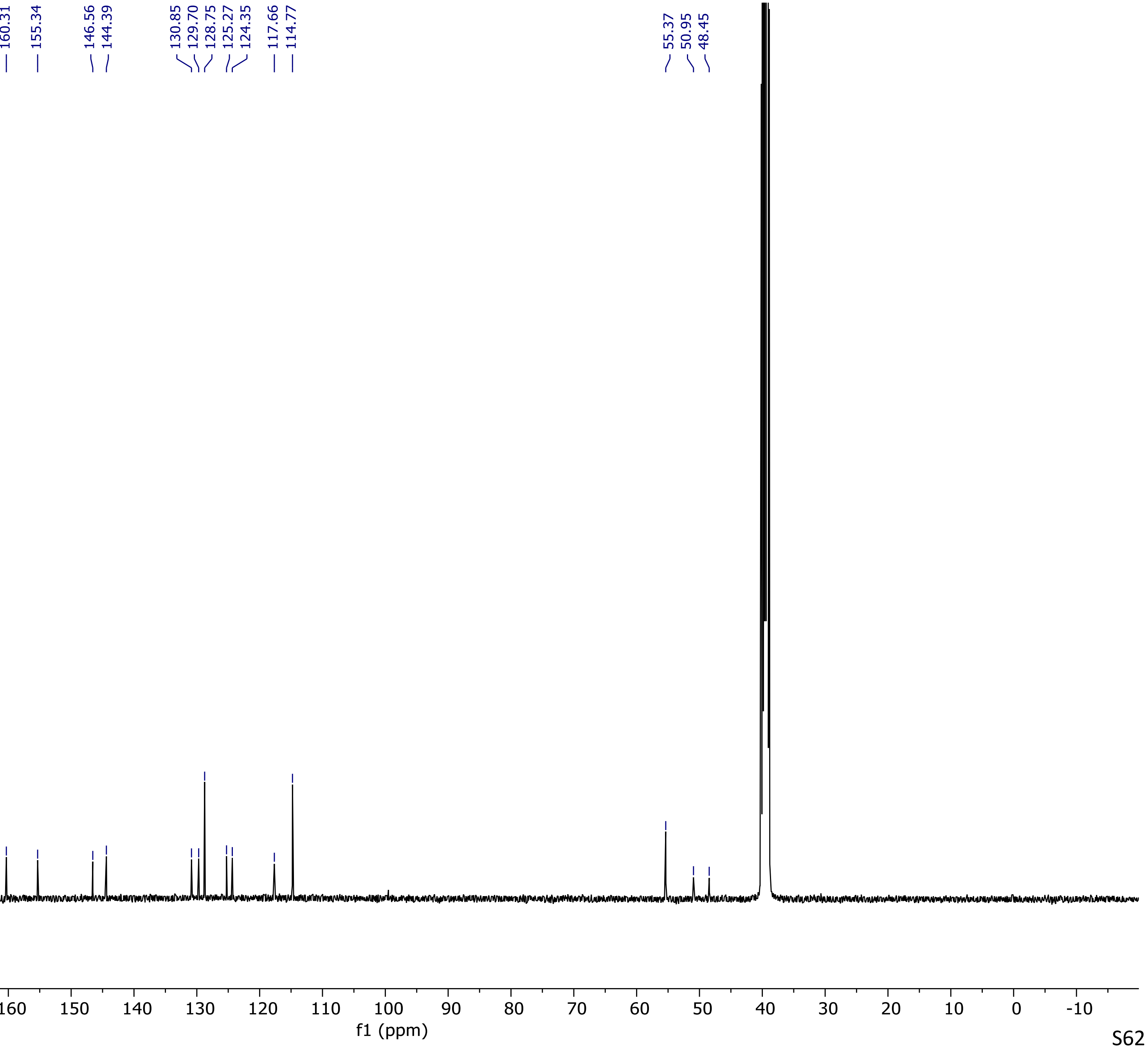

^{1}H NMR (500 MHz, CD_3OD)

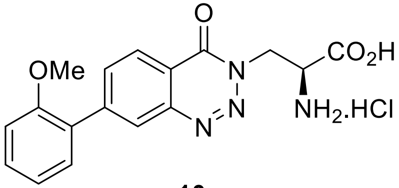

10e

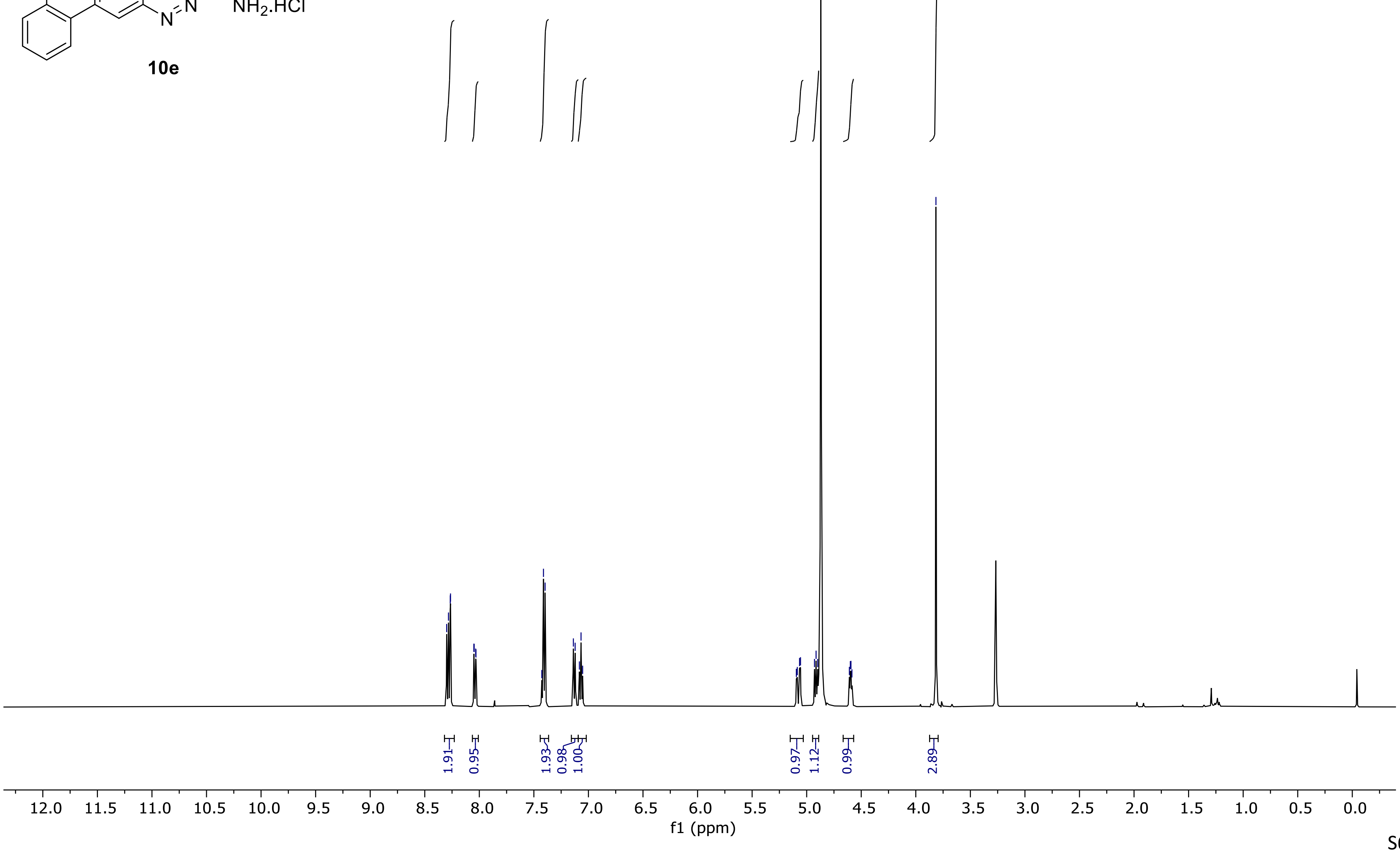

$^{13}C\{^{1}H\}$ NMR (126 MHz, CD_3OD)

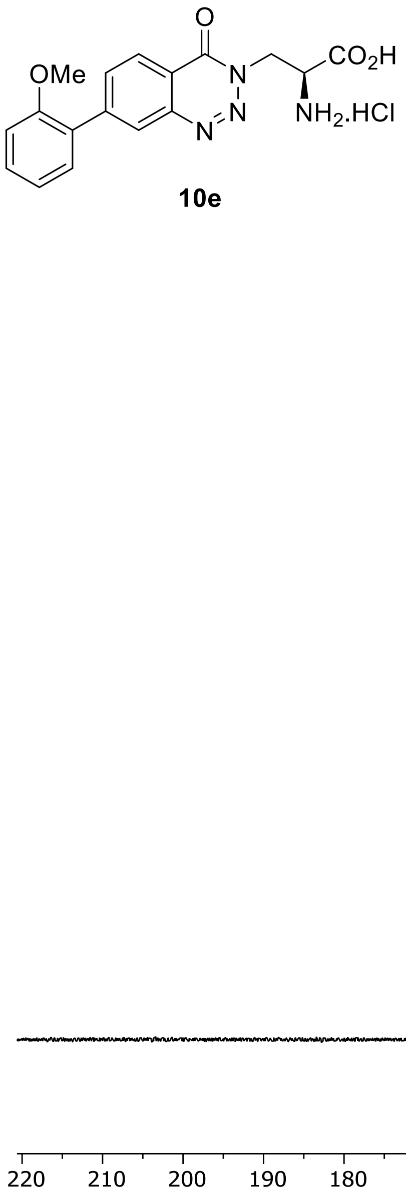

10e

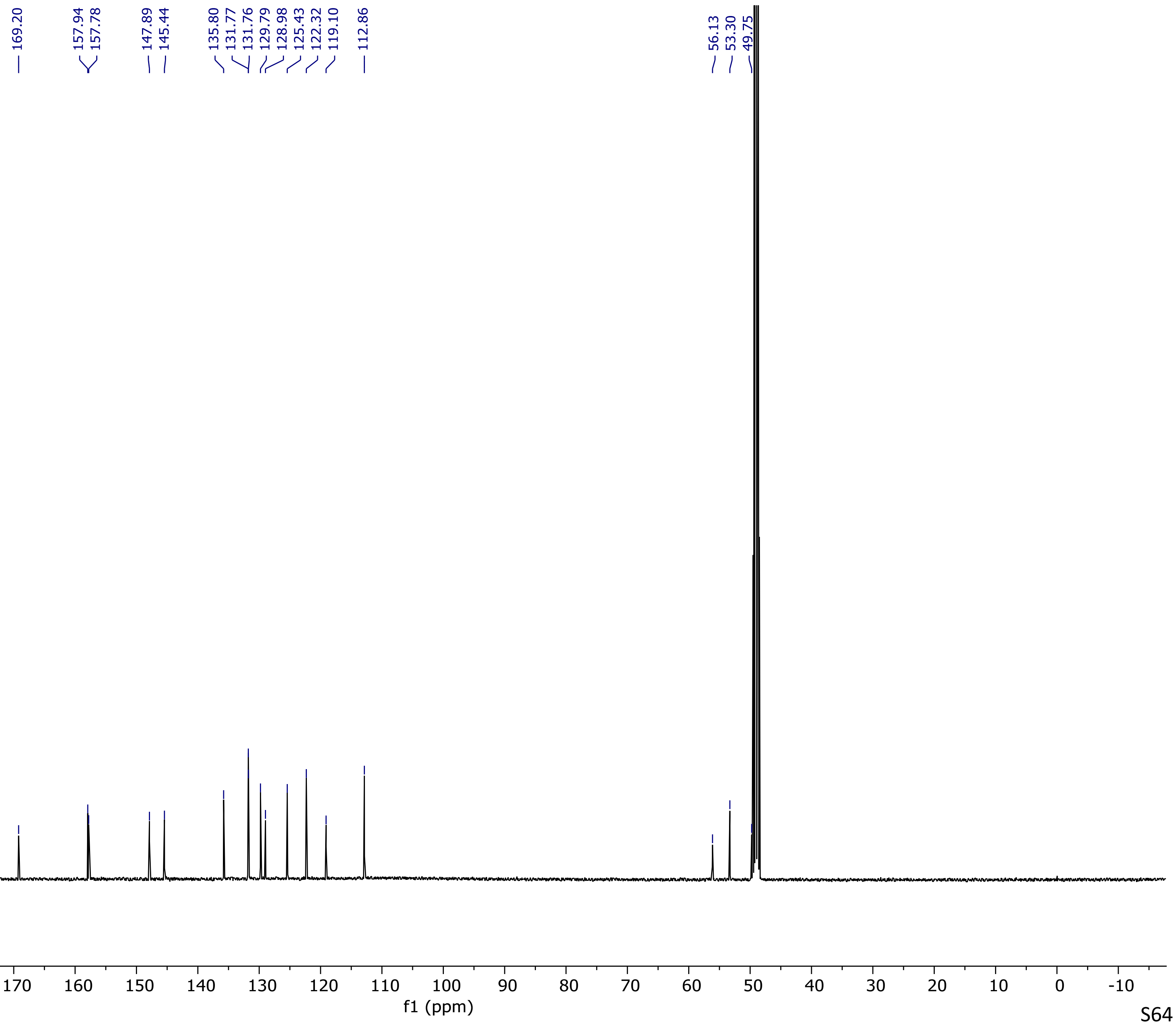

^{1}H NMR (400 MHz, DMSO-d_6)

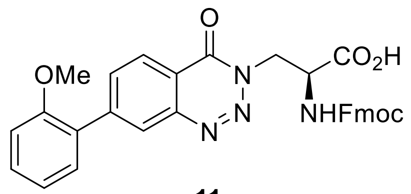

11

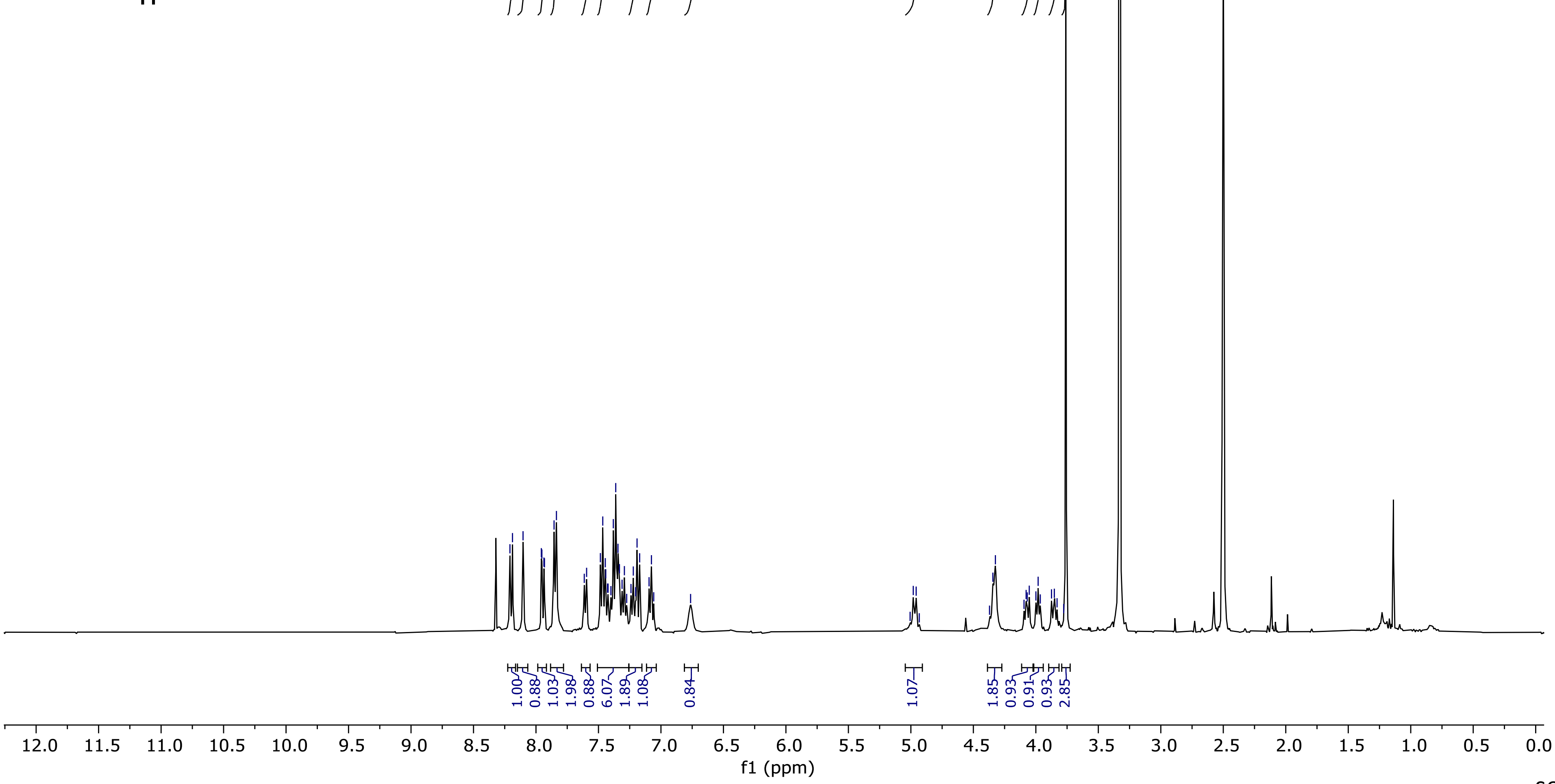

Supplement: Supplementary file 1 — ol3c02112_si_001.pdf [file ol3c02112_si_001.pdf]
